# IMU-based rotator cuff injury recognition with varying configurations and combinations

**DOI:** 10.3389/fphys.2026.1794238

**Published:** 2026-07-06

**Authors:** Wanwan Xiong, Xianwu Zeng, Jianning Sun, Yuexin Zhang, Chun Pei, Yi-Hao Chan, Peng Chen, Yunru Ma

**Affiliations:** 1 College of Computer and Cyber Security, Fujian Normal University, Fuzhou, China; 2 Department of Orthopedics, The First Affiliated Hospital, Fujian Medical University, Fuzhou, China; 3 School of Medicine and Health Science, Guangzhou Huashang College, Guangzhou, China; 4 Department of Transportation Engineering, Xiamen City University, Xiamen, China; 5 Department of Rehabilitation Medicine, School of Health, Fujian Medical University, Fuzhou, China

**Keywords:** deep learning, inertial measurement unit, machine learning, upper limb functional movement, rotator cuff injury recognition, multi-action combination

## Abstract

**Introduction:**

Assessing functional movements is important for evaluating shoulder impairments, as these movements directly reflect patients’ capacity to perform daily activities. Intelligent rotator cuff injury (RCI) recognition is beneficial for clinical management.

**Methods:**

This study aimed to develop accurate and cost-effective RCI recognition models by integrating machine learning (ML)/deep learning (DL) algorithms with upper limb kinematic data collected in functional movement, and to explore optimal motion task combinations and model configurations for clinical practice. A total of 102 participants were prospectively enrolled, comprising 51 patients diagnosed with RCI, 25 patients diagnosed with adhesive capsulitis and 26 healthy volunteers. Patients with adhesive capsulitis were included to assess model’s ability to distinguish RCI from other shoulder disorders that present with similar function limitations. Each participant performed six functional movements simulating daily activities and two shoulder range of motion (ROM) tests. Upper limb kinematic data were collected by inertial measurement units (IMUs) and analyzed via eight classification models, including the proposed DL based RCI recognition model (RCIRNet) and seven conventional ML models (e.g., KNN, SVM, DT, RF, NB, AdaBoost, and XGBoost). Given the relatively small sample size, data augmentation was applied to mitigate overfitting and improve model generalization. Model performance was evaluated across single and combined motion tasks via Accuracy, Precision, Recall, F1-score, and AUC with its 95% confidence interval (CI).

**Results:**

Results demonstrated that shoulder frontal ROM test alone achieved the best performance with RCIRNet, yielding an Accuracy of 0.89, F1-score of 0.89, and AUC of 0.93 (95%CI, 0.88–0.98). Combining four tasks increased Recall to 1.0 with high level Accuracy, F1-score, and AUC. However, adding more movements (5–8 tasks) did not improve model performance. Among different model configurations, RCIRNet performed best, SVM demonstrated overall stability, and KNN excelled in Recall.

**Discussion:**

It is concluded that selecting discriminant movements is more critical for effective RCI recognition than simply increasing the quantity of movement tasks. The shoulder frontal ROM test combined with RCIRNet provides an optimal approach for accurate and efficient clinical screening and rehabilitation monitoring.

## 1 Introduction

Rotator cuff injury (RCI) is a common clinical condition seen by orthopedic surgeons. Without timely diagnosis and intervention, RCI can lead to restricted shoulder joint mobility, chronic pain, weakness and even irreversible functional impairment, severely impacting patients’ quality of life and occupational capacity (May and Garmel, 2025). The diagnosis of RCI relies on surgeons integrating clinical features, physical examinations, and medical images to reach a comprehensive conclusion (Roy et al., 2015; Longo et al., 2025). It also requires careful differentiation from other shoulder pathologies with similar symptoms, such as adhesive capsulitis (Mezian et al., 2026). However, the interpretation of physical examinations can be influenced by clinicians’ personal experience and subjective judgement, leading to inter-rater variability (Schmidt et al., 2021). Meanwhile, medical imaging modalities (e.g., magnetic resonance imaging and ultrasonography) are costly and time-consuming, constraining their accessibility for large-scale screening and early intervention. These issues highlight an urgent need for the development of accurate and cost-effective RCI recognition tools, especially of ones that are capable of discriminating RCI from other clinically similar conditions.

With the integration of artificial intelligence (AI) in medical engineering, smart recognition technologies based on machine learning (ML) and deep learning (DL) have introduced innovative and promising solutions for RCI diagnoses. ML technology, characterized by highly efficient feature extraction and classification capabilities, serves as a primary method for shoulder disorder identification (Zhao et al., 2024; Jung et al., 2025). Some conventional ML models for shoulder disorder recognition typically rely on manual or semi-automatic feature engineering, and then the extracted features are usually processed by a classifier for recognition (Liu et al., 2024; Sassi et al., 2025). In contrast, DL is advantageous in automatically extracting high-level abstract features without manual feature engineering, demonstrating strong capability in handling complex medical data (Sarker, 2021). However, most of current AI approaches rely on medical image data (Ghosh et al., 2025; Li et al., 2025; Wu et al., 2025), which is less convenient for capturing functional movements in real clinical scenarios. Furthermore, the high computational complexity of image-based models constrains their clinical applicability. In contrast, IMU-based wearable devices are low-cost, easy to operate, and appropriate for continuous recording of upper-limb kinematics during functional movement tasks.

Inertial measurement units (IMUs), characterized by compact size, high portability, and ease of operation, offer an attractive measurement approach for clinicians and researchers (Esser et al., 2009; Cocco et al., 2024; Jocham et al., 2024). While IMU data-driven AI models have been widely applied in abnormal movement analysis and recognition (Liu et al., 2024, 2025; Audisio et al., 2025), existing studies show a concentration on neurological disorders, overlooking their potential in musculoskeletal conditions such as RCI (Smits Serena et al., 2025). Since the primary role of upper limbs is to maintain activities of daily life (ADLs), careful analysis of upper limb kinematics in ADLs is crucial for RCI screening, diagnosis, and treatment outcome assessment. Previous studies have analyzed upper limb kinematics in ADL functional motion tasks in patients with RCI (Vidt et al., 2016; Pataky et al., 2021; Schnorenberg et al., 2022). In addition to ADLs, standardized shoulder range of motion (ROM) tests are routinely used in clinical examinations to evaluate shoulder function. However, even the limited studies that attempt to use IMUs tend to rely on kinematic features extracted from an individual movement, without systematically investigating how a specific motion task or how various movement combinations influence model performance (Lee et al., 2024; Sassi et al., 2025). This gap reveals a lack of methodological evidence for selecting the most effective shoulder movement in real clinical scenarios. Consequently, developing IMU-based AI models specially for RCI recognition would not only broaden the applicational spectrum of assistive technologies in musculoskeletal care, but also rigorously define the motion task-model efficacy relationship.

Research objectives of this study are twofold. First, we aim to evaluate and compare the performance of various DL/ML approaches when distinguishing RCI. The models used IMU data collected in six ADL functional movement and two ROM tests. Second, we aim to explore the most accurate and efficient motion task combination for RCI recognition. By addressing these two mentioned aims, this study is designed to establish methodological foundations for optimizing IMU-based RCI recognition schemes, streamlining clinical data collection process, and ultimately facilitating rapid screening and intelligent rehabilitation monitoring.

## 2 Related work

### 2.1 IMU-based shoulder activity recognition

IMU-based activity recognition has been increasingly investigated in upper-limb and shoulder movement assessment. A previous study has demonstrated that wearable inertial sensors can recognize upper limb activities of daily living with high sensitivity and high specificity, including drinking, eating, and brushing hair (Lemmens et al., 2015). In this study, thirty healthy adults and one stroke patient performed standardized upper limb activities. Sensors were placed on the dominant hand, wrist, upper arm, and chest, each of which contained a triaxial accelerometer, gyroscope, and magnetometer. The activities were identified by extracting multi-array signal templates, decomposing activities into sub-phases, and performing pattern recognition with a two-dimensional convolution.

In shoulder rehabilitation settings, IMU has been used to identify exercise repetitions and classify therapeutic movements (Brennan et al., 2020; Liu et al., 2023). Brennan et al. developed a ML-based segmentation technique to accurately segment shoulder rehabilitation exercises using IMU data with a mean overall accuracy of 0.871. Thirty-five healthy adults performed 11 functional shoulder exercises with 31 variants while wearing three IMUs on the wrist, arm, and scapula. The system involved a convolution classifier and Finite State Machine to detect repetitions and movement phases. Liu et al. studied twenty healthy participants performing six frozen shoulder rehabilitation exercises, including finger walk, interlock, pendulum, shoulder abduction, shoulder flexion with the stick, and towel stretch. Five IMUs on the wrists, upper arms, and chest recorded triaxial accelerometer and gyroscope data at 128 Hz. Signals were segmented to overlapping sliding windows, and eight statistic features were extracted. Different ML models (k-nearest neighbors, support vector machines, and naive bayes) and DL models (convolutional neural network and gated recurrent unit) were trained to classify exercise types, achieving a high performance with an accuracy of 0.956. Collectively, these studies suggest that IMU-based recognition can support remote rehabilitation monitoring and structured shoulder exercise assessment.

### 2.2 IMU-based movement disorder detection

IMUs have also been increasingly used for movement disorderdetection and functional impairment assessment (Sassi et al., 2024; Cerfoglio et al., 2023). In shoulder related disorders, Kwak et al. investigated the dynamic motion quality of shoulders with rotator cuff tears using IMU (Kwak et al., 2020). The study enrolled 24 patients and compared the affected shoulder with the intact contralateral shoulder. Three new parameters (number of peaks, peak velocity to mean velocity ratio, and number of sign reversals) were defined to quantify motion smoothness, varied with tear size and correlated with symptom duration. A previous study has evaluated shoulder function in adhesive capsulitis using two IMUs placed on the wrist and upper arm (Chang et al., 2022). Three basic shoulder movements such as flexion, extension, and abduction were recorded. Eight statistical features were extracted from the accelerometer signals, including mean, standard deviation, variance, maximum, minimum, range, kurtosis, and skewness. Statistical analysis revealed significant difference between patients and healthy subjects, especially for standard deviation.

Similar IMU-based approaches have also been applied to evaluate upper limb function in stroke patients. Martino Cinnera et al. systematically reviewed the use of IMUs for upper limb assessment in stroke patients, analyzing 35 studies with 475 patients (Martino Cinnera et al., 2024). Kinematic parameters derived from IMU, including joint angles, velocity, acceleration, smoothness, and symmetry, showed moderate strong correlations with clinical scales such as Fugl-Meyer Assessment (FMA) scale, Action Research Arm Test (ARAT) and Modified Ashworth Scale (MAS). It demonstrated that IMU provided reliable and objective assessment for upper limb function.

### 2.3 Shoulder disorder recognition

Previous studies have explored shoulder disorder recognition using different modalities. For x-ray-based recognition (Hashimoto et al., 2024; Iio et al., 2024), Iio et al. developed a DL screening tool based on convolutional neural network (CNN) for detecting rotator cuff tears from shoulder radiographs, achieving an AUC of 0.82. Hashimoto et al. proposed a CNN model for automated detection and classification of rotator cuff tears on plain shoulder radiographs, reporting an accuracy of 0.86 and an AUC of 0.88.

Ultrasound-based methods have also been explored (Ho et al., 2022; Ghosh et al., 2025). Ho et al. evaluated CNN for classifying rotator cuff tears in ultrasound image, achieving an accuracy of 0.88 and an AUC of 0.83. Ghosh et al. proposed a deep reinforcement learning-based ultrasound video summarization and detection framework. This framework was used for rotator cuff tears detection from ultrasound videos, achieving an accuracy of 0.85.

MRI-based approaches have been further developed for rotator cuff tears recognition (Esfandiari et al., 2023; Guo et al., 2023). Guo et al. developed a DL model for automatic diagnosis of supraspinatus tears from MRI images, with a F1-score of 0.82 and an AUC of 0.92. Esfandiari et al. designed a novel CNN for detecting rotator cuff tears from MRI images, achieving an accuracy of 0.92.

In addition to imaging-based methods, Liu et al. (2024) proposed an IMU-based ML framework for frozen shoulder recognition from daily activities, including washing hair, washing upper back, washing lower back, placing an object on a high shelf, and removing an object from the back pocket. This framework achieved an accuracy of 0.88 and a F1-score of 0.89.

In summary, previous studies have demonstrated the feasibility of IMU for upper limb activity recognition, shoulder rehabilitation monitoring, and movement quality assessment. However, most existing works focus on recognizing movement types, quantifying kinematics, or monitoring rehabilitation, rather than identifying RCI from clinically relevant shoulder movements. Furthermore, previous studies for shoulder disorder recognition have focused on imaging data, and RCI detection using IMU data remains largely unexplored. In addition, the roles of different functional movements and models remain insufficiently explored. Therefore, this study aims to evaluate multiple functional shoulder tasks and ML/DL models for RCI recognition.

## 3 Materials and methods

### 3.1 Participants

This study was reviewed and approved by the Biomedical Research Ethics Committee of Fujian Medical University (Approval No. [2023]455) and was registered in the Chinese Clinical Trial Registry (Registration No. ChiCTR2300076496). Written informed consent was obtained for all participants prior to their inclusion in this study. A total of 102 subjects were prospectively enrolled at the First Affiliated Hospital of Fujian Medical University between October 2023 and December 2024. Subjects were categorized into two groups: patients with RCI and without RCI (NRCI).

The inclusion criteria for RCI group were as follows: patients with preoperative MRI- or arthroscopy-confirmed rotator cuff tear and with the functional ability to accomplish designed motion tasks. The exclusion criteria were as follows: diagnosis of other shoulder disorders or neuromuscular disorders; severe impairment affecting data collection.

The inclusion criteria for the NRCI group were as follows: individuals without any shoulder neuromusculoskeletal disorders, or patients with MRI-confirmed adhesive capsulitis; functional ability to accomplish designed motion tasks. The exclusion criteria were as follows: presence of RCI or other shoulder disorders; diagnosis of neuromuscular disorders.

### 3.2 Data collection

**Figure 1:**
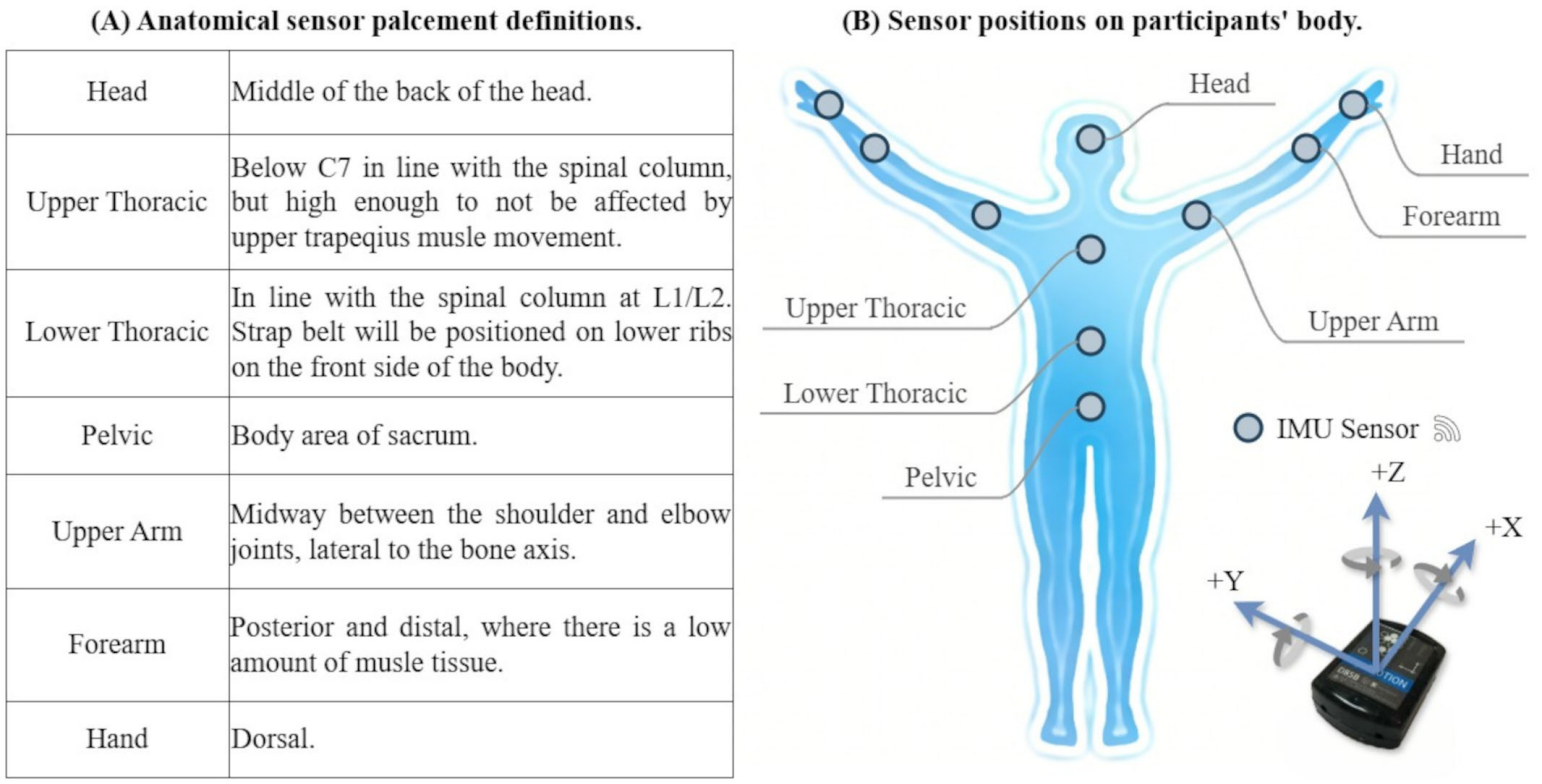
IMU sensor placement. **(A)** is definitions of anatomical locations of sensor placement. **(B)** is overview of sensor positions on participants’ body.

An IMU motion capture system (Portable Lab 02, Noraxon Inc., Scottsdale, AZ, USA) was employed to record upper limb kinematic data. Ten wearable IMU sensors were positioned on participants’ head, hands, forearms, upper arms, upper thoracic, lower thoracic and pelvic. All sensors were fixed by elastic straps. Sensor placement is shown in **Figure 1**.

Each IMU sensor was equipped with a triaxial accelerometer (± 16 g), gyroscope (± 2000°/s), and magnetometer (± 4900 µT) to capture linear acceleration, angular velocity, and magnetic field signals along x, y, and z axes. Data were collected at a sampling frequency of 100 Hz. The IMU system underwent a zero-calibration procedure before each test session to reset gyros of all assigned sensors.

**Figure 2:**
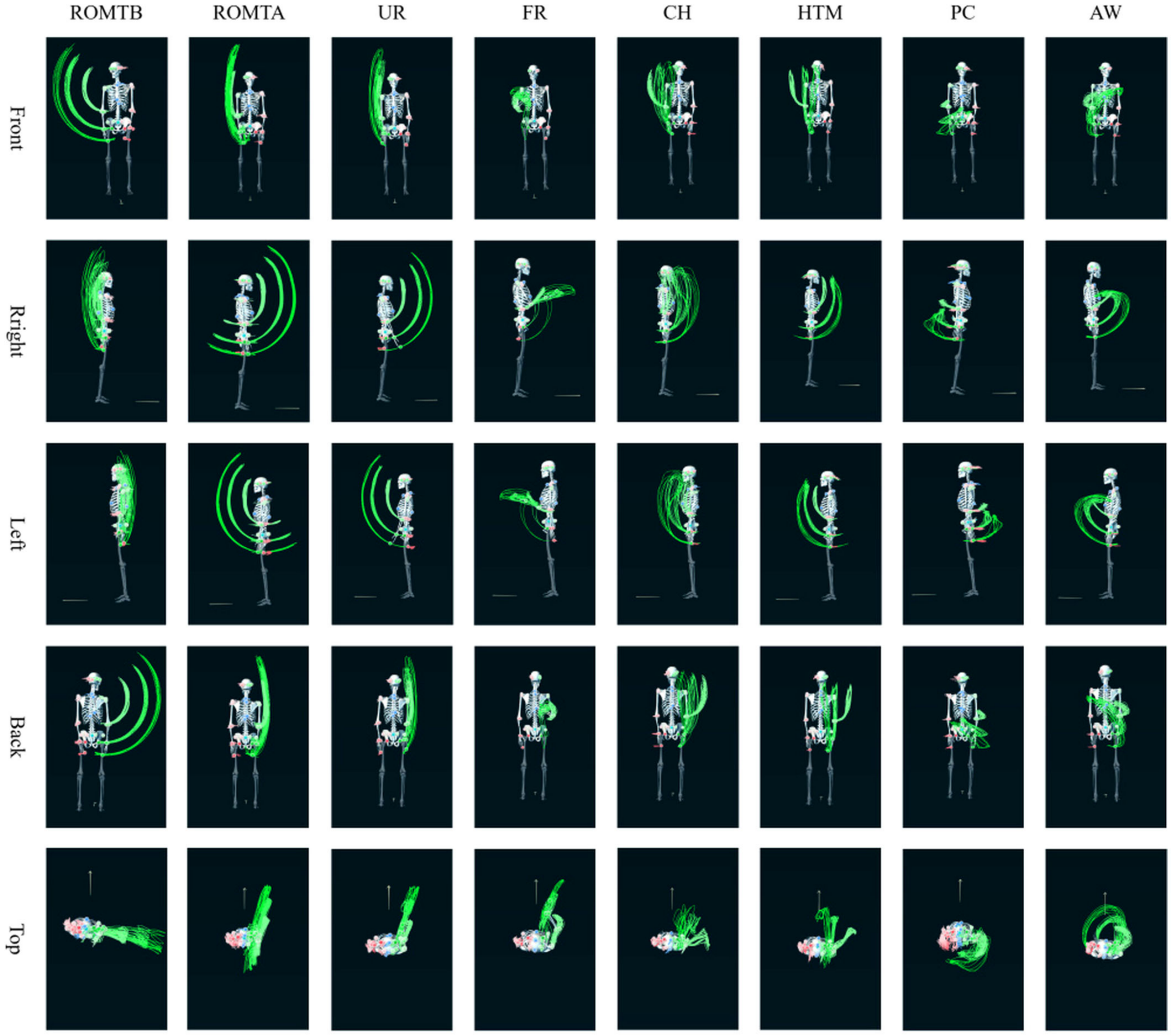
The diagram of eight shoulder joint functional assessment movements from front, right, left, back and top views. These Eight movements were denoted by AW, PC, HTM, CH, FR, UR, ROMTA and ROMTB, respectively (please refer to **Table 1** for details).

Experimental protocol consisted of two phases. The first phase included sensor placement and calibration. The static calibration was conducted to establish upper limb segmental coordinate systems before movement data collection. The second phase included movement data acquisition. Each participant was asked to stand straight with all joints in a neutral position and palms aligned with the sagittal plane. Then the participant performed six standardized shoulder movement tasks (Pichonnaz et al., 2015) at a self-comfortable speed, including movements simulating axilla wash, perineal care, hand to mouth, combing hair, forward and upward reach with 1kg item, as well as shoulder sagittal and frontal range of motion (ROM) test. Subsequently, participants returned their arms to the start position. Each movement was repeated five times. Detailed movement description is shown in **Table 1** and five views of eight actions are shown in **Figure 2**. These functional motion tasks were selected based on previous biomechanical studies assessing upper limb kinematics for patients with RCI (Vidt et al., 2016; Pataky et al., 2021; Schnorenberg et al., 2022).

**Table 1:** Eight selected shoulder motions and their functional description.

Motion name	Motion instructions
Axilla Wash (AW)	Patients started with the arm in an anatomical and neutral position, and reached across the torso to touch the contralateral shoulder. This motion mimics washing axilla.
Perineal Care (PC)	Patients started with the arm in an anatomical and neutral position, and reached behind their torso and touch the middle of lumbar with the palm side. This motion mimics perineal care.
Hand to Mouth (HTM)	Patients started with the arm in an anatomical and neutral position, and moved up to their mouth. This motion mimics drinking water or eating.
Combing Hair (CH)	Patients started with the arm in an anatomical and neutral position, and moved to their forehead and over their head to neck. This motion mimics washing and combing hair.
Forward Reach with an 1kg item (FR)	Patients started with the elbow flexed to 90° and upper arm parallel to the torso, then forward reach as far as possible with an 1 kg item. This motion mimics cleaning a desk.
Upward Reach with an 1kg item (UR)	Patients started with the elbow flexed to 90° and upper arm parallel to the torso, then upward reach as far as possible with an 1 kg item. This motion mimics taking an item from a cupboard.
Shoulder sagittal ROM test (ROMTA)	Patients were asked to flex/extend the shoulder at their best.
Shoulder frontal ROM test (ROMTB)	Patients were asked to adduct/abduct the shoulder at their best.

### 3.3 Dataset construction and partitioning

Only acceleration data was selected as model input because it can capture dynamic upper-limb motion patterns, while reducing the dimensionality and processing complexity (Kim et al., 2022). For participants with RCI or adhesive capsulitis, upper thoracic, upper arm and forearm sensor data of the affected side were used, while for participants with normal shoulders, upper thoracic, upper arm and forearm sensor data of the dominant side were used. All possible combinations of the selected eight actions were evaluated, ranging in size from one to eight actions ( $\left(C\;=\;\sum_{k=1}^{8}(k_{8}^{)}\right)\;$. For multiple actions, first extract the features of different actions respectively, then concatenate the extracted features and send them to the classifier for final classification.

Acceleration signals, with manually annotated start and end points, were processed through wavelet denoising and Kalman filtering (Wang et al., 2024). Individual variations were eliminated via normalization (Min-Max) and Z-score standardization, with uniformized sampling lengths (4500 length). A subject-wise split strategy was adopted, ensuring all samples from any single participant were assigned exclusively to either training, validation, or test set. Data were divided into training, validation, and test sets at a 7:1:2 ratio using stratified sampling to maintain class balance. Within NRCI group, stratification was also applied to preserve comparable proportions of healthy controls and patients with adhesive capsulitis across the three data sets. Data labels followed the format “Diagnosis_SubjectID”. In addition, Leave-One-Subject-Out (LOSO) cross-validation was performed to further evaluate generalizability to unseen participants. Due to the large number of action combinations and the computational cost of repeated LOSO model training, LOSO validation was conducted on single-action scenario. In each LOSO fold, all samples from one participant were held out as the test set, and the remaining participants were used for model training and validation.

### 3.4 Machine learning-based RCI recognition

**Figure 3:**
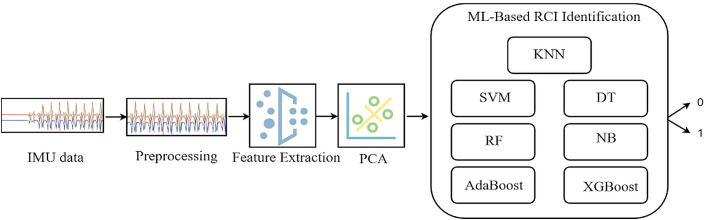
The proposed framework for ML-based RCI recognition. Raw IMU data was preprocessed, and time-domain statistical features and kinematic features were extracted. Dimensionality reduction was conducted via principal component analysis (PCA). Seven ML models were employed for RCI identification.

Seven machine learning algorithms, including k-nearest neighbors (KNN), support vector machines (SVM), decision trees (DT), random forests (RF), naive bayes (NB), adaptive boosting (AdaBoost), and extreme gradient boosting (XGBoost), were adopted in this study (**Figure 3**).

Time-domain statistical features and kinematic features were extracted from preprocessed data. Time-domain statistical features comprised eight metrics calculated from seven-dimensional acceleration data (three axes, total acceleration, and three plane components), including mean, standard deviation, variance, maximum value, minimum value, range, kurtosis, and skewness (Renuka et al., 2025). Kinematics features (e.g., smoothness and velocity) were extracted from triaxial acceleration signals, including the number of mean crossovers (NMPC), number of peaks (NP), spectral arc length (SPARC), logarithm of log-normal jerk (LDLJ), and power index (PI) (Balasubramanian et al., 2012; de los Reyes-Guzmán et al., 2014; Melendez-Calderon et al., 2021). Extracted features were normalized and subjected to principal component analysis (PCA) for dimensionality reduction (retaining 95% variance) before being fed into the model (Abdi and Williams, 2010).

### 3.5 Deep learning-based RCI recognition (RCIRNet)

**Figure 4:**
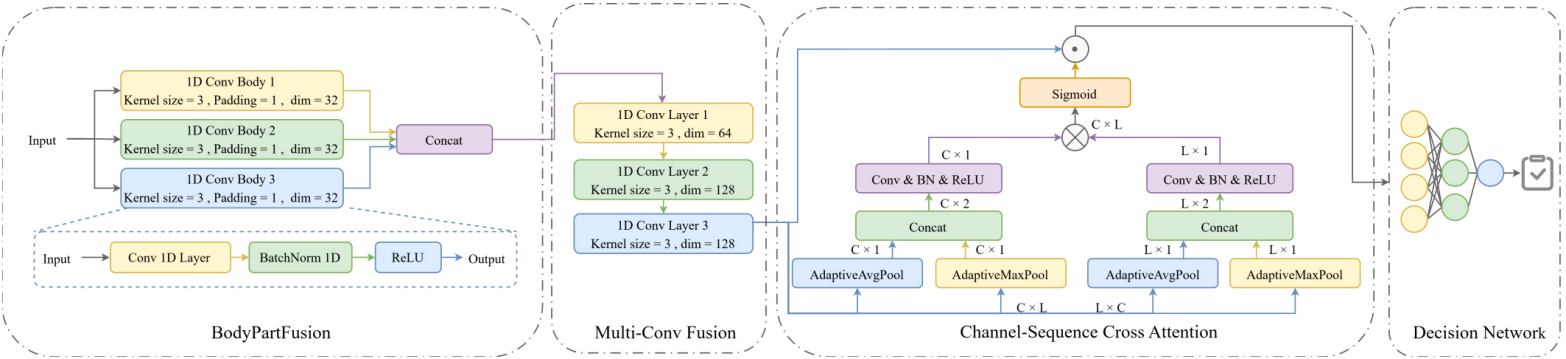
The proposed RCIRNet framework for RCI recognition. RCIRNet consisted of four modules: BodyPartFusion Module, Multi-Conv Fusion Module, Channel-Sequence Cross Attention Module and Decision Network Module.

A deep learning-based model was proposed to distinguish RCI from NRCI (**Figure 4**). To enhance model’s generalization performance and alleviate overfitting, several data augmentation strategies, including addition of Gaussian noise (standard deviation 0.05), temporal distortion (standard deviation 0.2), and amplitude scaling (range 0.9–1.1), were applied to the training data set (Uchitomi et al., 2023). Processed IMU data was progressively transformed through a hierarchical architecture, which consisted of a BodyPartFusion module, a Multi-Conv Fusion module, a Channel Sequence Cross Attention module and a Decision Network module.

BodyPartFusion Module: IMU sequences from three body regions were processed by separate standard one-dimensional (1D) convolution (Conv) branches to extract local temporal features. Each branch using a 1D convolution layer with a kernel size of 3, a padding of 1, and an output feature dimension of 32. Each layer was combined with batch normalization (BatchNorm, BN) and rectified linear unit (ReLU). BN normalized features for improving training stability and convergence speed. ReLU introduced nonlinearity and alleviate vanishing gradient problem. These temporal features were then concatenated and fused via a linear layer. This two-stage module design was capable of obtaining information from individual body region, while simultaneously preserving inter-segmental coordination patterns during movement.

Multi-Conv Fusion Module: This module consisted of three 1D convolutional layers with feature dimensions of 64, 128, and 128, respectively. Each layer used a kernel size of 3. This design could build layered feature representations. It also progressively improved the ability to describe complex movement patterns and changes in movement caused by disease.

Channel Sequence Cross Attention (CSCA) Module: This module consisted of adaptive average pooling (AdaptiveAveragePool), adaptive max pooling (AdaptiveMaxPool), standard convolutional layers, and sigmoid activation. First, average and maximum pooling were applied to extract global features from channel and sequence dimensions, respectively. These features were then concatenated via standard convolutional layers. Sigmoid functions subsequently normalized attention weights. By cross-fusing attention information from both channel and sequence dimensions, CSCA dynamically adjusted the weights of input features and highlighted key spatiotemporal characteristics, enhancing RCIRNet’s RCI classification performance. The mathematical formulation of CSCA is:


$$F_{out}=F⊙\sigma (g([AvgPool_{T}(F),MaxPool_{T}(F)])\cdot g([AvgPool_{C}(F),MaxPool_{C}(F)])),$$

where F was input feature, ⊙ denoted element-wise multiplication, σ(·) was the sigmoid activation function, and g(·) denoted the mapping function implemented by one-dimensional convolution.

Decision Network: Features refined by the CSCA module were fed into Decision Network, which learned decision boundary through three fully connected layers and nonlinear activation functions. The numbers of neurons in the three layers are 128, 64, and 2. Proper regularization was implemented to reduce overfitting. Finally, a Softmax layer was introduced to automatically generate RCI classification results.

### 3.6 Implementation details

To compare recognition performance across different action combinations, each action combination was trained and tested separately. All experiments were implemented in Python 3.9.23 and PyTorch 2.1.0 framework. Model training was conducted on a workstation equipped with an Intel Core i9-13900k CPU, 16.0 GB of memory size, NVIDIA GeFore RTX 4090 GPU. Training parameters were set as: batch size 16, learning rate 0.005, momentum factor 0.88, total training epochs 200.

### 3.7 Evaluation indicators

To assess models’ classification performance, five metrics, including Accuracy, Precision, Recall, F1-score, and the area under the ROC curve (AUC) with its 95% confidence interval (CI), were computed. These metrics were calculated based on TP, FN, FP and TN. In particular, TP was RCI samples correctly predicted as RCI; FN was RCI samples incorrectly predicted as NRCI; FP was NRCI samples incorrectly predicted as RCI; TN was NRCI samples correctly predicted as NRCI.

Accuracy equals the proportion of correctly predicted samples among all samples, reflecting model’s overall prediction performance. It is calculated as


$$Accuracy=\frac{TP+TN}{TP+TN+FP+FN}$$

Precision computes the proportion of correctly predicted positive samples among all samples, reflecting model’s ability to control false positive. It is expressed as


$$Precision=\frac{TP}{TP+FP}$$

Recall refers to the proportion of correctly predicted positive samples among all samples predicted as positive, reflecting the sensitivity of model. It is calculated as


$$Recall=\frac{TP}{TP+FN}$$

F1-score is the harmonic mean of Precision and Recall, balancing the trade-off between the two metrics. It can be expressed as


$$F1-score=2\times \frac{Precision\times Recall}{Precision+Recall}$$

AUC measures a model’s ability to distinguish between positive and negative classes. The ROC curve plots false positive rate (FPR) on horizontal axis and true positive rate (TPR) on vertical axis at various threshold settings. The AUC value ranges from 0 to 1, with 1 suggesting perfect discrimination and 0.5 revealing a random classifier.


$$FPR=\frac{FP}{FP+TN},TPR=\frac{TP}{TP+FN}$$

To quantify the overall performance difference between RCIRNet and ML models across action combination scenarios, we conducted a task-level aggregated comparison. For each scenario, the AUC difference was calculated as $\;\Delta AUC=AUC_{RCIRNet}-AUC_{ML}$. The mean and 95%CI of difference across all scenario were estimated using bootstrap resampling. Wilcoxon signed-rank test was employed to access whether the difference differed from zero.

We conducted an interpretability analysis, based on saliency maps, for the proposed model. For each sample, the gradient of the target class score with respect to the input sequence was computed. Then, the absolute gradients across feature dimensions were averaged. This produced the importance of each time step for the prediction. The full movement cycle was divided into three phases to analyze the importance of different phases, including early phase (0%–33%), middle phase (33%–67%), and late phase (67%–100%). The saliency of each channel was averaged over the time dimension. The channels were then grouped by sensor to evaluate the relative contribution of each sensor. To statistically compare sensor contributions, a Kruskal-Wallis test was performed to evaluate the overall difference among the three sensors, followed by *post hoc* tests with Holm correction for pairwise comparisons, where statistical significance was defined as p<0.05.

To further evaluate the effectiveness of the proposed RCIRNet, additional comparisons with five representative DL baseline models were conducted under the representative action ROMTB. These five baselines were CNN, long short-term memory (LSTM), CNN-LSTM, temporal convolutional neural (TCN), and Transformer. The implementation details of these baseline models were provided in **Supplementary Material**.

## 4 Results

This section presented the results for RCI recognition based on IMU data collected in eight shoulder functional motions. RCI recognition performance across a progression from single motions to multi-motion combinations using eight ML and DL models were systematically evaluated. For conciseness, only the full results of single motion and the best and second-best results for each step of multi-motion were reported in this section, which were based on a comprehensive evaluation of five metrics. Complete set of results for all models and motion combinations could be found in Supplementary Material (**Supplementary Figures 1–36**). The results of LOSO validation and DL comparison experiment also could be found in Supplementary Material (**Supplementary Tables 1**, **2**).

### 4.1 Demographic characteristics

Demographic characteristics were demonstrated in **Table 2**. The RCI group consisted of 51 patients with RCI (15 male and 36 female). The NRCI group consisted of 25 patients with adhesive capsulitis and 26 healthy controls (27 males and 24 females). The distribution of gender and height between RCI group and NRCI group showed a significant difference with a p value of 0.016 and 0.015, respectively. But there was no difference exhibited in age with a p value of 0.054. In addition, there was no statistically significant difference in weight or BMI between the two group.

**Table 2:** Demographic characteristics of study participants.

Characteristic	Category	RCI group (n=51)	NRCI group (n=51)	Statistics	p-value
Gender, n (%)	Male	15 (29.4%)	27 (52.9%)	χ2 = 5.829	0.016
	Female	36 (70.6%)	24 (47.1%)		
Age, years	Mean ± SD	58 ± 8.10	54 ± 11.74	t = 1.95	0.054
Height, cm	Mean ± SD	161.16 ± 69.45	165.12 ± 61.39	t = –2.75	0.0151
Weight, kg	Mean ± SD	60.32 ± 94.34	62.68 ± 92.90	t = –1.234	0.220
BMI, kg/m^2^	Mean ± SD	23.06 ± 5.70	22.81 ± 7.58	t = 0.494	0.622

Chi-square test was used to analyze the difference in gender and t-test was used to analyze the difference in age, height, weight, and BMI between RCI group and NRCI group. A statistical significance was defined as p<0.05.

### 4.2 RCI recognition in single-action scenario

**Figure 5:**
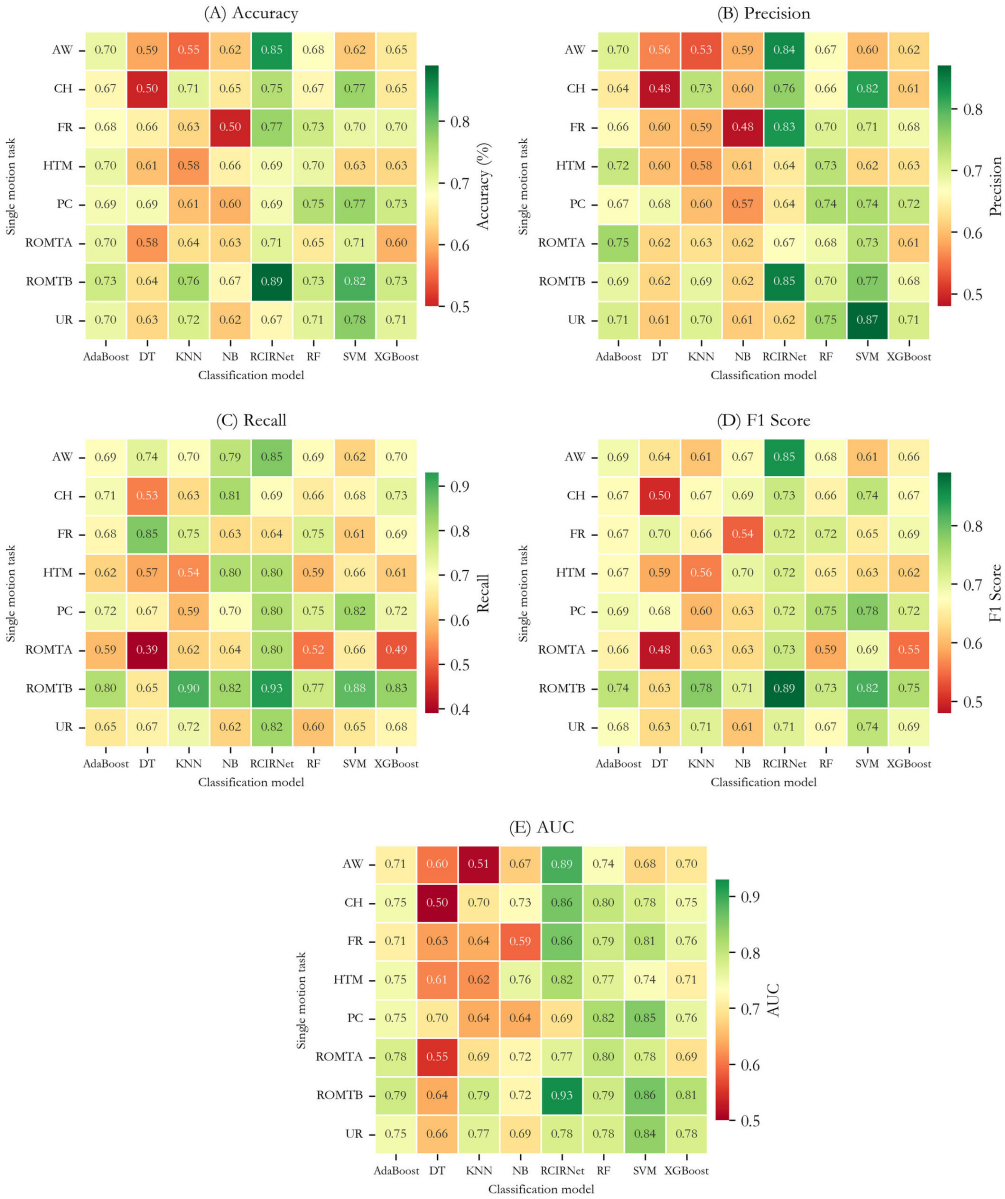
Evaluation metrics at eight single-action scenarios with eight models. **(A–E)** is Accuracy, Precision, Recall, F1-score and AUC, respectively.

The recognition performance of single motion tasks was demonstrated in **Figure 5**. Shoulder frontal ROM (ROMTB) task yielded the best performance when processed by RCIRNet (Accuracy = 0.89, F1-score = 0.89, and AUC = 0.93), indicating ROMTB was the most discriminative single motion for RCI recognition. AW ranked the second best (Accuracy = 0.85, F1-score = 0.85, and AUC = 0.90), suggesting its potential as an effective alternative. Furthermore, RCIRNet outperformed all traditional ML models in single motion scenarios.

### 4.3 RCI recognition in dual-action scenario

As shown in **Table 3**, the classification performance of dual-action tasks decreased compared with single actions. ROMTB_HTM processed by RCIRNet demonstrated the best performance with an Accuracy of 0.83 and F1-score of 0.83, indicating strong complementary effects between these two actions. ROMTB_UR showed the second-best performance with an Accuracy of 0.83, F1-score of 0.83 and AUC of 0.90 using RCIRNet.

**Table 3:** Evaluation metrics of the best and second-best performance at 28 dual-action scenarios with eight models.

Performance Rank	Metric	Action	Model	Value
Best	Accuracy	ROMTB_HTM	RCIRNet	0.83
	Precision	ROMTB_UR	RCIRNet	0.85
	Recall	ROMTA_CH	KNN	0.97
	F1-score	ROMTB_HTM	RCIRNet	0.83
	AUC (95%CI)	ROMTB_UR	RCIRNet	0.90 (0.84,0.95)
Second-Best	Accuracy	ROMTB_UR	RCIRNet	0.83
	Precision	UR_HTM	XGBoost	0.84
	Recall	FR_HTM	KNN	0.95
	F1-score	ROMTB_UR	RCIRNet	0.83
	AUC (95%CI)	ROMTB_HTM	RCIRNet	0.90 (0.83,0.95)

95%CI, 95% Confidence Interval.

### 4.4 RCI recognition in three-action scenario

As shown in **Table 4**, model performance significantly improved when compared with dual-action scenarios. Notably, the ROMTB_FR_HTM combination processed by RCIRNet yielded the best performance (Accuracy = 0.86, F1-score = 0.86, and AUC = 0.92). The maximum Recall was investigated in KNN across all three-action conditions (Recall = 0.95). Among the second-best evaluations, FR_AW_HTM processed by RCIRNet was superior with an Accuracy of 0.84 and F1-score of 0.84.

**Table 4:** Evaluation metrics of the best and second-best performance at 56 three-action scenarios with eight models.

Performance Rank	Metric	Action	Model	Value
Best	Accuracy	ROMTB_FR_HTM	RCIRNet	0.86
	Precision	ROMTB_FR_HTM	RCIRNet	0.88
	Recall	ROMTA_UR_FR	KNN	0.95
	F1-score	ROMTB_FR_HTM	RCIRNet	0.86
	AUC (95%CI)	ROMTB_FR_HTM	RCIRNet	0.92 (0.87,0.97)
Second-Best	Accuracy	FR_AW_HTM	RCIRNet	0.84
	Precision	UR_AW_HTM	XGBoost	0.86
	Recall	ROMTA_CH_HTM	KNN	0.95
	F1-score	FR_AW_HTM	RCIRNet	0.84
	AUC (95%CI)	ROMTB_FR_CH	RCIRNet	0.90 (0.85,0.96)

95%CI, 95% Confidence Interval.

### 4.5 RCI recognition in four-action scenario

As shown in **Table 5**, the performance of four-action combinations improved compared to three-action results. ROMTB_UR_FR_CH with RCIRNet generated the highest Accuracy (0.88) and F1-score (0.88). ROMTB_ROMTA_UR_AW with RCIRNet ranked the second with an Accuracy of 0.87 and F1-score of 0.86. Notably, KNN model demonstrated the best Recall (1.00) with the ROMTA_PC_FR_HTM combination, indicating the minimum false negatives.

**Table 5:** Evaluation metrics of the best and second-best performance at 70 four-action scenarios with eight models.

Performance Rank	Metric	Action	Model	Value
Best	Accuracy	ROMTB_UR_FR_CH	RCIRNet	0.88
	Precision	ROMTB_UR_FR_CH	RCIRNet	0.89
	Recall	ROMTA_PC_FR_HTM	KNN	1.00
	F1-score	ROMTB_UR_FR_CH	RCIRNet	0.88
	AUC (95%CI)	ROMTB_UR_FR_AW	RCIRNet	0.94 (0.90,0.98)
Second-Best	Accuracy	ROMTB_ROMTA_UR_AW	RCIRNet	0.86
	Precision	ROMTB_ROMTA_UR_AW	RCIRNet	0.87
	Recall	ROMTA_PC_FR_CH	KNN	0.98
	F1-score	ROMTB_ROMTA_UR_AW	RCIRNet	0.86
	AUC (95%CI)	ROMTB_UR_CH_AW	RCIRNet	0.93 (0.88,0.97)

95%CI, 95% Confidence Interval.

### 4.6 RCI recognition in five-action scenario

Results for five-action combinations indicated a performance decline compared to few action combinations (**Table 6**). RCIRNet continued to be the most reliable model with the ROMTB_ROMTA_PC_CH_AW combination, attaining the highest value in Accuracy (0.85), F1-score (0.85) and AUC (0.92). While the KNN model again was the most sensitive model with the ROMTB_ROMTA_UR_PC_AW combination (Recall = 1.00). ROMTB_ROMTA_UR_AW_HTM processed by RCIRNet ranked the second with an Accuracy of 0.85 and F1-score of 0.85.

**Table 6:** Evaluation metrics of the best and second-best performance at 56 five-action scenarios with eight models.

Performance Rank	Metric	Action	Model	Value
Best	Accuracy	ROMTB_ROMTA_PC_CH_AW	RCIRNet	0.85
	Precision	ROMTB_ROMTA_PC_CH_AW	RCIRNet	0.86
	Recall	ROMTB_ROMTA_UR_PC_AW	KNN	1.00
	F1-score	ROMTB_ROMTA_PC_CH_AW	RCIRNet	0.85
	AUC (95%CI)	ROMTB_ROMTA_PC_CH_AW	RCIRNet	0.92 (0.87,0.97)
Second-Best	Accuracy	ROMTB_ROMTA_UR_AW_HTM	RCIRNet	0.85
	Precision	ROMTB_ROMTA_PC_FR_HTM	RCIRNet	0.85
	Recall	ROMTA_PC_FR_CH_HTM	KNN	0.98
	F1-score	ROMTB_ROMTA_UR_AW_HTM	RCIRNet	0.85
	AUC (95%CI)	ROMTB_ROMTA_FR_CH_HTM	RCIRNet	0.91 (0.86,0.96)

95%CI, 95% Confidence Interval.

### 4.7 RCI recognition in six-action scenario

As shown in **Table 7**, a performance regression was observed in six-action conditions. The ROMTB_ROMTA_UR_PC_CH_AW combination with RCIRNet produced the best Accuracy (0.82), F1-score (0.82) and AUC (0.90). ROMTB_ROMTA_PC_FR_CH_HTM with RCIRNet posed the second with an Accuracy of 0.81 and F1-score of 0.81. KNN continued to excel in sensitivity with a Recall of 0.98.

**Table 7:** Evaluation metrics of the best and second-best performance at 28 six-action scenarios with eight models.

Performance Rank	Metric	Action	Model	Value
Best	Accuracy	ROMTB_ROMTA_UR_PC_CH_AW	RCIRNet	0.82
	Precision	ROMTB_ROMTA_UR_PC_CH_AW	RCIRNet	0.84
	Recall	ROMTA_PC_FR_CH_AW_HTM	KNN	0.98
	F1-score	ROMTB_ROMTA_UR_PC_CH_AW	RCIRNet	0.82
	AUC (95%CI)	ROMTB_ROMTA_UR_PC_CH_AW	RCIRNet	0.90 (0.85,0.96)
Second-Best	Accuracy	ROMTB_ROMTA_PC_FR_CH_HTM	RCIRNet	0.81
	Precision	ROMTB_PC_FR_CH_AW_HTM	RCIRNet	0.84
	Recall	ROMTB_ROMTA_PC_FR_CH_HTM	KNN	0.98
	F1-score	ROMTB_ROMTA_PC_FR_CH_HTM	RCIRNet	0.81
	AUC (95%CI)	ROMTB_PC_FR_CH_AW_HTM	RCIRNet	0.90 (0.85,0.95)

95%CI, 95% Confidence Interval.

### 4.8 RCI recognition in seven-action scenario

Compared to fewer action scenarios, results of classification exhibited a decline in seven-action conditions (**Table 8**). ROMTB_UR_PC_FR_CH_AW_HTM delivered the best Accuracy of 0.81 and F1-score of 0.81 with RCIRNet. ROMTB_ROMTA_UR_PC_FR_CH_HTM achieved the second-best performance under RCIRNet, obtaining an Accuracy of 0.79 and F1-score of 0.79. KNN exhibited the highest Recall (0.97), indicating its sensitivity in detecting positive samples.

**Table 8:** Evaluation metrics of the best and second-best performance at eight seven-action scenarios with eight models.

Performance Rank	Metric	Action	Model	Value
Best	Accuracy	ROMTB_UR_PC_FR_CH_AW_HTM	RCIRNet	0.81
	Precision	ROMTB_UR_PC_FR_CH_AW_HTM	RCIRNet	0.84
	Recall	ROMTB_ROMTA_UR_PC_FR_CH_HTM	KNN	0.97
	F1-score	ROMTB_UR_PC_FR_CH_AW_HTM	RCIRNet	0.81
	AUC (95%CI)	ROMTB_ROMTA_UR_PC_FR_CH_AW	RCIRNet	0.89 (0.83,0.94)
Second-Best	Accuracy	ROMTB_ROMTA_UR_PC_FR_CH_HTM	RCIRNet	0.79
	Precision	ROMTB_ROMTA_UR_PC_FR_AW_HTM	RCIRNet	0.81
	Recall	ROMTB_ROMTA_PC_FR_CH_AW_HTM	KNN	0.95
	F1-score	ROMTB_ROMTA_UR_PC_FR_CH_HTM	RCIRNet	0.79
	AUC (95%CI)	ROMTB_ROMTA_UR_PC_CH_AW_HTM	RCIRNet	0.86 (0.79,0.93)

95%CI, 95% Confidence Interval.

### 4.9 RCI recognition in eight-action scenario

Consistent with the trend investigated from above action combinations, overall metrics revealed a continue decrease in the final eight-action scenarios (**Table 9**). RCIRNet achieved the best overall performance, with an Accuracy of 0.78, F1-score of 0.74 and AUC of 0.84. SVM performed the second best, with an Accuracy of 0.75 and F1-score of 0.74. KNN maintained the highest Recall (0.97), reflecting strong sensitivity in identifying positive samples.

**Table 9:** Evaluation metrics of the best and second-best performance at one eight-action scenarios with eight models.

Performance Rank	Metric	Model	Value
Best	Accuracy	RCIRNet	0.78
	Precision	RCIRNet	0.80
	Recall	KNN	0.97
	F1-score	RCIRNet	0.78
	AUC (95%CI)	RCIRNet	0.84 (0.77,0.91)
Second-Best	Accuracy	SVM	0.75
	Precision	SVM	0.75
	Recall	RCIRNet	0.78
	F1-score	SVM	0.74
	AUC (95%CI)	AdaBoost	0.82 (0.75,0.90)

95%CI, 95% Confidence Interval.

### 4.10 Summaries

**Figure 6:**
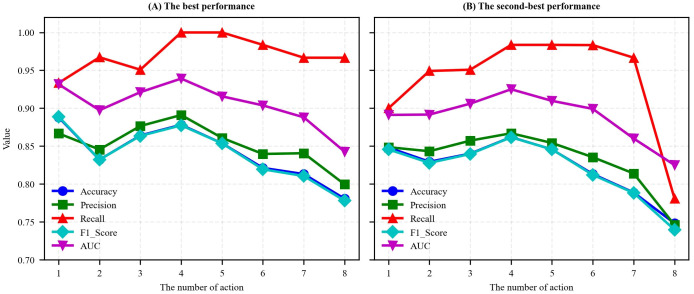
The best and second-best performance of RCI recognition from actions one to eight. This image shows the trend of identification performance as the number of action increases. **(A)** is the best performance of all action combinations, and **(B)** is the second best performance.

The best and second-best results across all action combinations and all classification models were depicted in **Figure 6**. Single action and a moderate number of 3–4 actions achieved an optimal balance, ensuring high Recall while maintaining good Accuracy, F1-score, and AUC. In contrast, performance declined when the number of actions increased (5–8 actions), indicating that redundant kinematic features and noise may weaken models’ discriminative power.

### 4.11 Model difference analysis

To further quantify the magnitude of performance difference, we calculated AUC differences between RCIRNet and seven ML models across all action combination scenarios (**Table 10**). RCIRNet showed consistently positive AUC differences compared to the seven ML models. Wilcoxon signed-rank test showed that the AUC difference was significant different from zero, with all p-value less than 0.001.

**Table 10:** Across action combination analysis of AUC difference between RCIRNet and seven ML models.

Model	Mean	Median	95% CI	Wilcoxon_Statistic	P_Value
AdaBoost	0.02	0.02	(0.01, 0.03)	10220	<0.001
DT	0.20	0.19	(0.19, 0.21)	1	<0.001
KNN	0.15	0.17	(0.14, 0.17)	784	<0.001
NB	0.10	0.10	(0.09, 0.10)	1083	<0.001
RF	0.03	0.04	(0.02, 0.04)	8316	<0.001
SVM	0.02	0.02	(0.01, 0.02)	11265	<0.001
XGBoost	0.03	0.03	(0.02, 0.04)	8641	<0.001

### 4.12 Ablation experiment

The ablation experiment was conducted using the representative action ROMTB which achieved the best performance in all action combinations. We evaluated the effect of data augmentation and architectural modules in **Table 11**. Model variants were conducted by removing or replacing key modules of RCIRNet. These variants were used to assess the contribution of the BodyPartFusion module, Multi-Conv Fusion module, CSCA module and Decision Network. NoBodyPartFusion was removed the BodyPartFusion module, NoMultiConvFusion was removed the Multi-Conv Fusion module, NoCSCA was removed the CSCA module, and SimpleClassifier was obtained by replaying the original multi-layer classifier with single linear layer. The results demonstrated that all modules contributed to model performance. And the use of data augmentation improved Accuracy from 0.77 to 0.89, F1-score from 0.77 to 0.89 and AUC from 0.86 to 0.93.

**Table 11:** Ablation analysis of data augmentation and architectural modules.

Model	Accuracy	Precision	Recall	F1-score	AUC (95% CI)
RCIRNet	0.89	0.85	0.93	0.89	0.93 (0.88, 0.98)
NoBodyPartFusion	0.74	0.70	0.82	0.75	0.84 (0.77, 0.91)
NoMultiConvFusion	0.76	0.71	0.85	0.77	0.82 (0.74, 0.89)
NoCSCA	0.77	0.83	0.65	0.73	0.85 (0.78, 0.92)
SimpleClassifier	0.80	0.89	0.80	0.79	0.88 (0.82, 0.94)
NoAugmentation	0.77	0.74	0.8	0.77	0.86 (0.79, 0.92)

95%CI, 95% Confidence Interval.

The performance of sensor configurations also was analyzed, and the results can be found in **Table 12**. Three sensors gained the optimal Accuracy of 0.89, Recall of 0.93 and F1-score of 0.93. Upper arm and forearm sensors configuration achieved the best Precision of 0.89 and AUC of 0.95. In addition, single sensor configurations all performed relatively poorly, indicating that multi-sensor information is beneficial for RCI recognition.

**Table 12:** Recognition performance of different sensor configurations on the ROMTB action.

Configuration	Accuracy	Precision	Recall	F1-score	AUC (95% CI)
All_Three_Sensors	0.89	0.85	0.93	0.89	0.93 (0.88, 0.98)
Upper_Arm_Forearm	0.85	0.89	0.78	0.83	0.95 (0.92, 0.98)
Upper_Thoracic_Upper_Arm	0.62	0.62	0.55	0.58	0.74 (0.65, 0.83)
Upper_Thoracic_Forearm	0.70	0.67	0.73	0.70	0.74 (0.66, 0.83)
Upper_Thoracic_Only	0.65	0.67	0.53	0.59	0.74 (0.65, 0.82)
Upper_Arm_Only	0.70	0.64	0.87	0.74	0.70 (0.60, 0.80)
Forearm_Only	0.58	0.55	0.73	0.63	0.69 (0.60, 0.79)

95%CI, 95% Confidence Interval.

### 4.13 Model interpretability analysis

**Figure 7:**
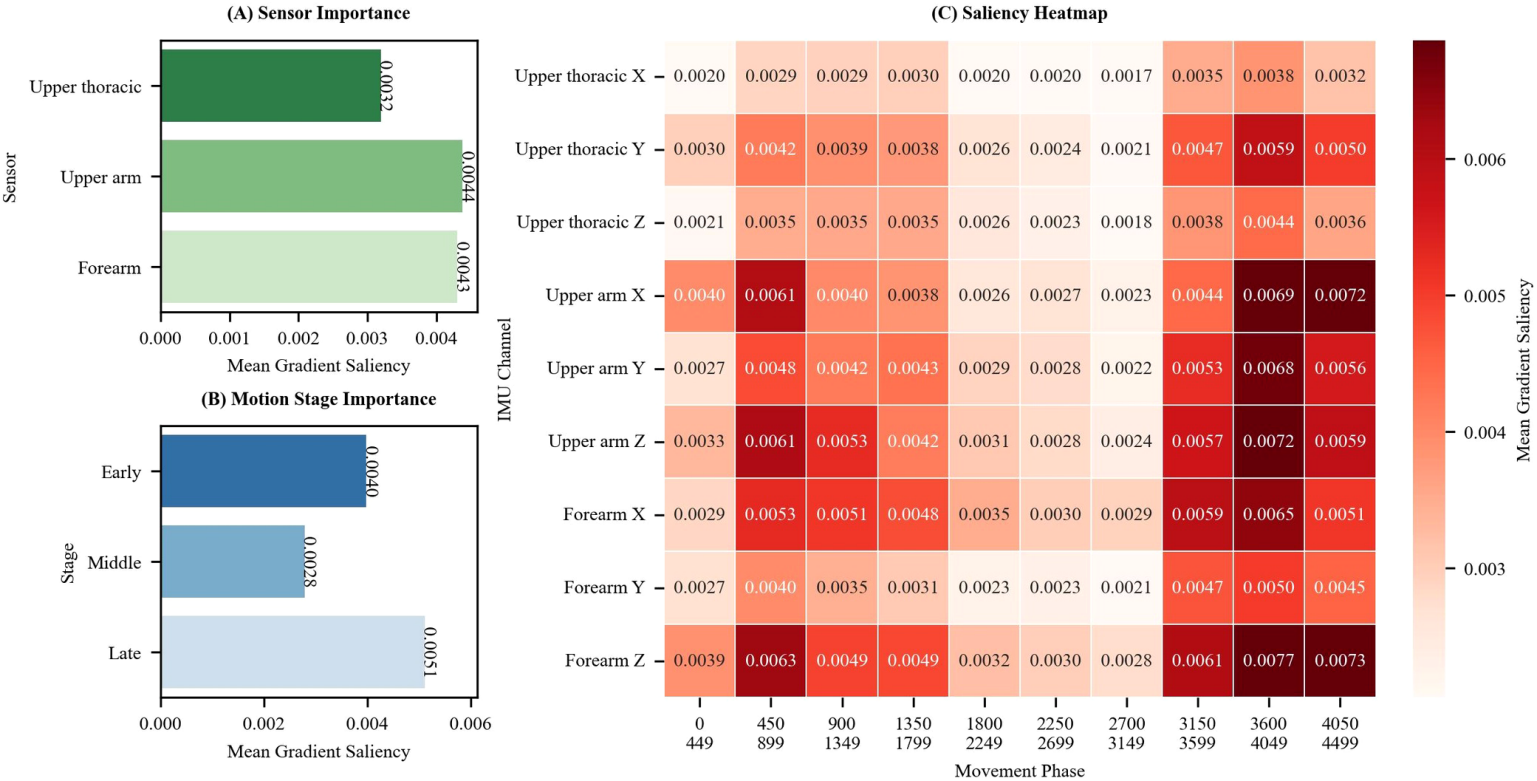
Interpretability analysis of sensor and temporal importance based on saliency. **(A)** compares the importance of sensor locations, **(B)** summarizes saliency across motion stages, and **(C)** presents the temporal saliency heatmap across IMU channels.

We employed model interpretability analysis to show which phase of the motion and which sensor contributed most to the classification (**Figure 7**; **Table 13**). This experiment was conducted on the single motion ROMTB using a gradient-based saliency method. Mean saliency scores were 0.0044 for upper arm sensor, 0.0043 for forearm sensor, and 0.0032 for upper thoracic sensor. The Kruskal-Wallis test showed a significant overall difference among the three sensors (H = 24.361, p < 0.001). *Post hoc* test comparisons indicated no significant difference between the upper arm and forearm sensors (p = 0.888). In contrast, both the upper arm and the forearm sensors showed significantly higher saliency than the upper thoracic sensor (both p < 0.001). The saliency map showed higher importance in the final 75%–100% of the IMU sequence, indicating that the late phase contributed most to model classification.

**Table 13:** Pairwise comparisons of sensor-level gradient saliency scores using a *post hoc* test (n = 125 per sensor).

Comparison	Mean	Median	95% CI	Adjusted p-value
Upper arm vs Forearm	<0.001	<0.001	[-0.000596, 0.000745]	0.888
Upper arm vs Upper thoracic	0.001	0.001	[0.000594, 0.001757]	<0.001
Forearm vs Upper thoracic	0.001	0.001	[0.000544, 0.001691]	<0.001

A statistical significance was defined as Adjusted p-value < 0.05. 95% CI, 95% Confidence Interval.

## 5 Discussions

This study systematically assessed recognition performance of eight DL/ML models from a single motion task to multiple motion task combinations (2–8 motion tasks). Multi-dimensional metrics were evaluated, including Accuracy, Precision, Recall, F1-score, and AUC 95%CI. Results indicated that overall recognition effectiveness is jointly determined by discriminative power and complementarity of selected motion tasks, as well as the adaptability of models. The combination of action shoulder frontal ROM test and RCIRNet was the best configuration for effective RCI recognition.

### 5.1 Analysis at the model level

Different models exhibit distinct advantages across various evaluation metrics. RCIRNet demonstrates outstanding performance in all action scenarios, capable of deeply mining complex features. SVM demonstrates the most stable performance compared to other traditional ML, indicating its strong generalization capability and robustness. KNN demonstrates outstanding Recall performance, ensuring an extremely low false negative rate and proving highly suitable for large scale clinical screening applications.

The architecture of RCIRNet influenced model performance by improving its ability to learn multi-segment movement patterns from IMU signals. The Multi-Conv Fusion module was used to extract local patterns from IMU sequences. BodyPartFusion module and CSCA module were design to enhance informative features from the upper thoracic, upper arm and forearm signals. However, additional modules may also increase the risk of overfitting with a limited sample size. To reduce this risk, global average pooling and global maximum pooling were applied to obtain compact pooled features before classification, and the dropout was applied during training. The comparison with DL baselines further supported the effectiveness of RCIRNet. The experimental result was placed in the **Supplementary Material**. RCIRNet achieved the highest Accuracy, Precision, F1-score, and AUC. TCN achieved the highest Recall, but its Precision was lower. This indicates that RCIRNet provided a better balance between sensitivity and positive prediction reliability. Data augmentation was also used to increase the effective training samples. The ablation analysis further showed that removing the key modules affected model performance. It suggests that these modules contributed to model discriminative ability rather than merely increase model complexity. Nevertheless, the limited sample size remains an important limitation, and the generalizability of the proposed architecture should be further validated in larger sample size.

The data augmentation ablation experiment showed that RCIRNet trained with augmentation outperformed without augmentation, suggesting that data augmentation improved model robustness under the limited sample size. The improvement was related to the increase in effective training samples and the greater diversity of samples within the same group. This helped reduce the model’s dependence on specific subject signal fluctuations. However, data augmentation should be employed cautiously on biomechanical IMU data. If applied inappropriately, augmentation may introduce artificial noise or alter meaningful movement information. In this study, the improved performance suggested that the augmentation strategy did not substantially disrupt the discriminative movement information related to RCI.

As shown in **Table 13**, the upper arm and forearm sensors contributed similar to model prediction, and both outperformed the upper thoracic sensor. This result implies that RCI mainly affect shoulder and elbow inter-joint coordination, whereas the upper thoracic motion reflects trunk compensation is less discriminant for RCI recognition. Sensor configuration analysis was conducted to identify the most appropriate set of IMU sensors that achieved both high recognition performance and practical simplicity. Although the two-sensor setup (upper arm combined with forearm) achieved higher AUC and Precision, the three-sensor configuration provided a clear superiority in Recall. Recall is an essential metric for the intended screening application, because missing a true RCI case carries greater risk. Given this trade-off, we recommended the three-sensor set as the preferred balance between clinical utility and discriminative ability, while the two-sensor setup remains a complementary tool for scenarios that prioritize precision and overall recognition ability. The result demonstrated that the upper arm and forearm sensors contributed similar to model prediction, while both contributed more than the upper thoracic sensor.

For a clinical screening tool, it is important to understand the rationale behind the model’ prediction of RCI. We conducted model interpretability experiments to investigate this. Our results revealed that the late phase (75–100% of the motion cycle) contributed most to the RCI recognition. During this phase, the arm is lowering back to the start position, where the rotator cuff acts as a dynamic stabilizer of the humeral head. In patients with RCI, tendon tears lead to impaired eccentric control and trunk compensations. Consistently, our results showed that the y-axis (anterior-posterior) channel of the upper thoracic sensor exhibited higher saliency scores in the late phase compared to the early and middle phases, reflecting compensatory trunk motions.

### 5.2 Analysis at the motion task selection level

Shoulder frontal ROM test integrated with RCIRNet demonstrates the most outstanding performance in single-motion scenarios, achieving optimal results across four core evaluation metrics (Accuracy, Recall, F1-score, AUC). Shoulder frontal movement ability inherently possesses rich discriminative information. It can expose weakness, instability, and compensatory movement patterns related to RCI. Biomechanical studies have shown that complete supraspinatus tears can reduce abduction force and increase superior humeral head migration (Rybalko et al., 2020). In addition, shoulders with rotator cuff tears require compensatory deltoid function to prevent abduction motion loss (Dyrna et al., 2018). The performance of axilla wash (AW) task ranked secondary. Previous biomechanical studies have investigated that patients with RCI exhibited higher minimum elevation angles and smaller joint contact forces in the AW task compared to healthy controls, implying this task has potential for RCI discrimination (Vidt et al., 2016, 2018). In multi-motion scenarios, model performance exhibited an improvement when 2–3 motion tasks were included. Upon further expansion to 4 tasks, performance gradually approached a saturation plateau. A slight decline was observed when including 5 to 8 motion tasks. It should be noted that overall model performed better in single-motion scenarios rather than multi-motion settings, suggesting that selecting informative motion tasks is more important than increasing motion numbers.

Although functional tasks are clinically important, they did not outperform standardized ROM tasks in this study. This may be due to the higher complexity and variability of daily functional movements. Functional movements involve coordinated movement across multiple upper-limb segments rather than isolated single plane shoulder motion. Previous studies have shown that daily functional tasks require coordinated glenohumeral and scapulothoracic motion. And patients with rotator cuff tears may perform them with altered thoracohumeral kinematics, including greater internal rotation (Rundquist et al., 2009; Vidt et al., 2016). Patients may complete the same tasks with different compensatory strategies, which may reduce the consistency of discriminative movement patterns. Symptomatic rotator cuff tear patients may develop compensatory movement patterns, including abnormal activity and of the biceps brachii and posterior deltoid muscles (Veen et al., 2021). Therefore, daily functional movements should be interpreted as complementary to ROM tasks. ROM tasks are more suitable for maximizing screening performance, whereas functional movements provide meaningful information about daily functional impairment and compensatory. Future models may benefit from explicit temporal segmentation of functional movement, phase-level attention mechanisms, and multi-modal IMU inputs to better capture informative patterns in complex functional movements.

### 5.3 Action-model matching relationship

Experimental results reveal the optimal matching rules between motions and models. ROMTB with RCIRNet or SVM yielded robust outcomes in applications that requires a balance model performance and complexity. Multi-action setting including ROMTA with KNN reaches the highest Recall, making it a preferable solution where missing a true positive case is not acceptable. The ROMTB_UR_FR_CH combination with RCIRNet reaches the best scores in Accuracy, F1-score and AUC, thereby representing the strongest configuration in multi-motion setting.

### 5.4 Clinical application value

Findings arising from this study offer a potential solution for effective and accurate RCI recognition schemes. Appropriate motion combinations can significantly improve Recall while maintaining high accuracy, thereby facilitating early screening and large-scale population surveillance. Compared to conventional imaging examinations, approaches adopted in this study applied motion data collected by wearable IMU sensors, offering advantages of convenience, low cost, and scalability. Thus, they can serve as a complementary tool for imaging examinations, providing physicians with rapid diagnostic references. The clinical meaning of model performance depends on the intended application scenarios. In community or primary care, Recall is important because false-negative predictions may delay further examination or treatment. KNN with multi-action including ROMTA is recommended in this scenario. In specialist outpatient assessment, especially when differentiating RCI from other shoulder disorders such as adhesive capsulitis, AUC is more relevant because it reflects the overall discriminative ability of the model. RCIRNet with shoulder frontal ROM test is suitable for this scenario. In rehabilitation or follow-up monitoring, Precision may be important because clinicians need reliable identification of abnormal or recovered movement patterns before adjusting rehabilitation plans. ROMTB_UR_FR_CH action combination with RCIRNet is appropriate in this scenario. Therefore, different metrics provide complementary information for different clinical needs. Most existing shoulder disorder recognition studies rely on imaging data. Although these methods are valuable for structural assessment, their use in large-scale screening may be limited by equipment availability, operator requirements, and examination cost. Therefore, the portability and low-cost nature of IMU-based methods may provide practical value for preliminary RCI screening in large-scale screening scenarios.

### 5.5 Limitations and future work

This study has a few limitations. First, although this study has included 102 subjects, covering three groups (RCI, adhesive capsulitis, and healthy individuals), the sample size remained limited. The small sample size is a common issue in IMU-based AI models, which is reported with a median of 50 in a recent systematic review study (Smits Serena et al., 2025). Second, this study did not involve clinically commonly used tests such as Neer and Hawkins impingement tests, restricting the model’s generalization in complex clinical settings. Third, this study only preliminarily explored RCI identification in multi motion sequences, and the fusion of multiple actions was performed through feature concatenation. Another limitation is that this study did not compare IMU-based recognition with optoelectronic motion capture system-based recognition. Although IMUs are portable, low-cost, and easy-to-operate, optoelectronic motion capture systems generally provide higher accuracy. Future work should compare these two approaches under the same experimental setting, which can better evaluate the trade-off between measurement accuracy and practical usability. Future research will further investigate more suitable data fusion strategies and establish a more appropriate multi action joint RCI recognition model to address complex multi motion scenarios. Additionally, expanding the sample size to include groups with varies demographic characteristics, such as different age groups, disease stages, onset durations, and integrating clinically diagnostic tests would also enhance the clinical validity and translational potential of models developed in this study.

## 6 Conclusion

This study proposed a RCI recognition model RCIRNet based on IMU data, and systematically assessed recognition performance of RCIRNet and seven traditional ML models from a single motion task to multiple motion task combinations (2–8 motion tasks). Results show that selecting specific movements is more critical than increasing the number of movements for effective RCI recognition. The proposed RCIRNet performed best compared with ML models in all tasks, and the combination of action shoulder frontal ROM test and RCIRNet is the best configuration for RCI recognition. Future research will expand sample size and explore suitable data fusion strategies.

## Data Availability

Due to strict ethical restrictions on human subject data, the generated datasets are not available upon request. Requests to access the datasets should be directed to Yunru Ma, y.ma@fjmu.edu.cn.
