# Supplementary material for: IMU-based rotator cuff injury recognition with varying configurations and combinations
Source: Front Physiol. 2026 Jul 6;17:1794238. doi: 10.3389/fphys.2026.1794238 (PMC13381275; doi:10.3389/fphys.2026.1794238)
Supplement: Supplementary file 1 [file DataSheet1.docx]

Supplementary Material

For RCI identification based on IMU motion data, the recognition performance of eight distinct movements was investigated: axilla wash, perineal care, hand to mouth, combing hair, forward and upward reach with 1kg item, and shoulder sagittal and frontal range of motion test. Experiments were conducted across eight models (based on machine learning and deep learning), with results presented in Figures 1-5. Overall, the classification Accuracy, F1-score, and AUC ranged from 0.50 to 0.89, 0.46 to 0.89, and 0.50 to 0.93, respectively.


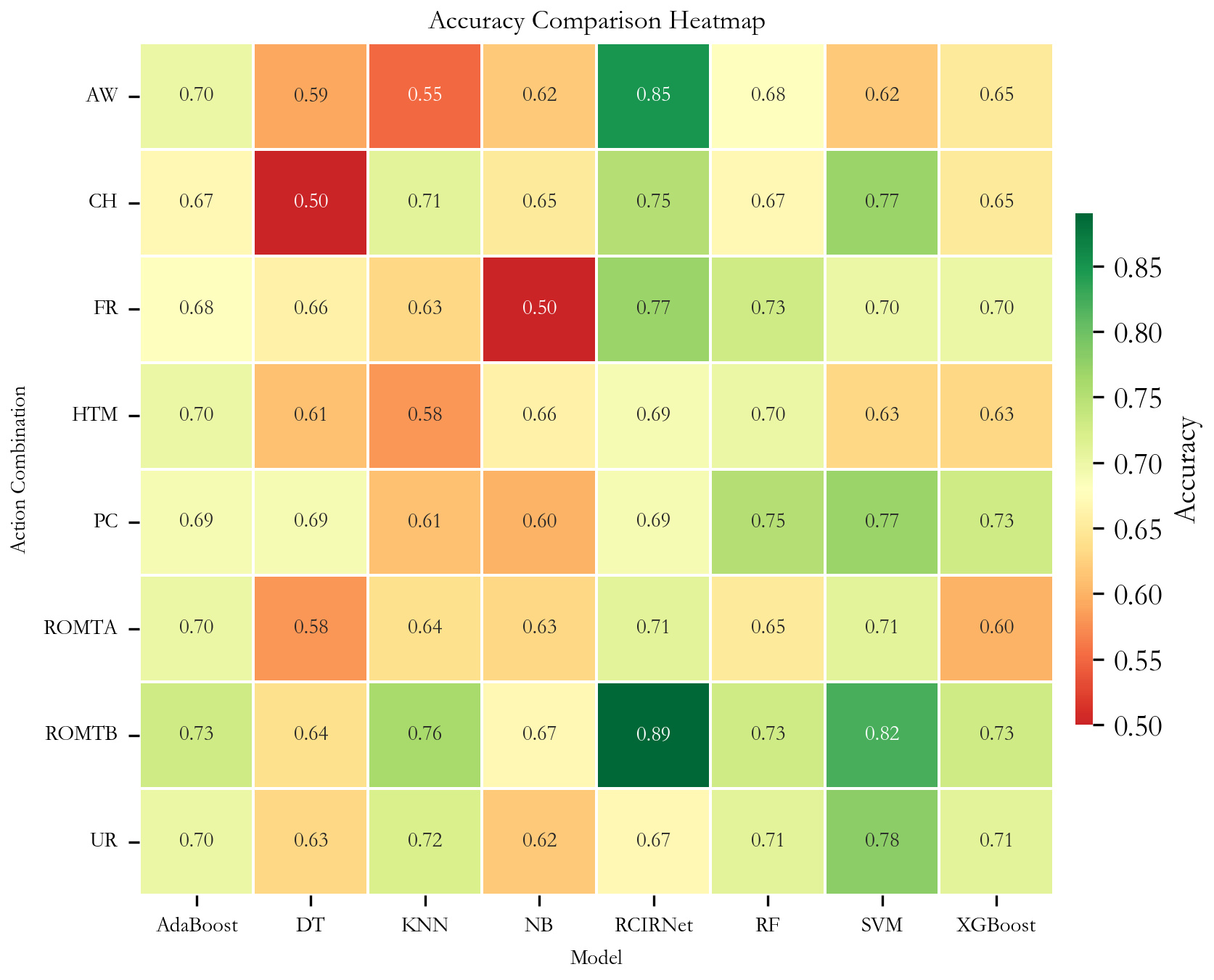


**Supplementary Figure 1.** Accuracy of signal action combination experiments.


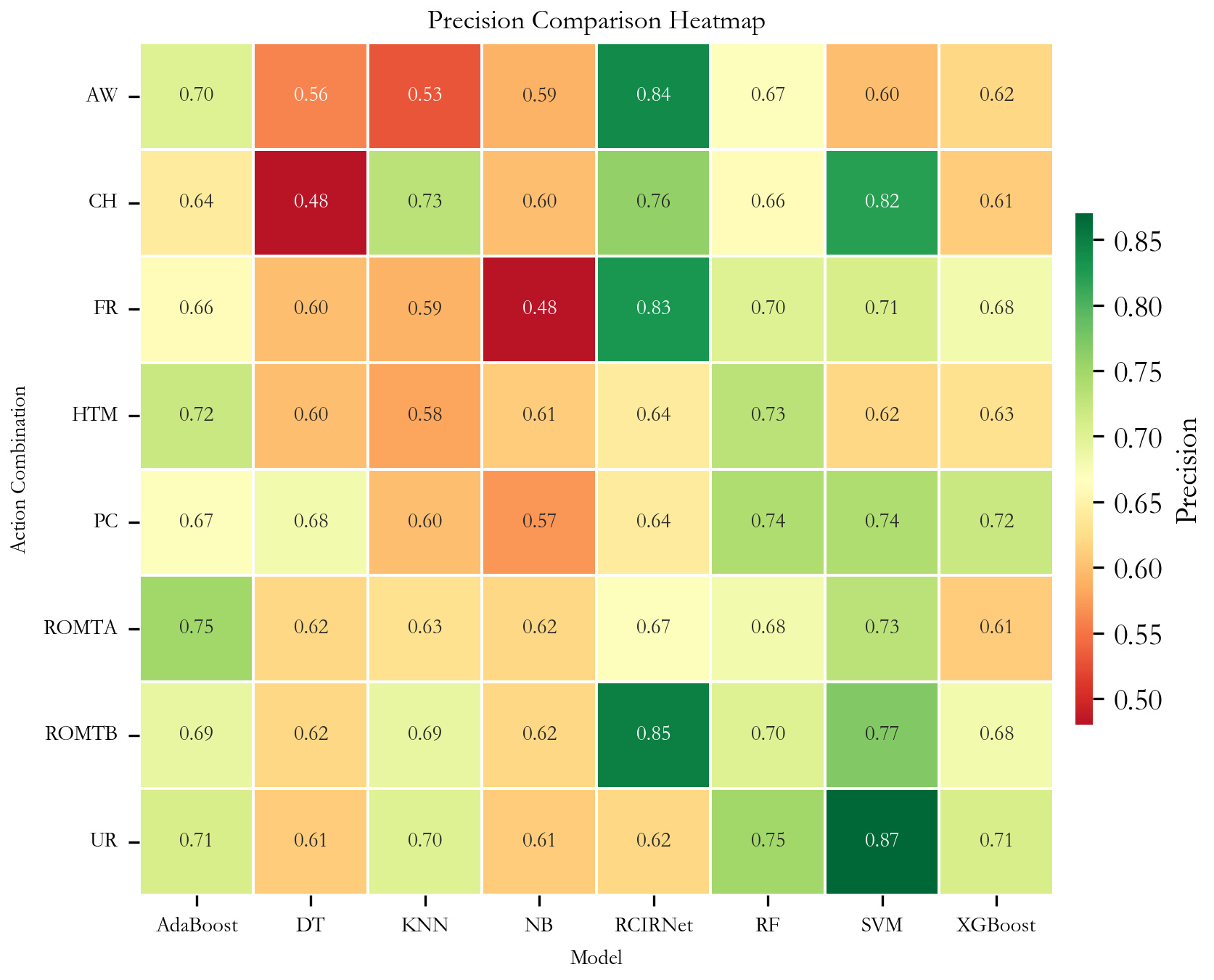


**Supplementary Figure 2.** Precision of signal action combination experiments.


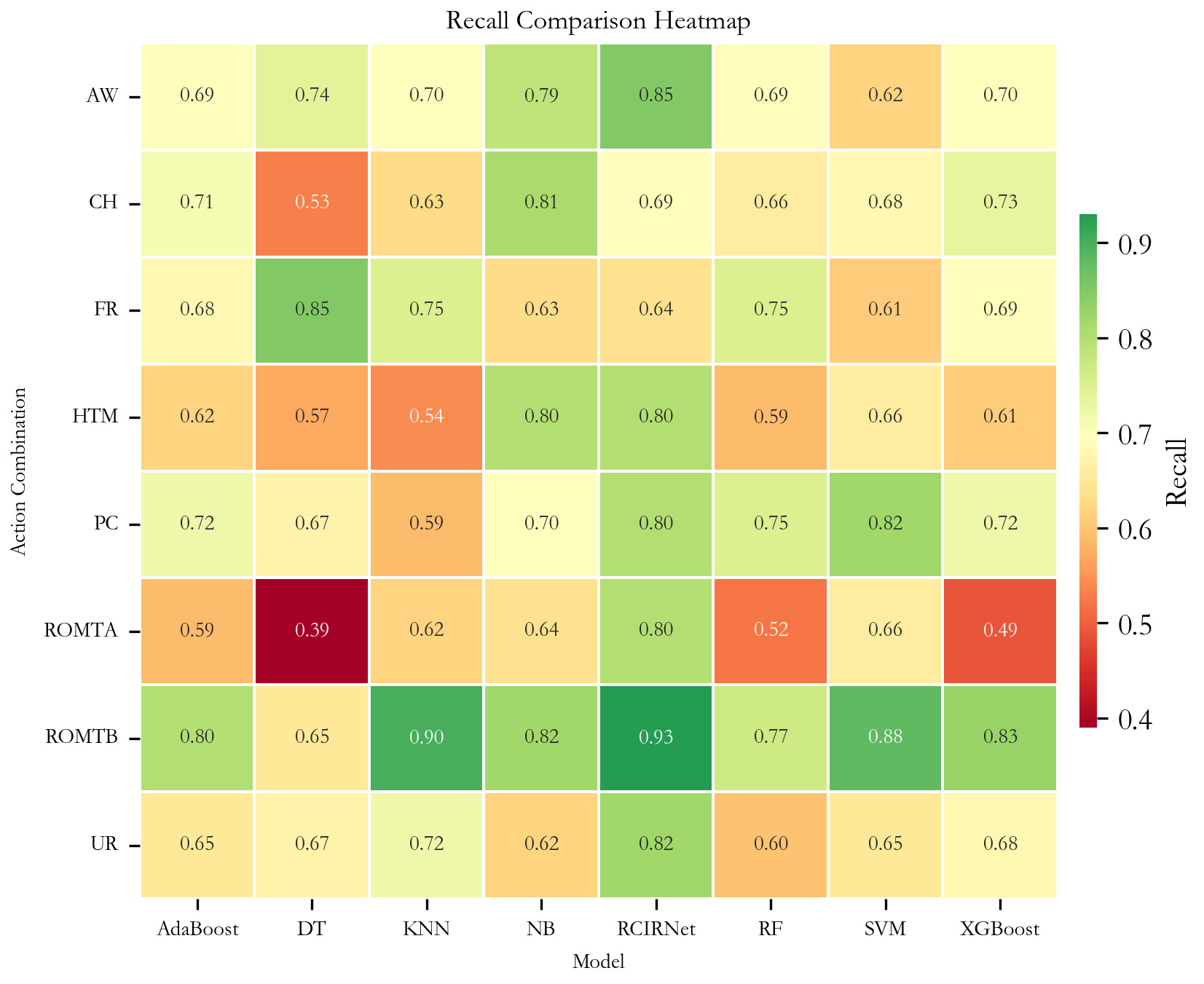


**Supplementary Figure 3.** Recall of signal action combination experiments.


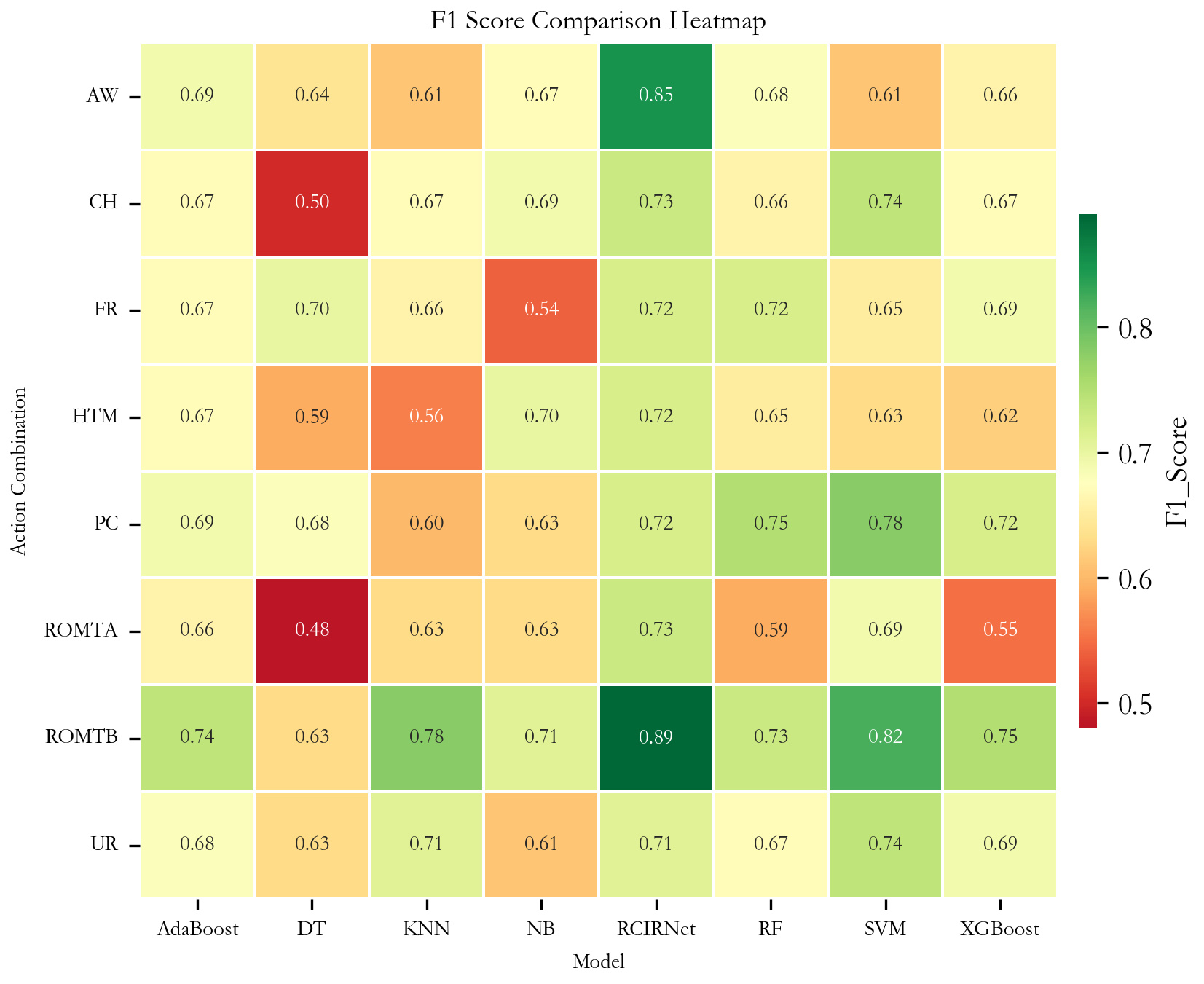


**Supplementary Figure 4.** F1-score of signal action combination experiments.


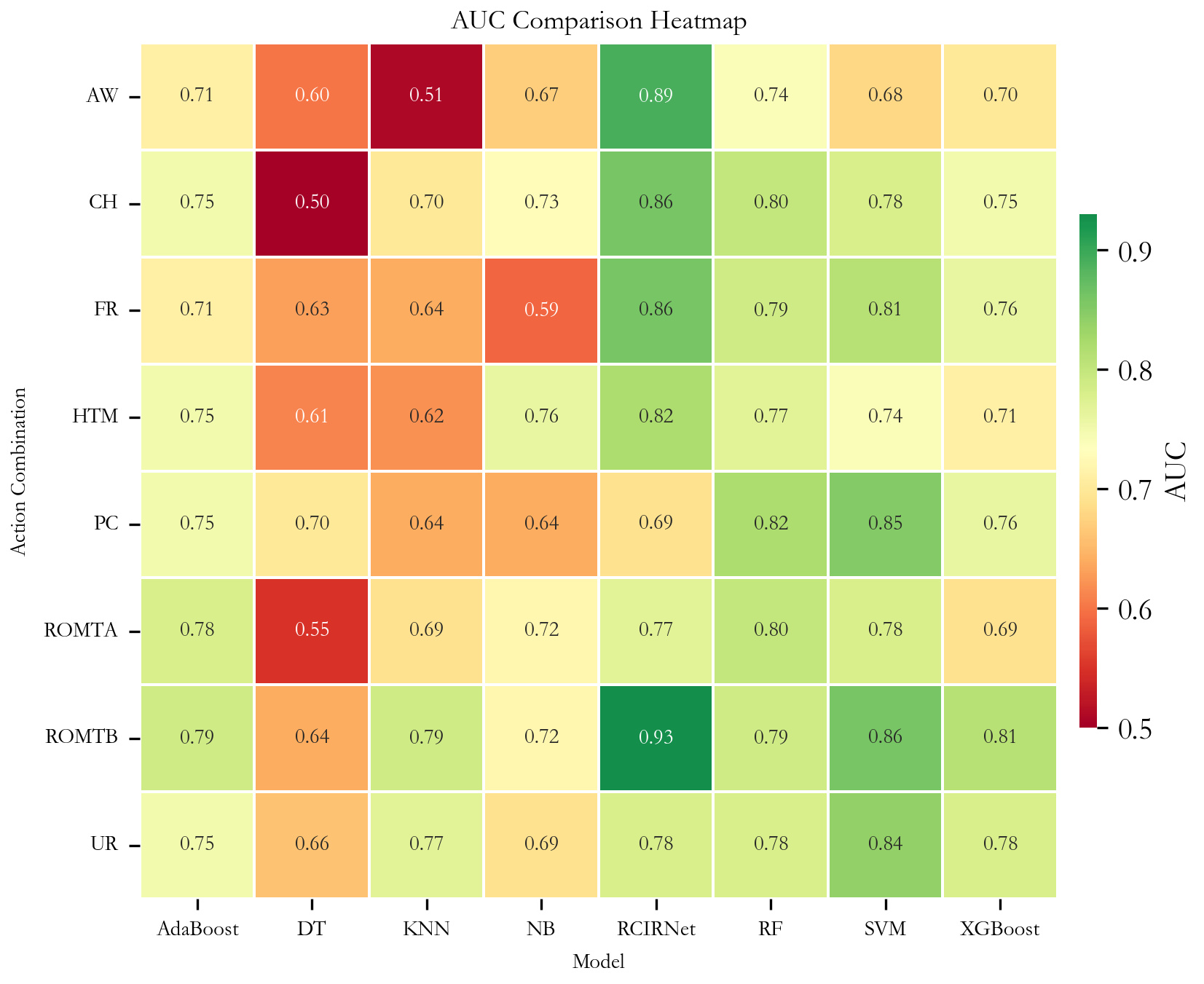


**Supplementary Figure 5.** AUC of signal action combination experiments.

We also investigated the identification performance for multi-action sequences, including two-action sequences (28 combinations), three-action sequences (56 combinations), four-action sequences (70 combinations), five-action sequences (56 combinations), six-action sequences (28 combinations), seven-action sequences (8 combinations), and eight-action sequences (1 combination), totally obtaining 247 combinations. Figures 6-10 present experimental results for RCI recognition through dual-action combinations. The classification Accuracy, F1-score, and AUC ranged from 0.42 to 0.83, 0.33 to 0.83, and 0.32 to 0.90, respectively.

**
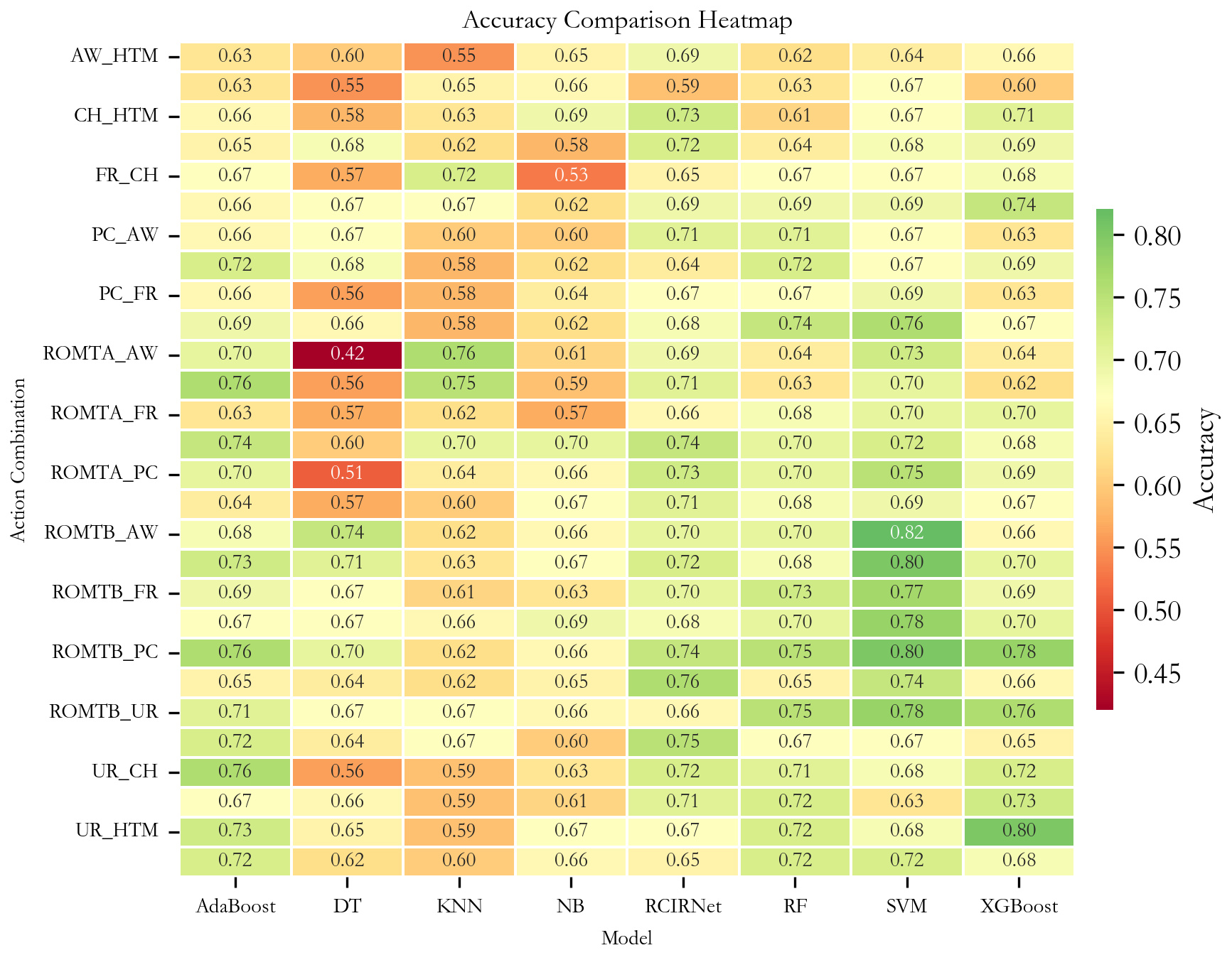
**

**Supplementary Figure 6.** Accuracy of dual-action combination experiments.


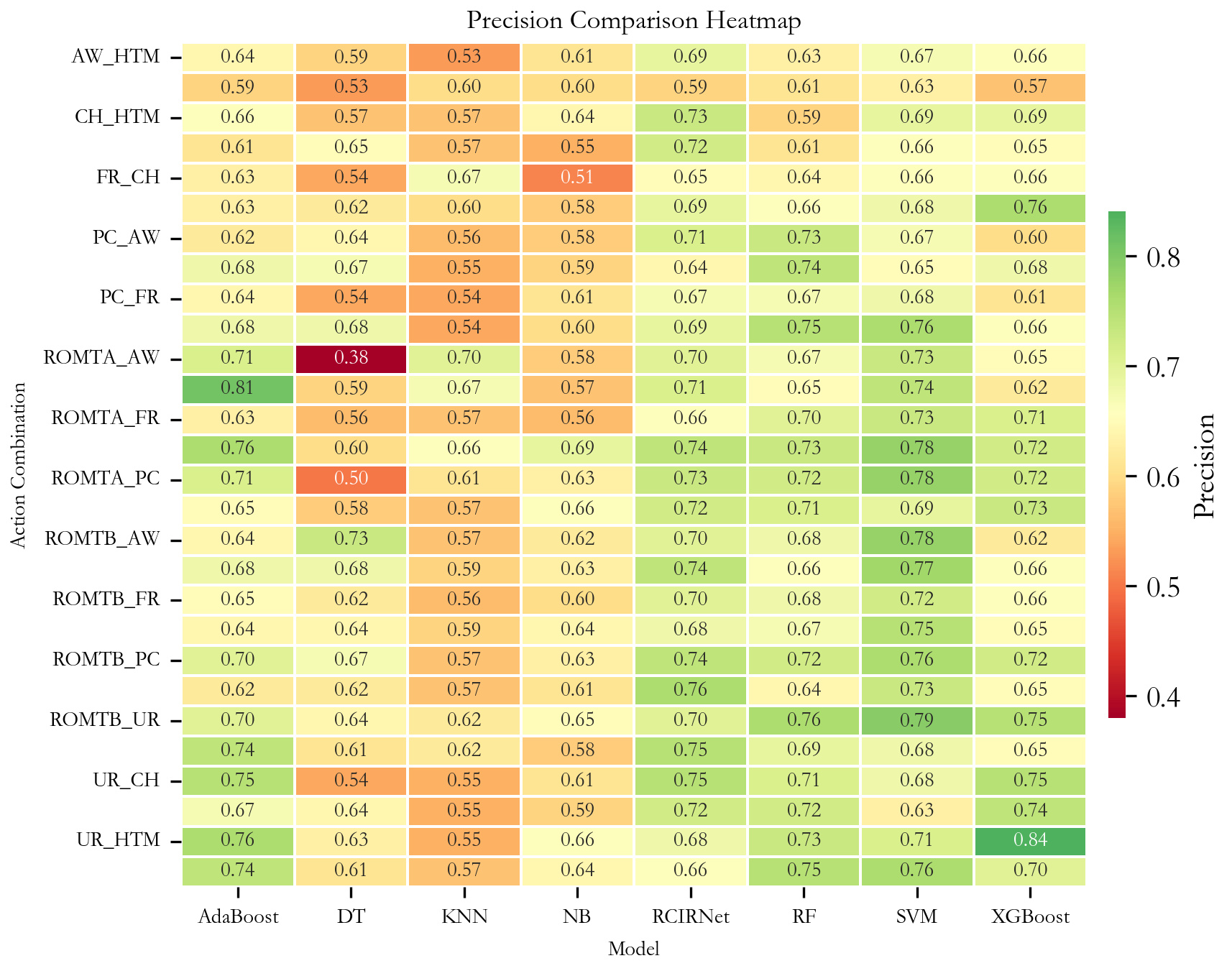


**Supplementary Figure 7.** Precision of dual-action combination experiments.


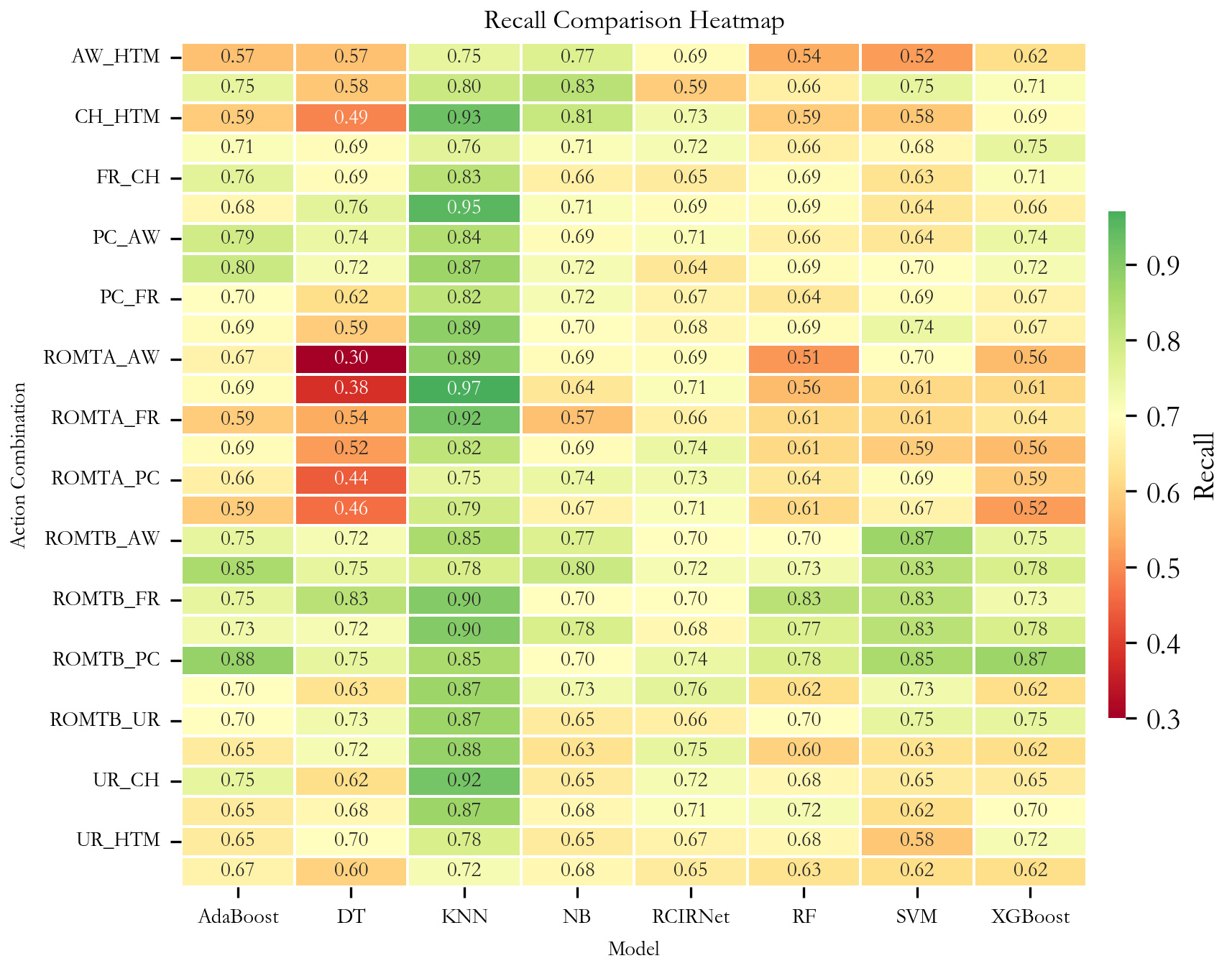


**Supplementary Figure 8.** Recall of dual-action combination experiments.


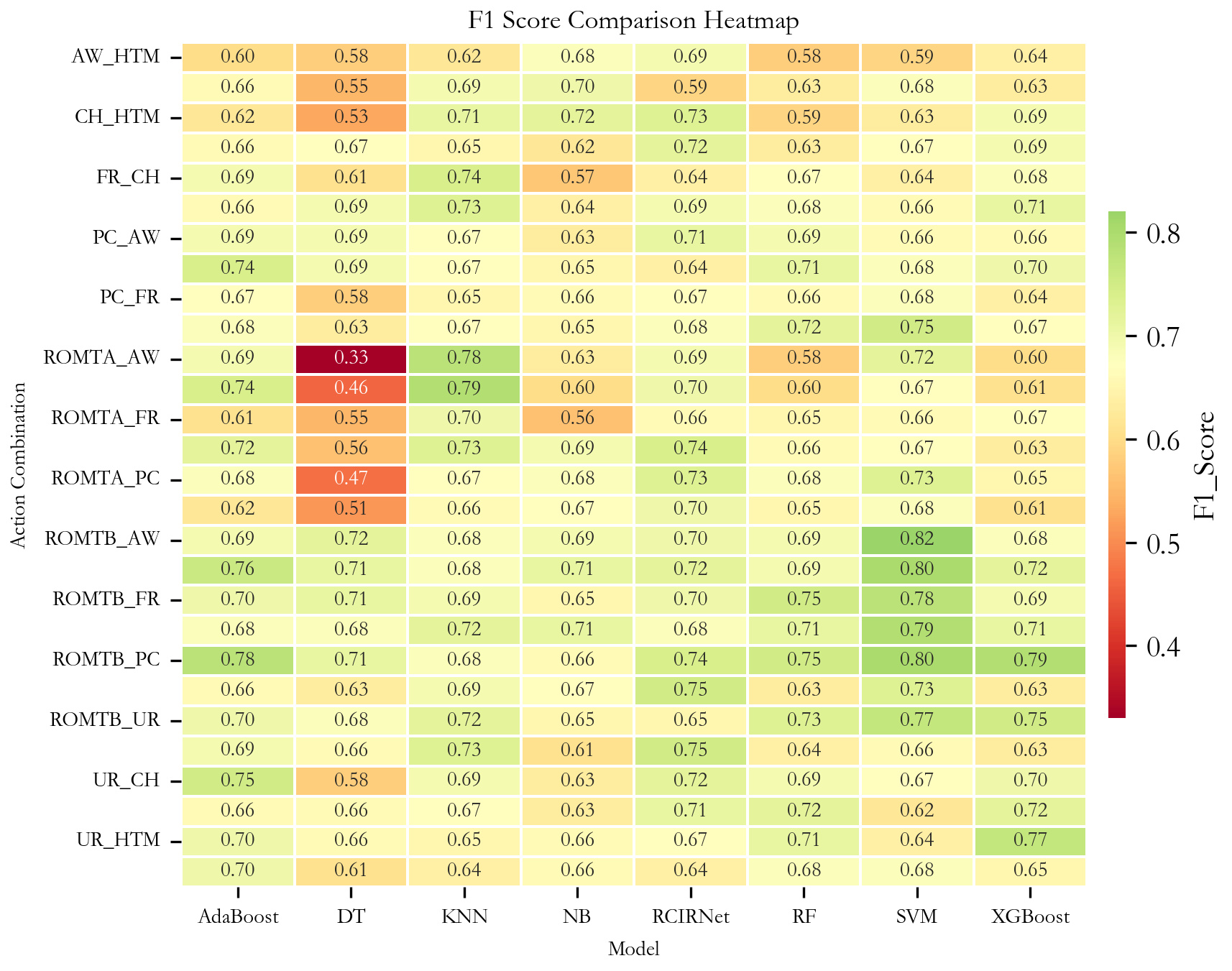


**Supplementary Figure 9.** F1-score of dual-action combination experiments.


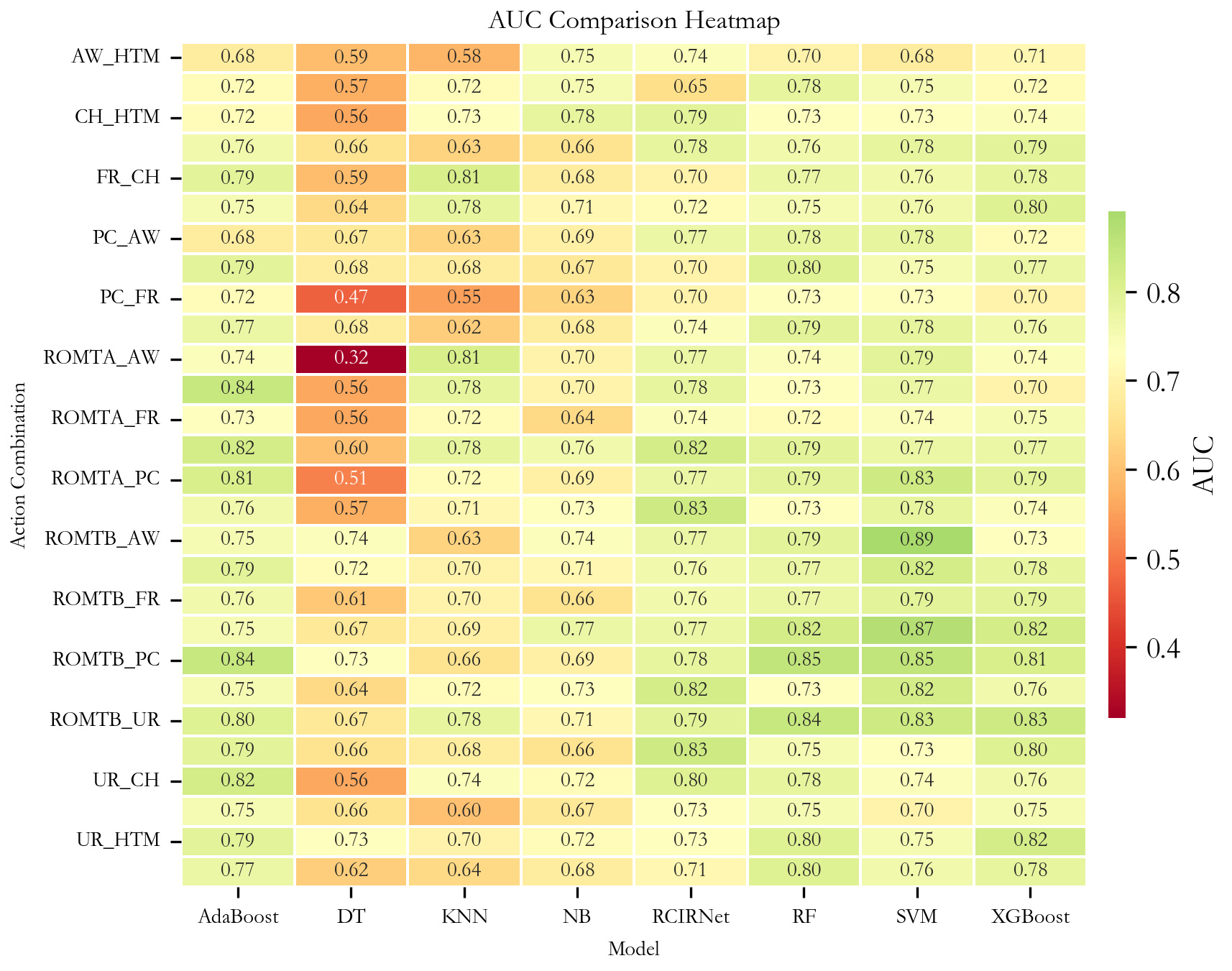


**Supplementary Figure 10.** AUC of dual-action combination experiments.

Figures 11-15 present experimental results for RCI identification through three combined movements. The recognition Accuracy, F1-score, and AUC ranged from 0.46 to 0.86, 0.44 to 0.86, and 0.40 to 0.92, respectively.


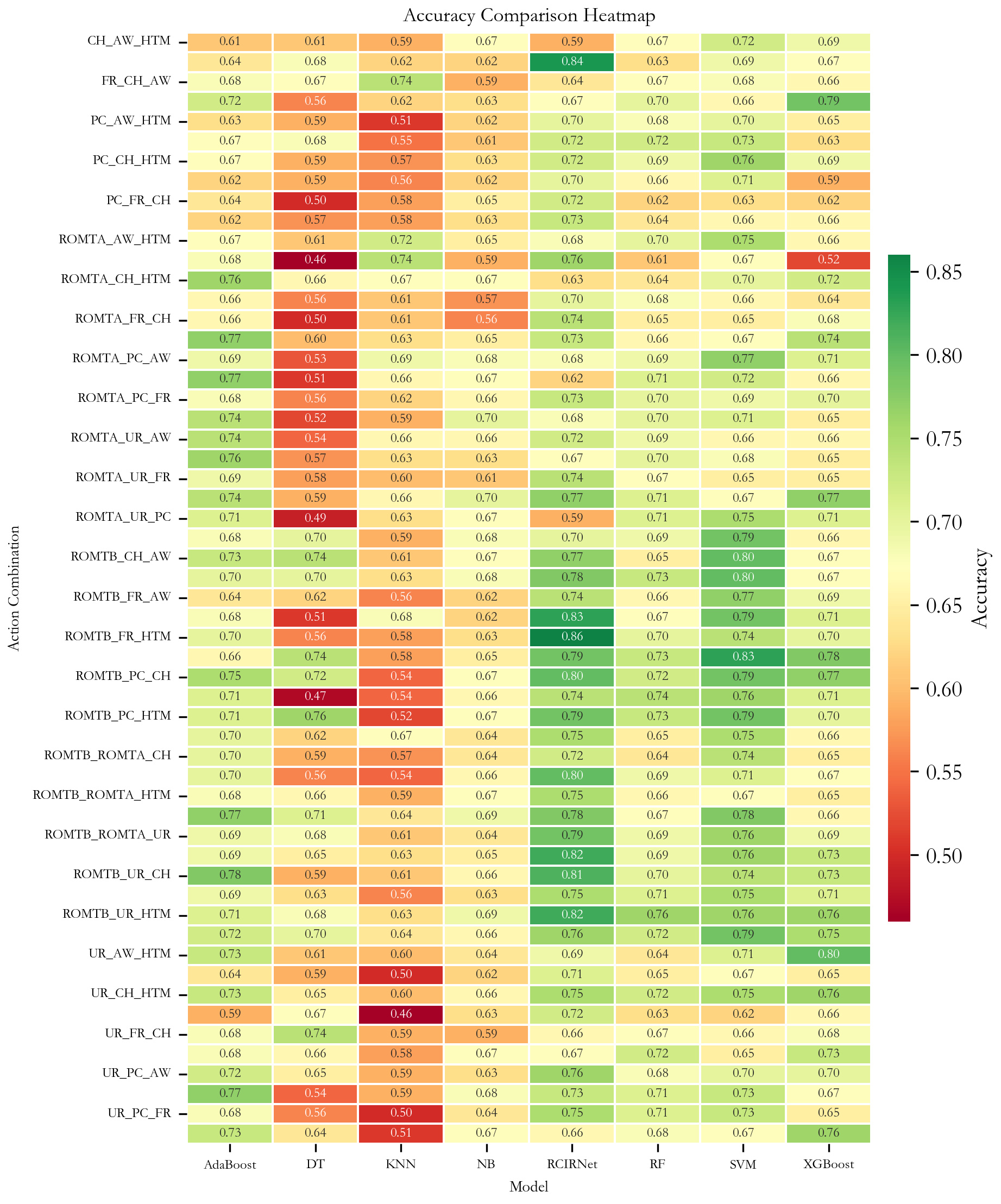


**Supplementary Figure 11.** Accuracy of three-action combination experiments.


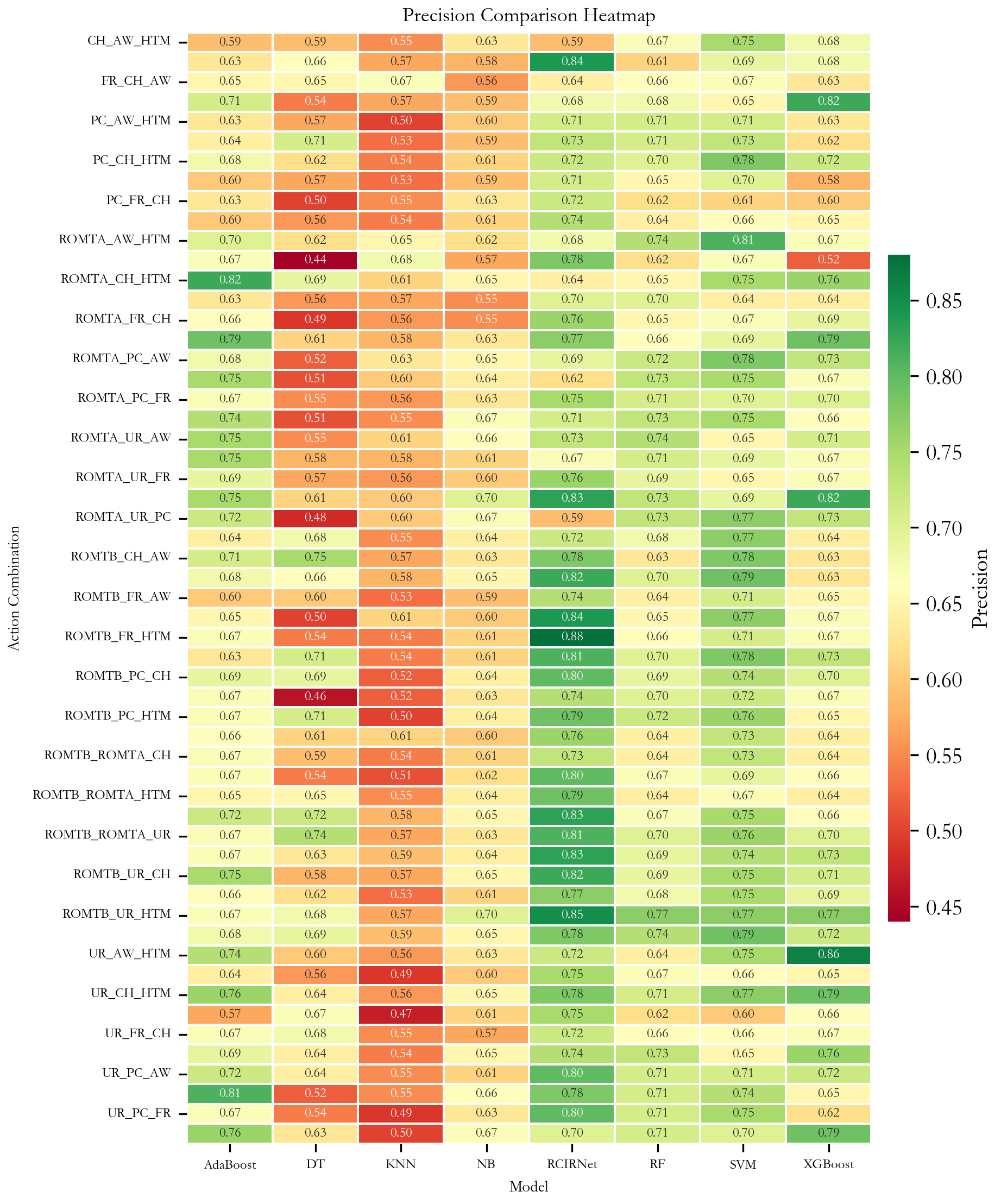


**Supplementary Figure 12.** Precision of three-action combination experiments.


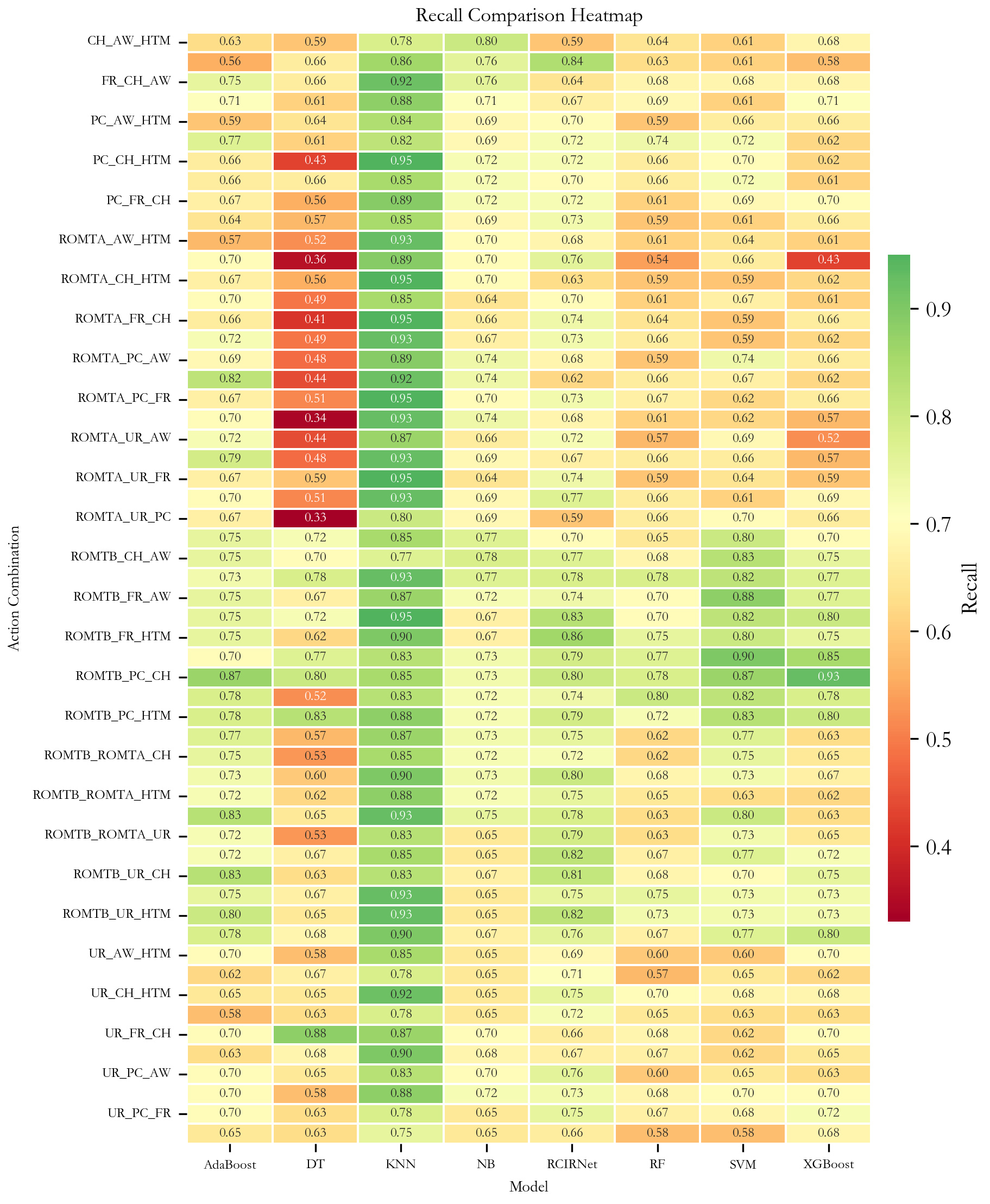


**Supplementary Figure 13.** Recall of three-action combination experiments.


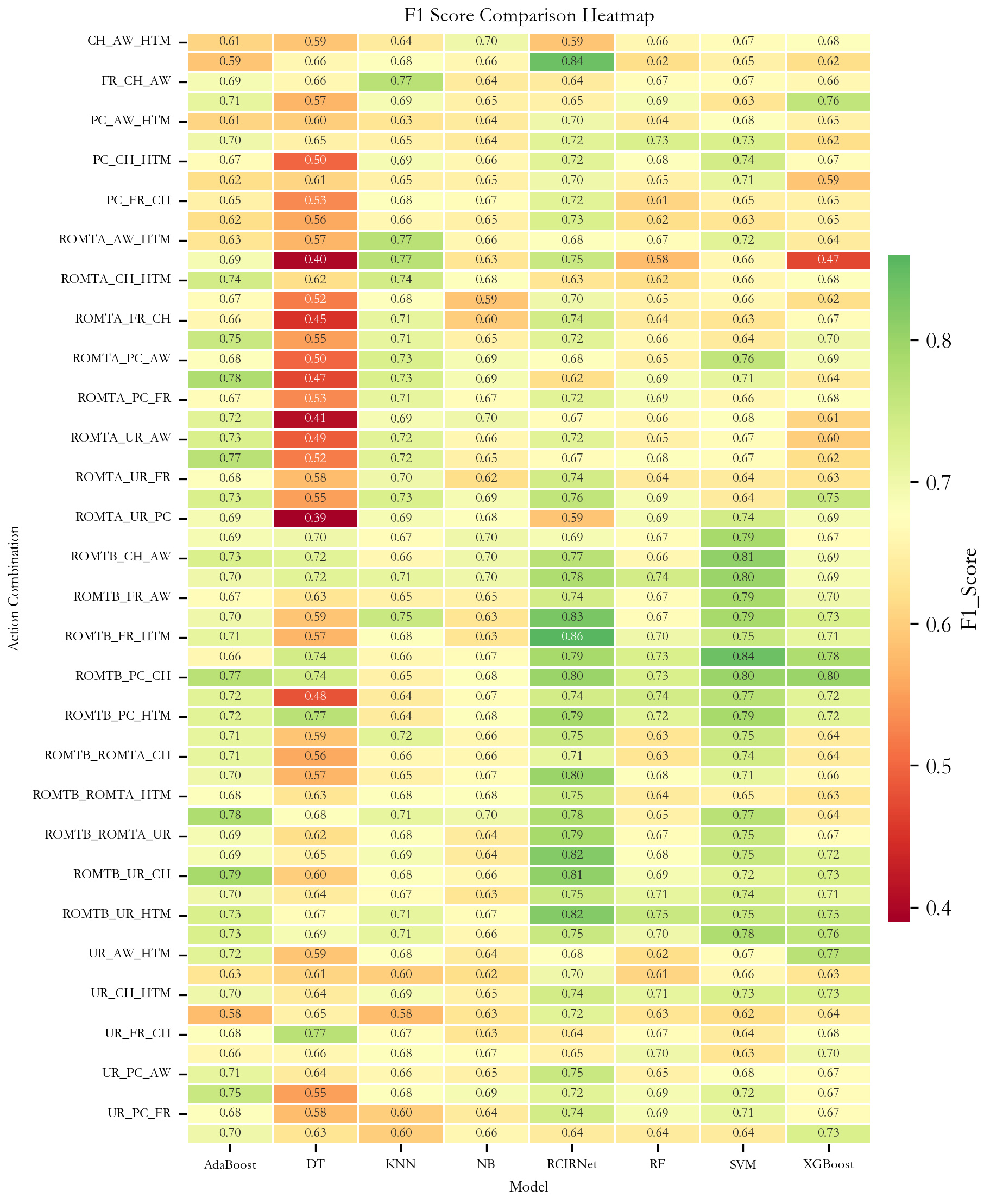


**Supplementary Figure 14.** F1-score of three-action combination experiments.


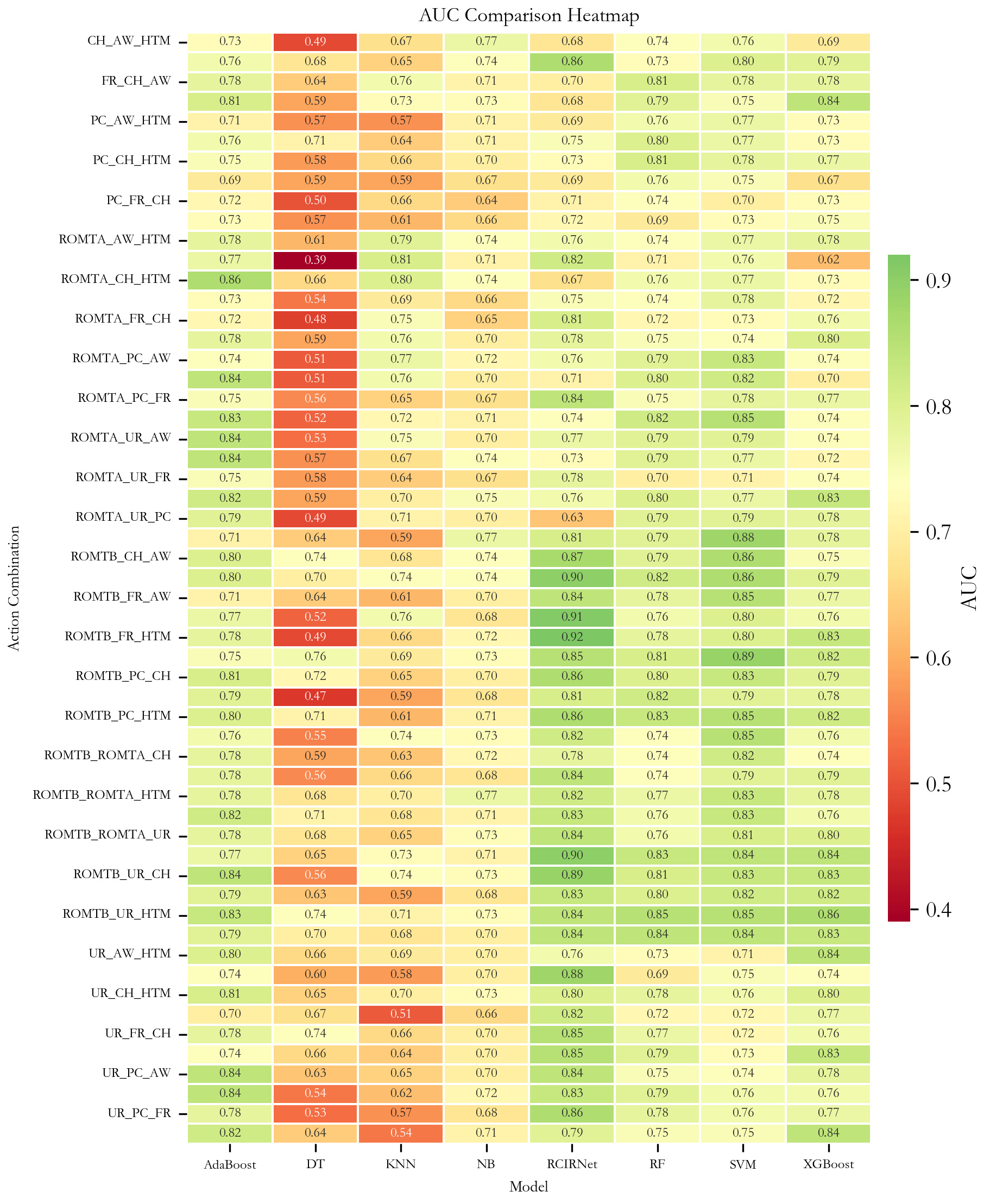


**Supplementary Figure 15.** AUC of three-action combination experiments.

Figures 16-20 present classification results for RCI recognition across four combined movements. The findings indicate that recognition Accuracy, F1-score, and AUC ranged from 0.47 to 0.88, 0.38 to 0.88, and 0.49 to 0.94, respectively.


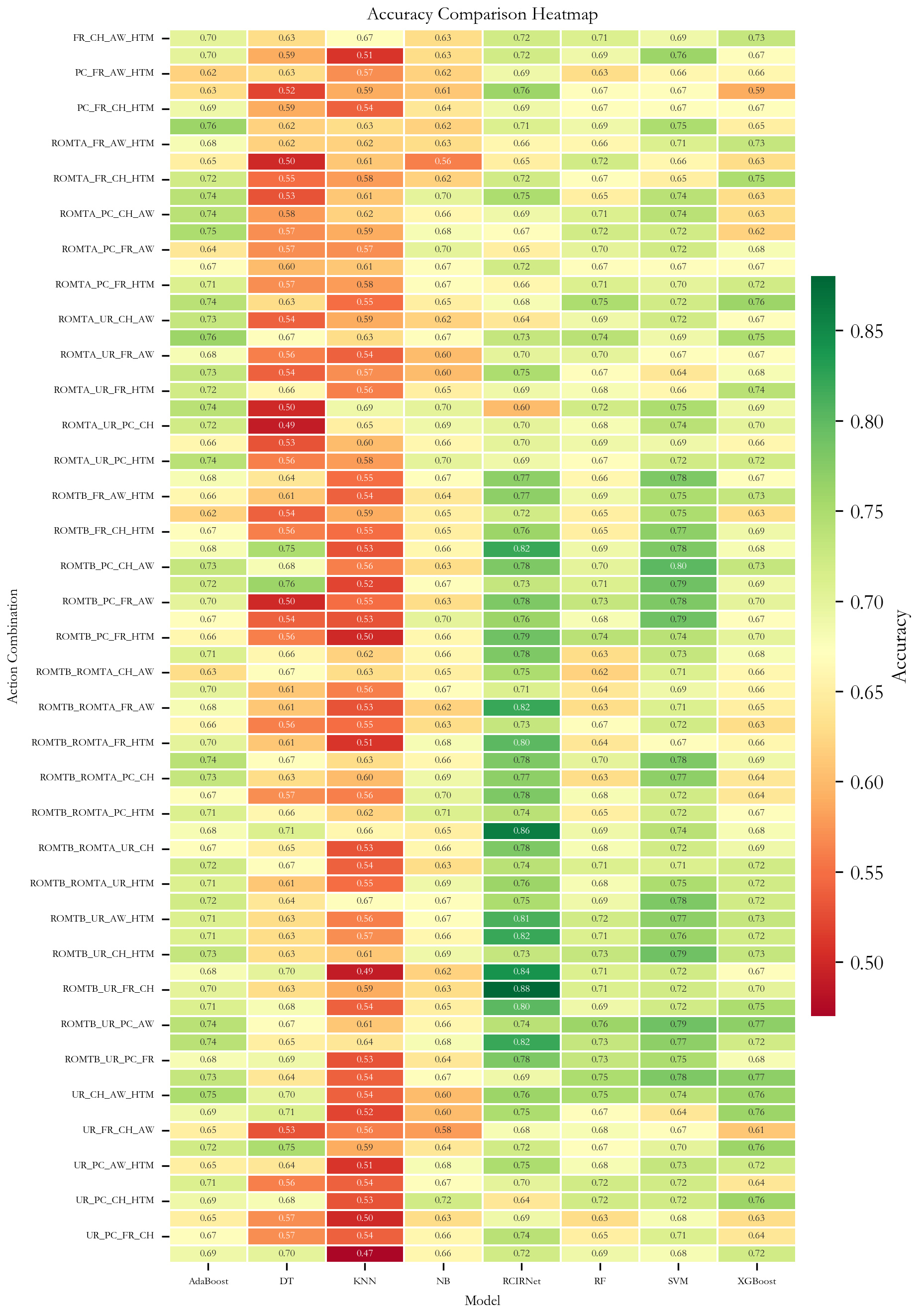


**Supplementary Figure 16.** Accuracy of four-action combination experiments.


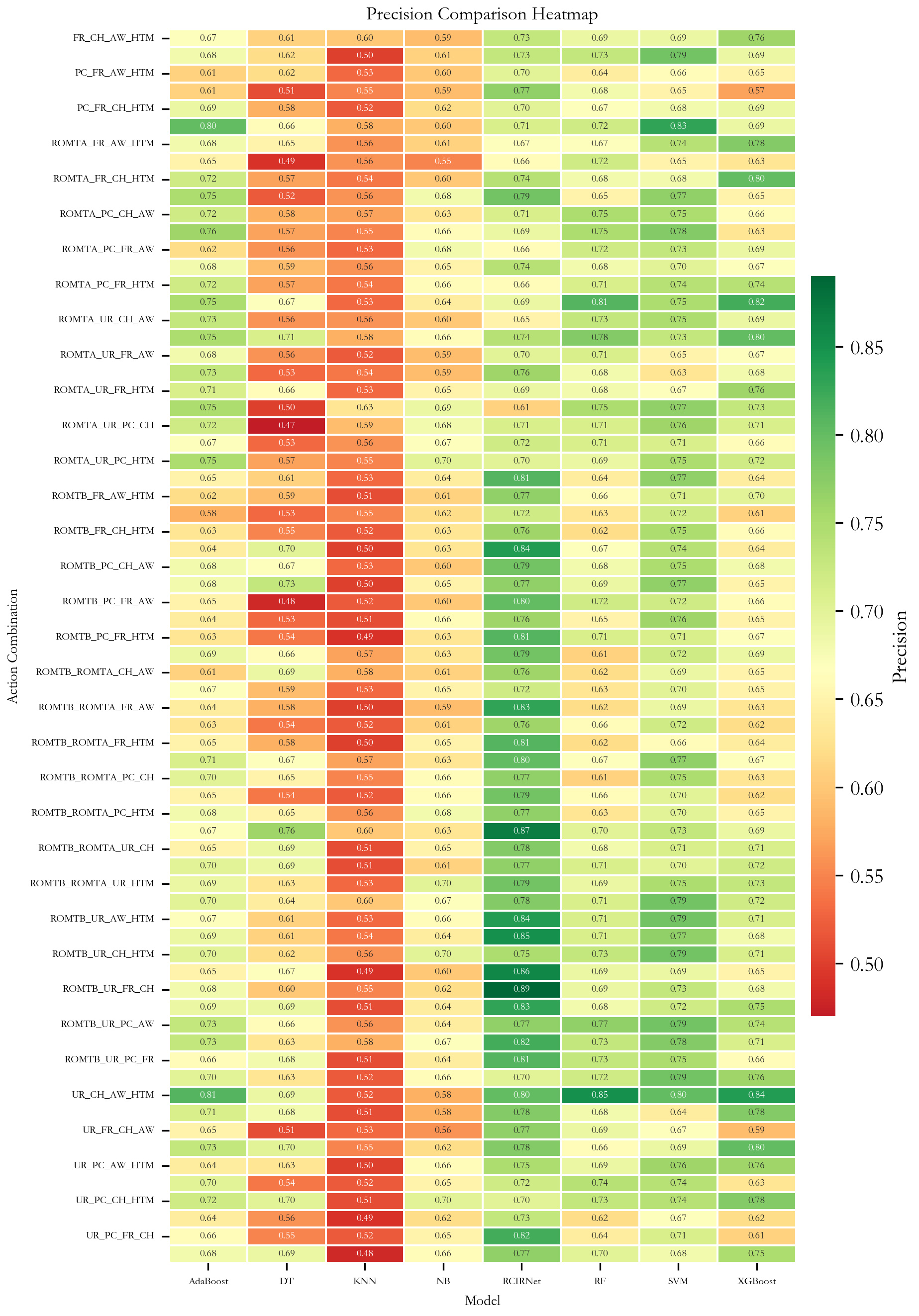


**Supplementary Figure 17.** Precision of four-action combination experiments.


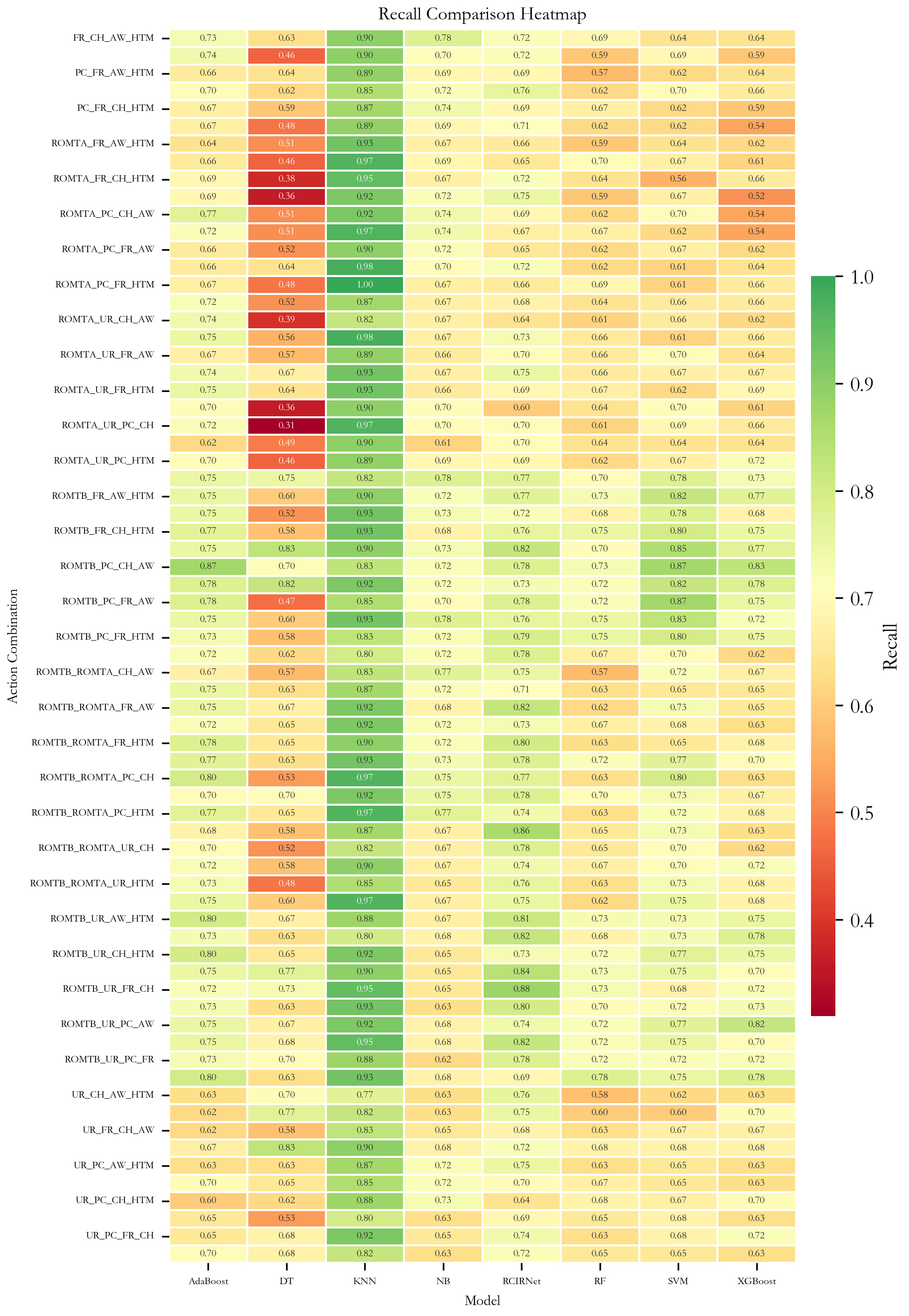


**Supplementary Figure 18.** Recall of four-action combination experiments.


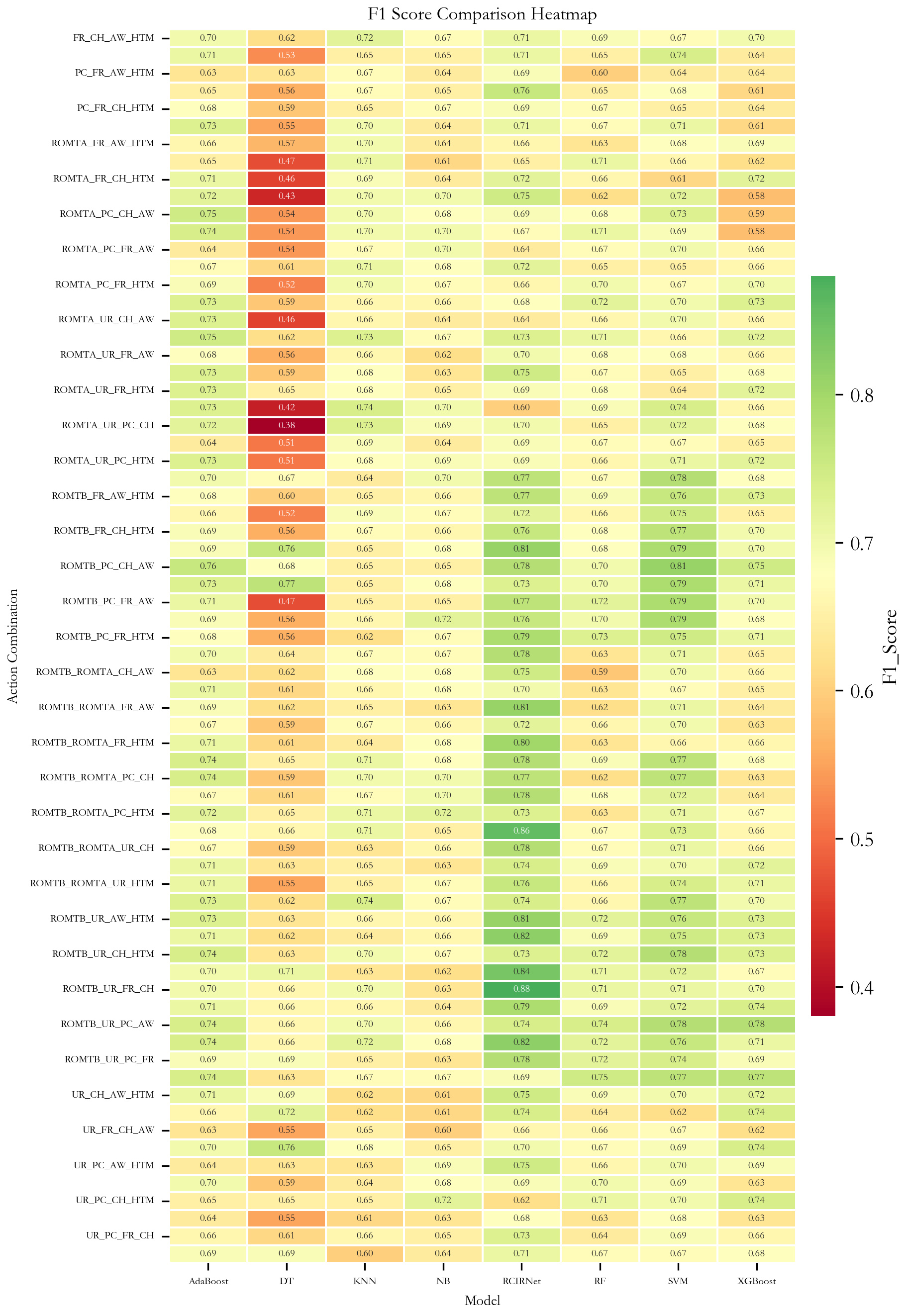


**Supplementary Figure 19.** F1-score of four-action combination experiments.


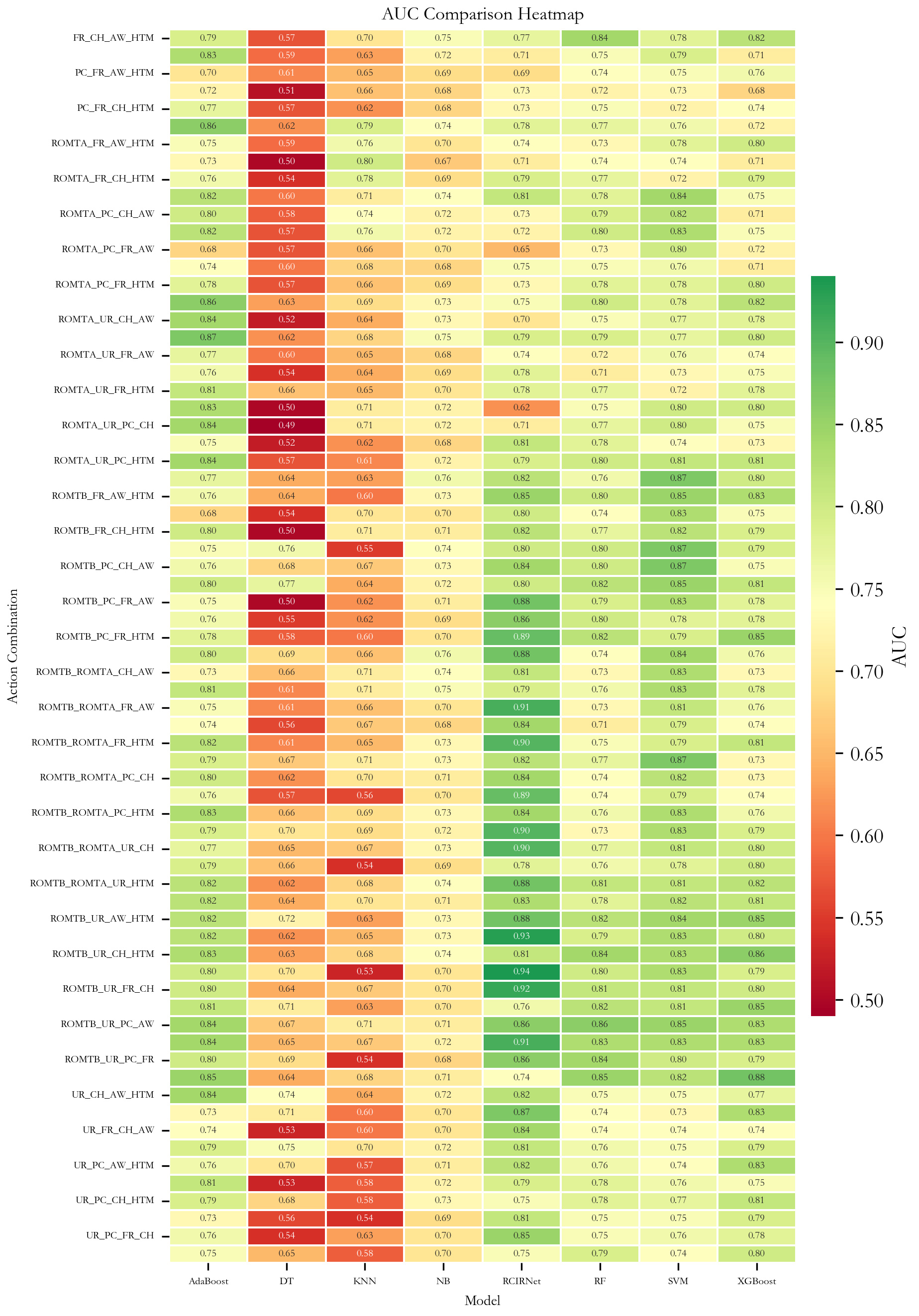


**Supplementary Figure 20.** AUC of four-action combination experiments.

Figures 21-25 present experimental results for RCI recognition through five combined movements. Overall, the identification Accuracy, F1-score, and AUC ranged from 0.46 to 0.85, 0.85 to 0.80, and 0.46 to 0.92, respectively.


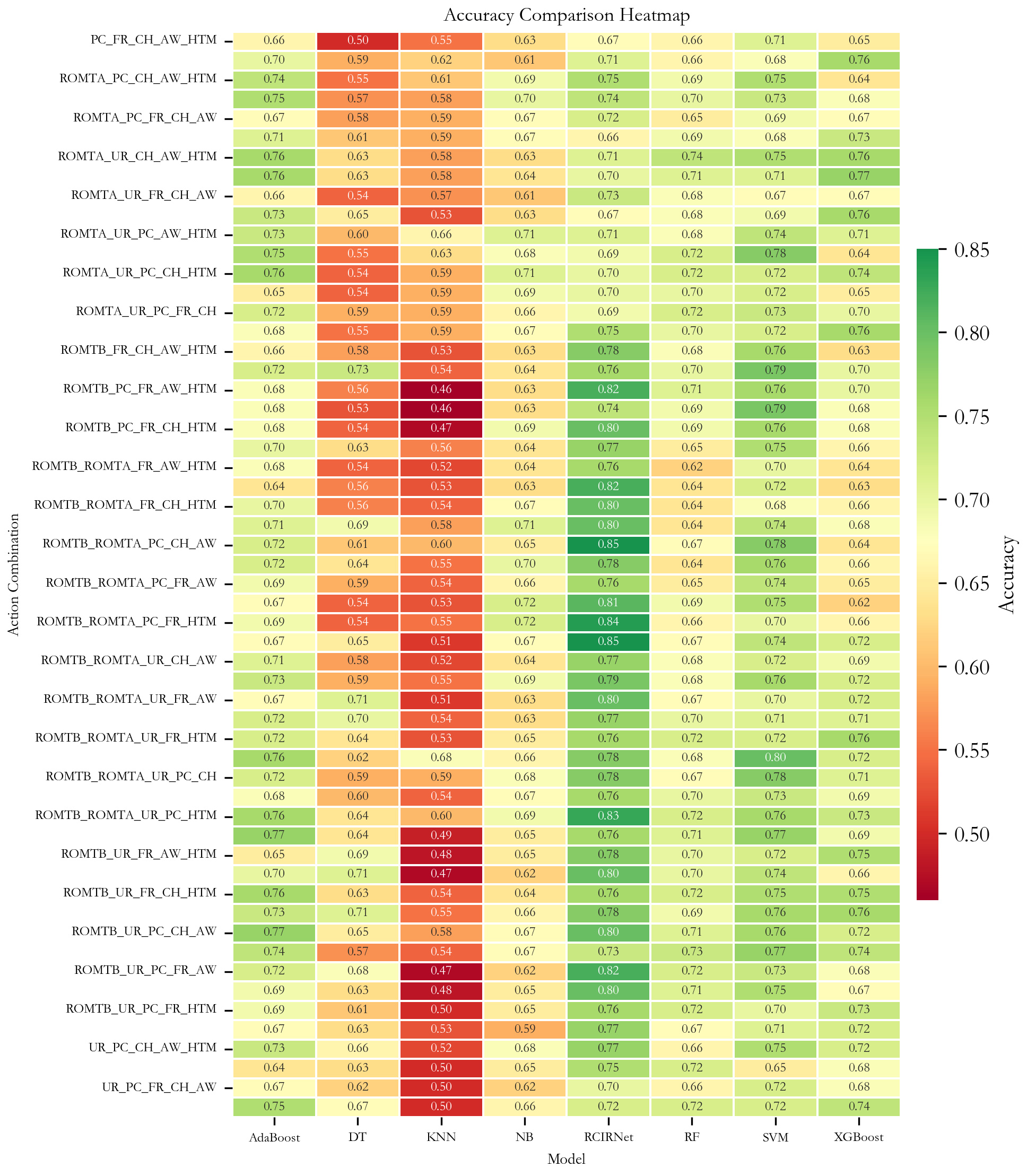


**Supplementary Figure 21.** Accuracy of five-action combination experiments.


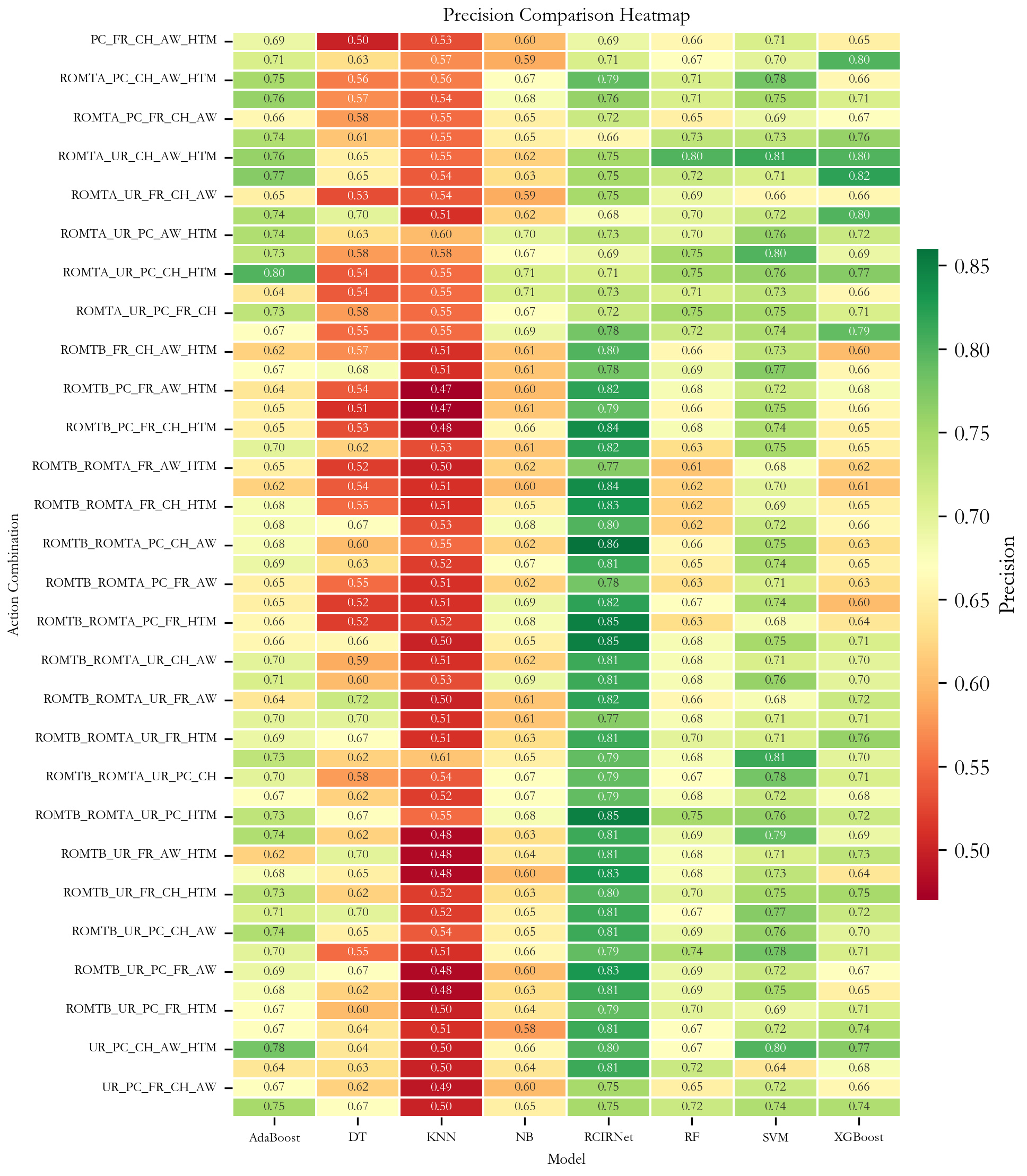


**Supplementary Figure 22.** Precision of five-action combination experiments.


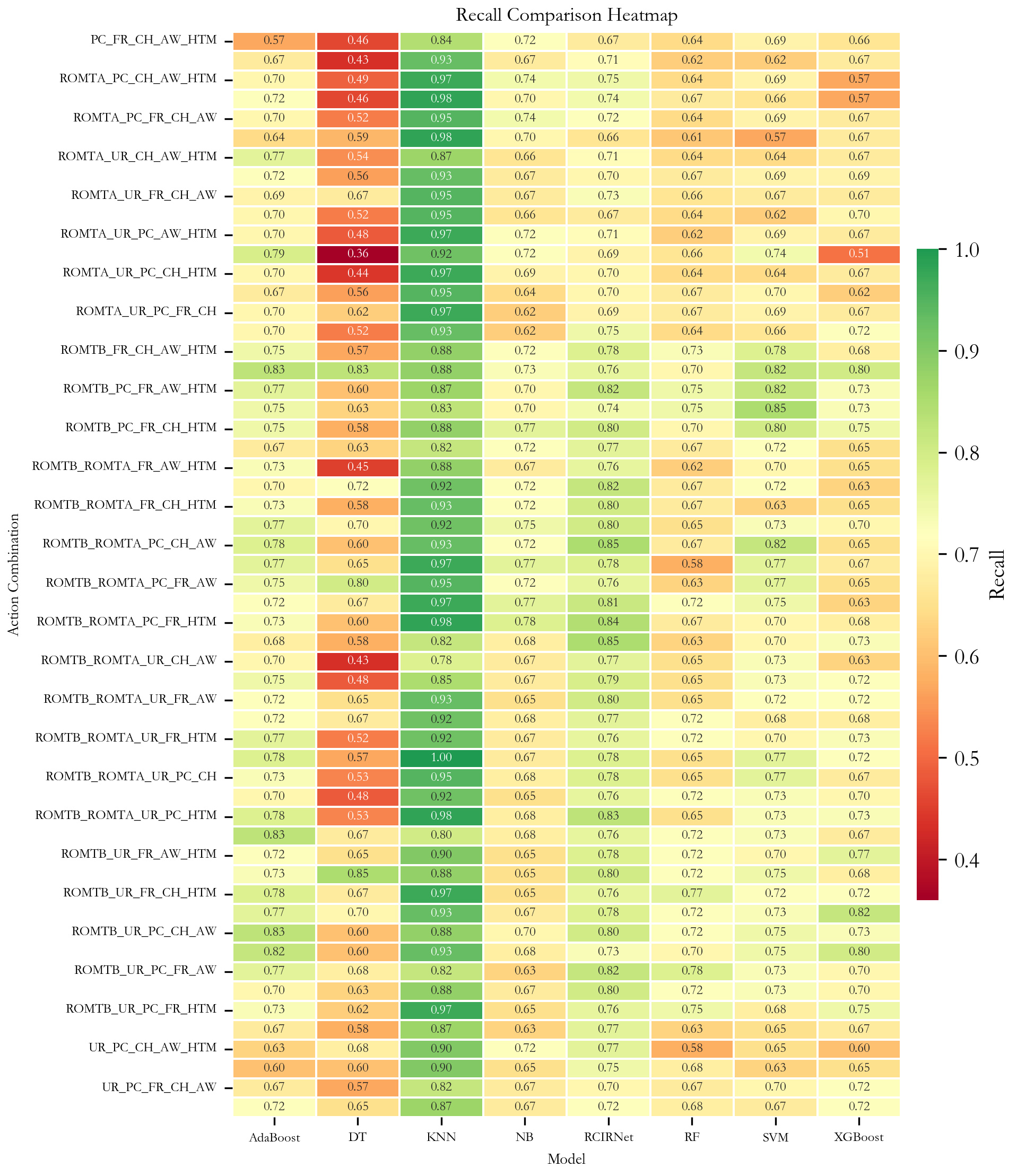


**Supplementary Figure 23.** Recall of five-action combination experiments.


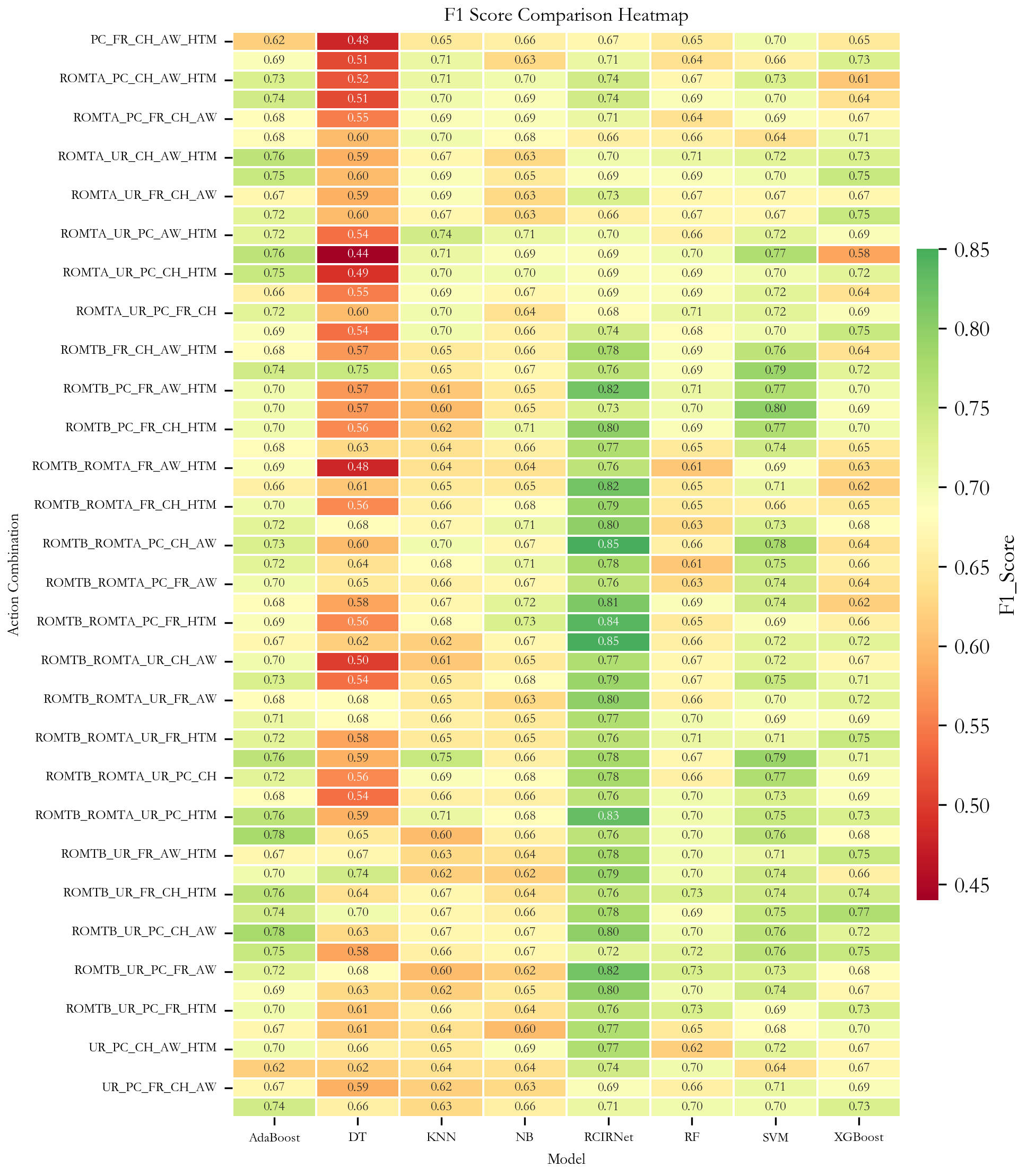


**Supplementary Figure 24.** F1-score of five-action combination experiments.


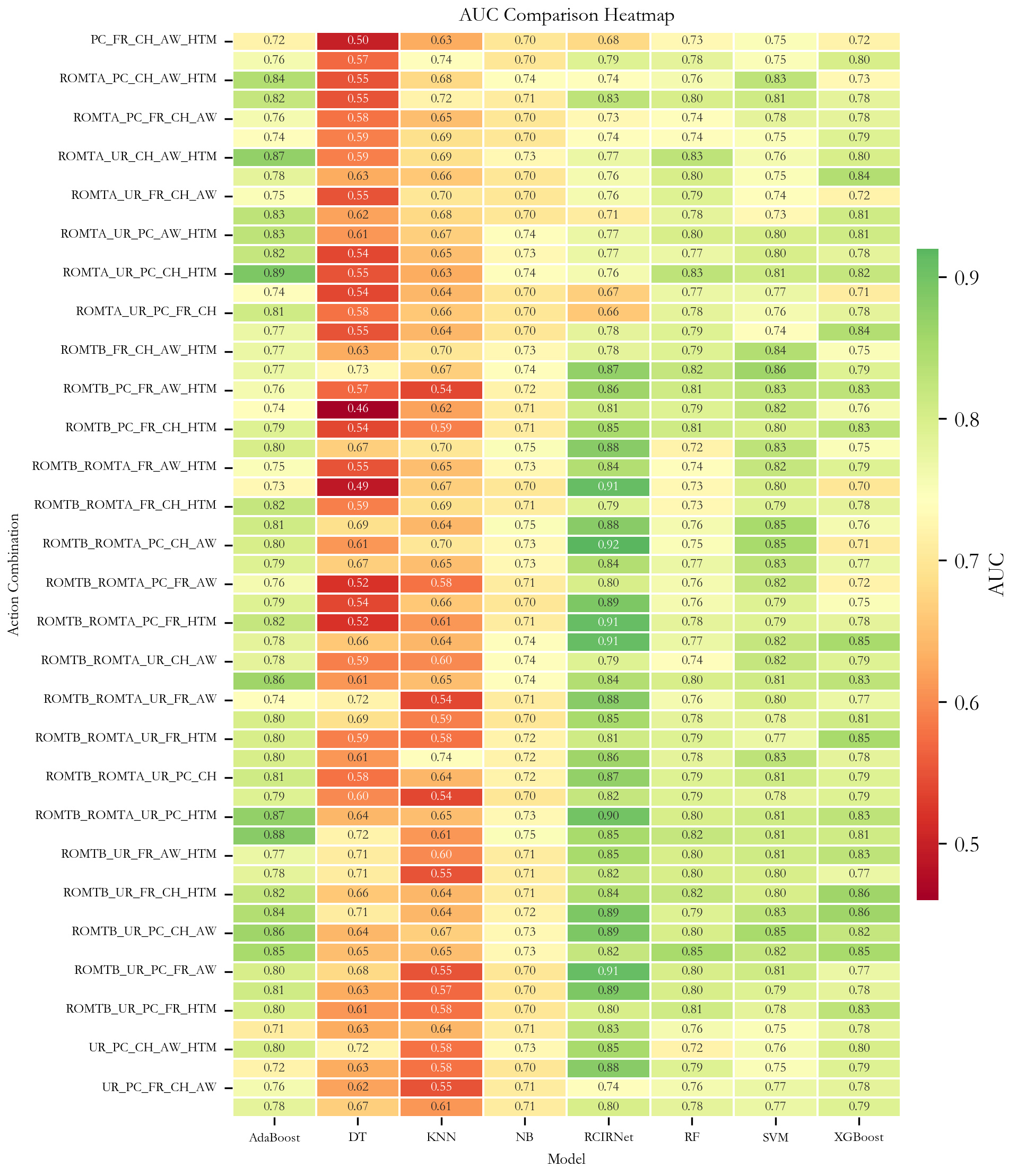


**Supplementary Figure 25.** AUC of five-action combination experiments.

Figures 26-30 present experimental results for RCI identification through six combined movements. The classification Accuracy, F1-score, and AUC ranged from 0.45 to 0.82, 0.52 to 0.82, and 0.44 to 0.90, respectively.


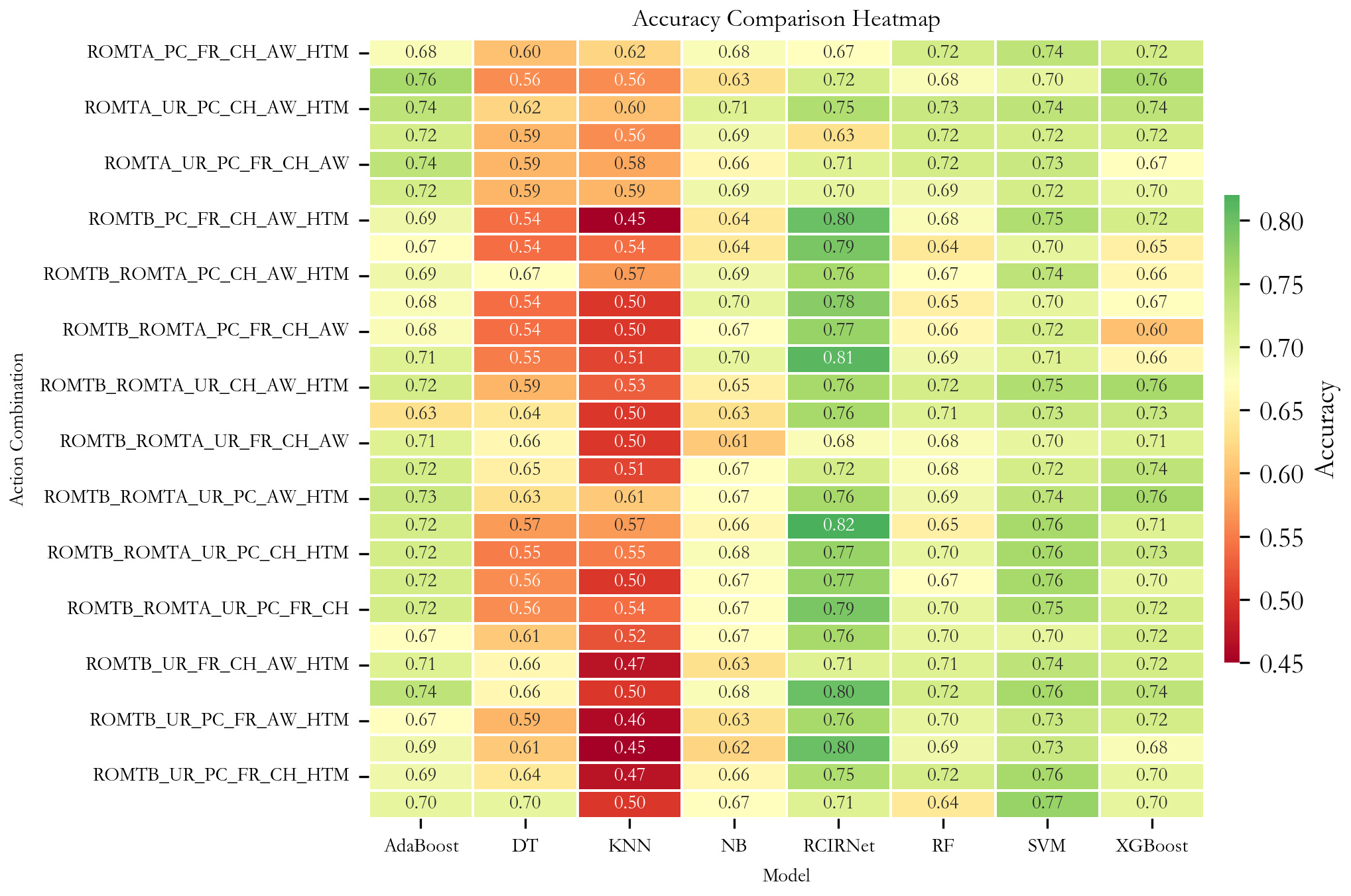


**Supplementary Figure 26.** Accuracy of six-action combination experiments.


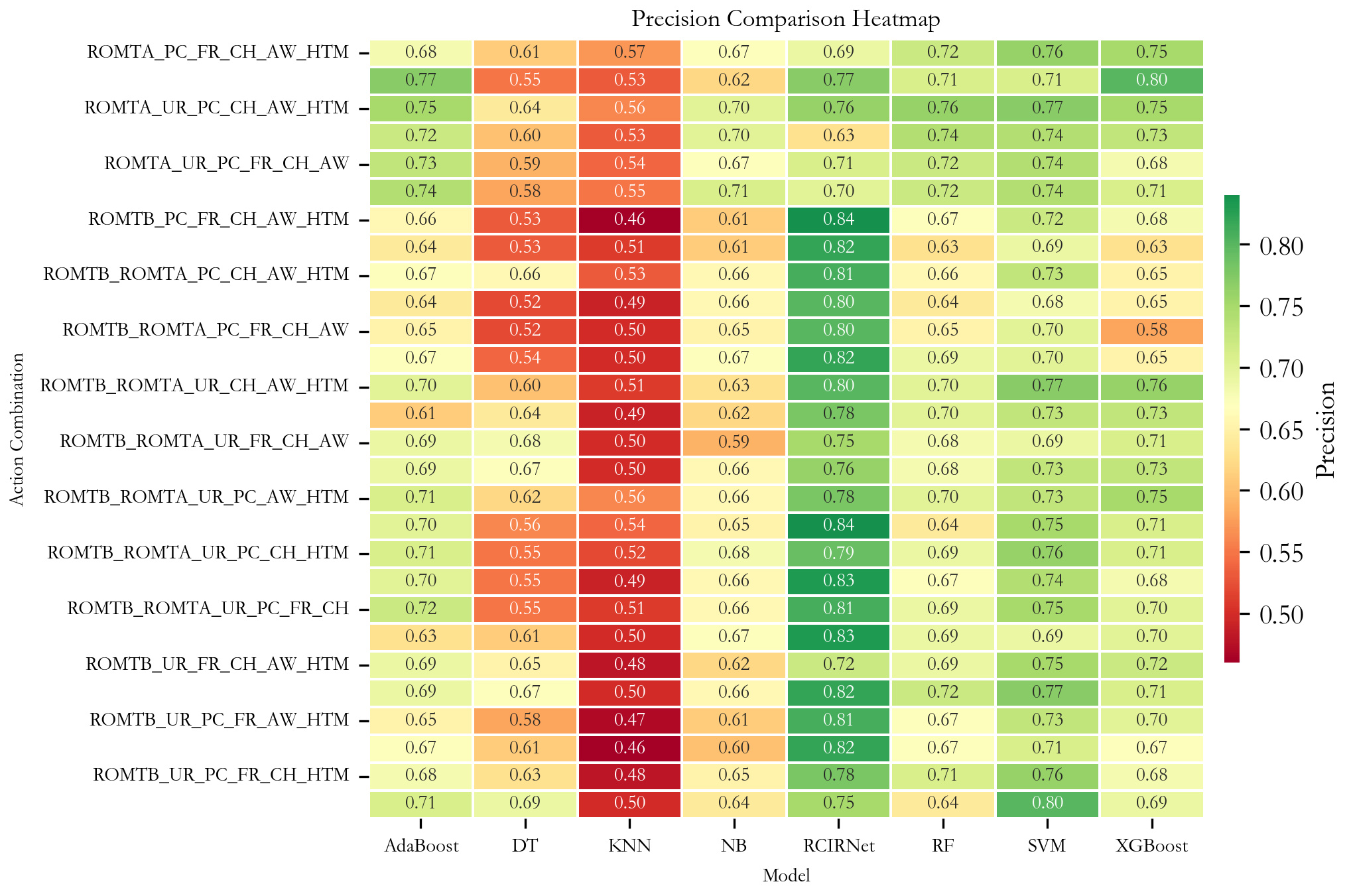


**Supplementary Figure 27.** Precision of six-action combination experiments.


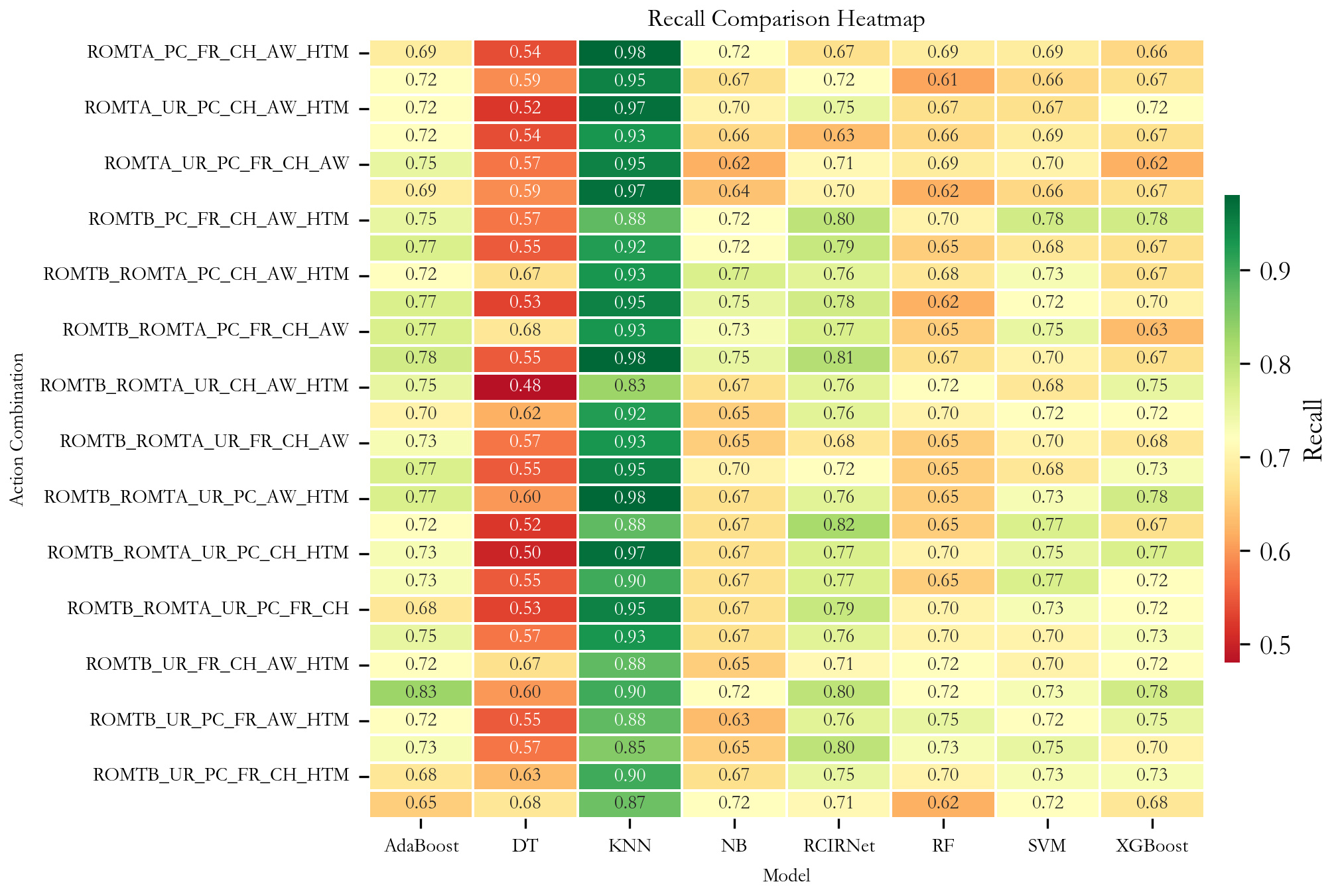


**Supplementary Figure 28.** Recall of six-action combination experiments.


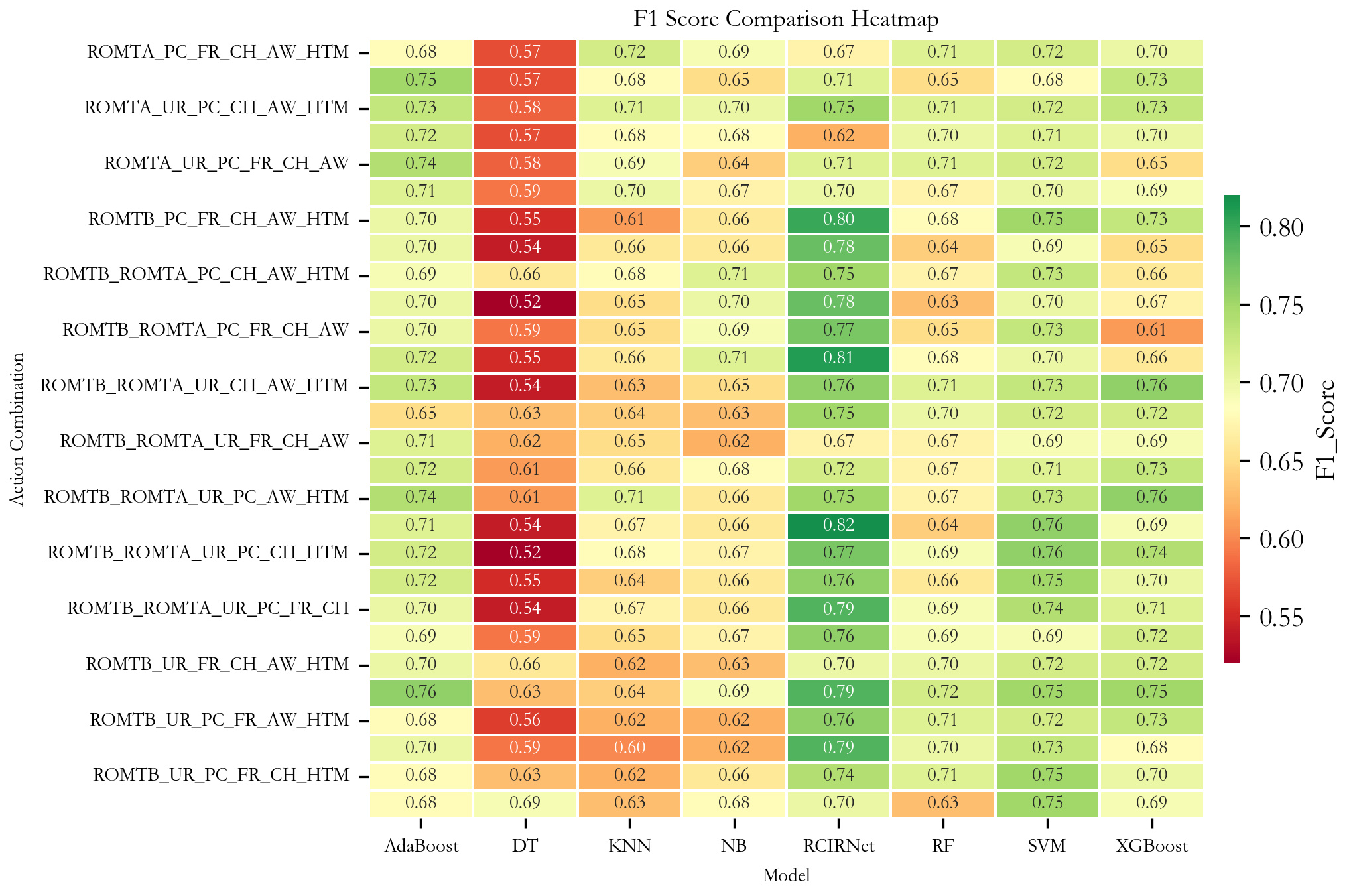


**Supplementary Figure 29.** F1-score of six-action combination experiments.


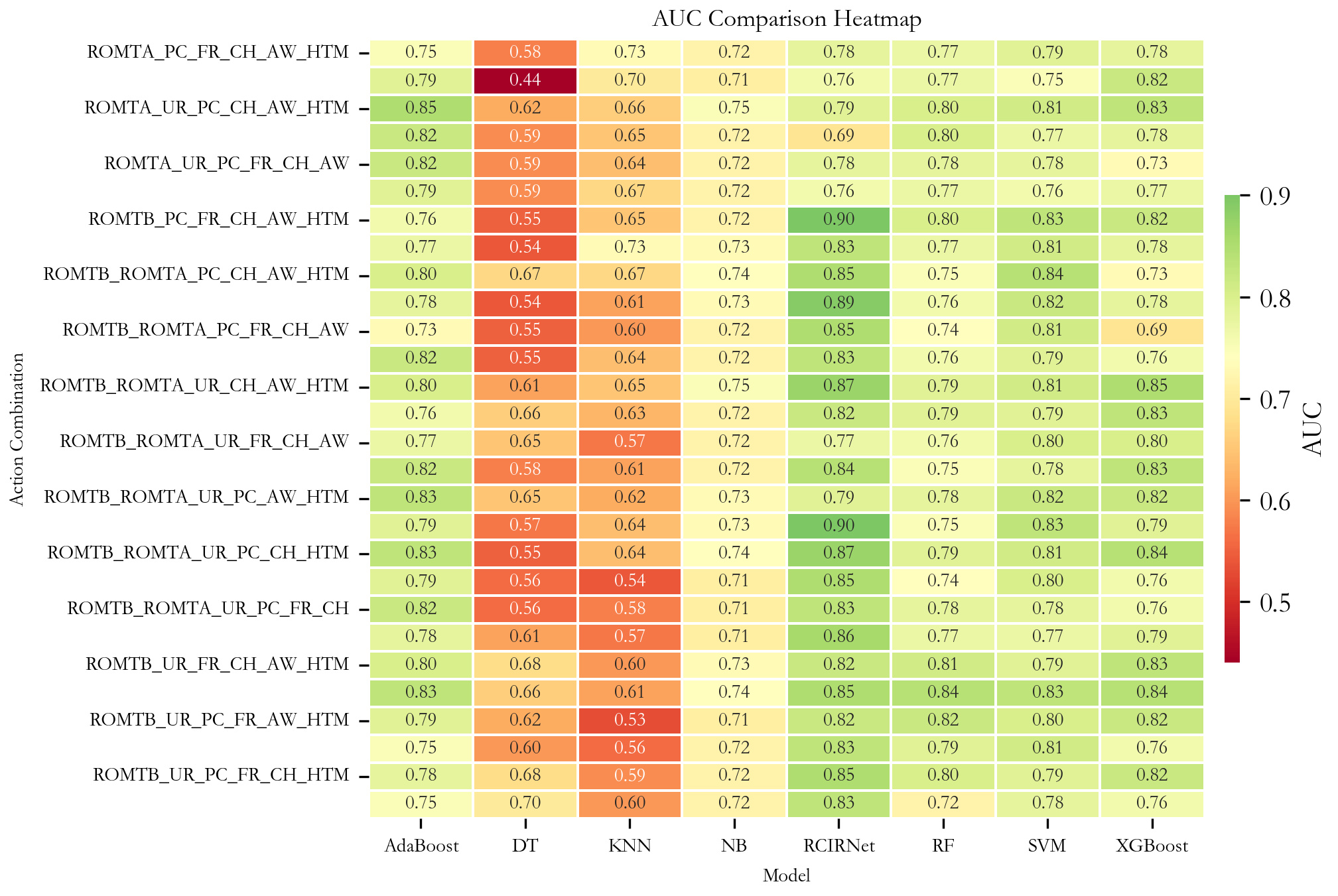


**Supplementary Figure 30.** AUC of six-action combination experiments.

Figures 31-35 present experimental results for RCI recognition based on seven-motion combinations. It can be observed that the classification Accuracy, F1-score, and AUC range from 0.43 to 0.81, 0.53 to 0.81, and 0.55 to 0.89, respectively.


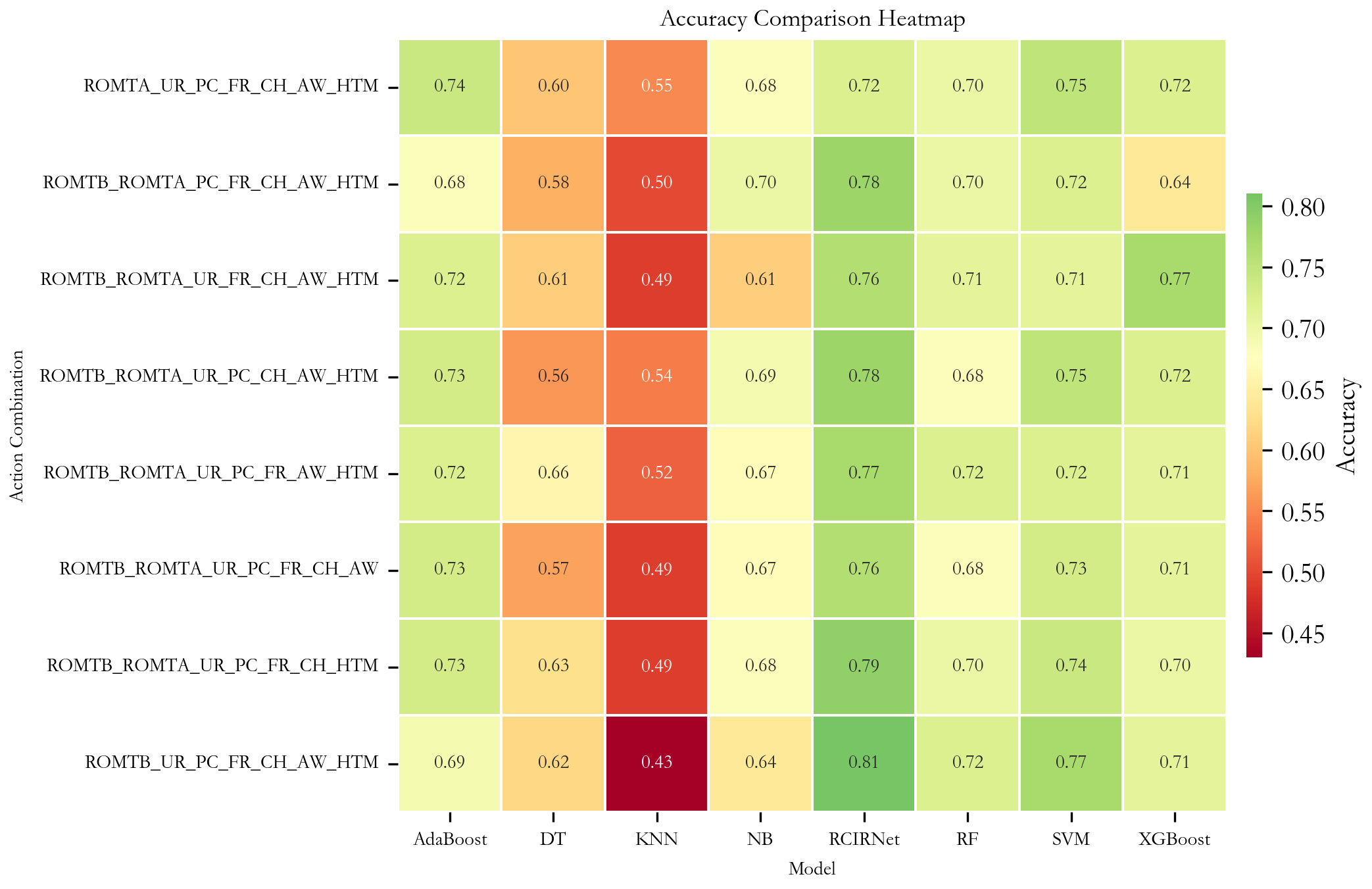


**Supplementary Figure 31.** Accuracy of seven-action combination experiments.


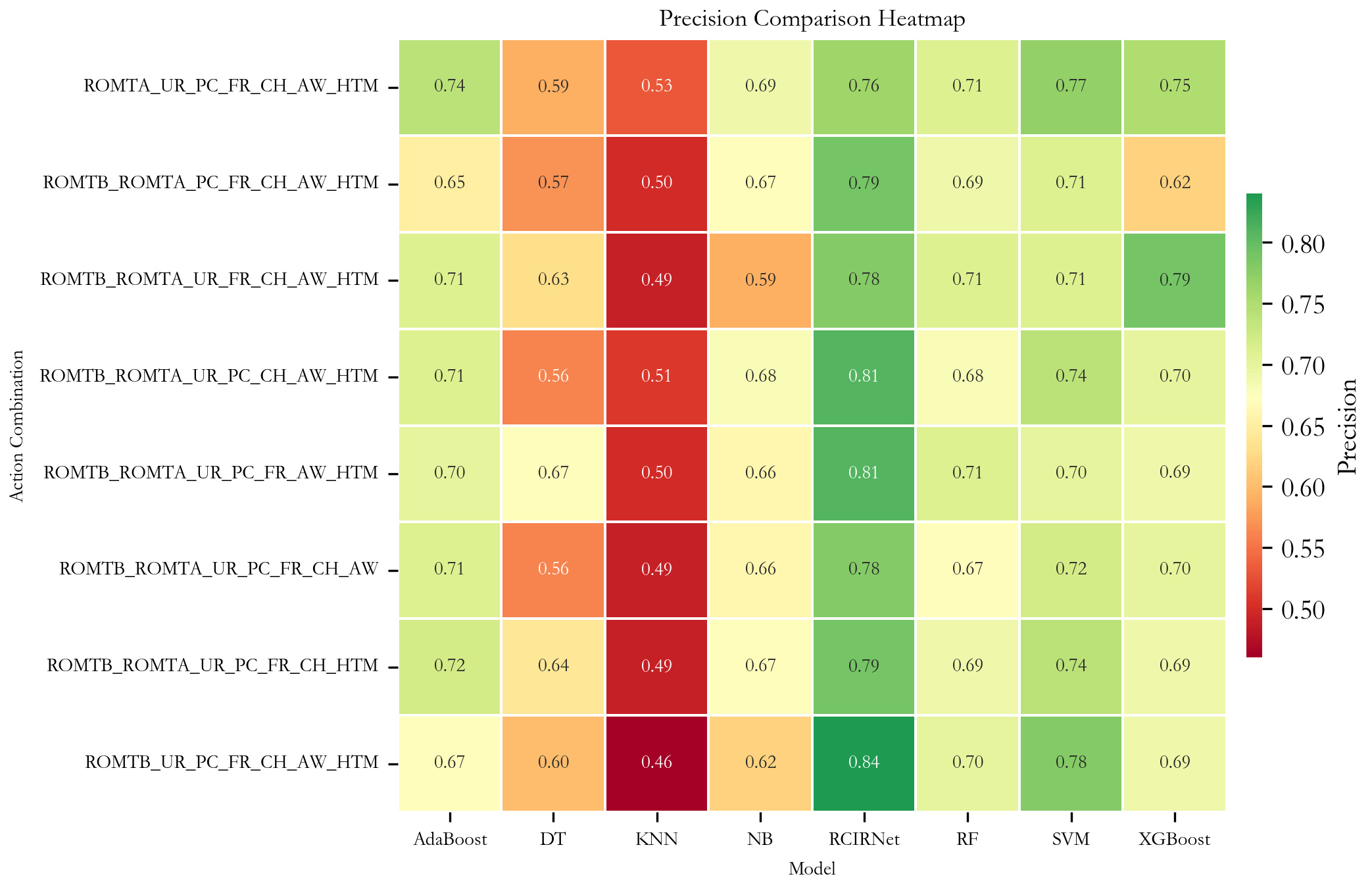


**Supplementary Figure 32.** Precision of seven-action combination experiments.


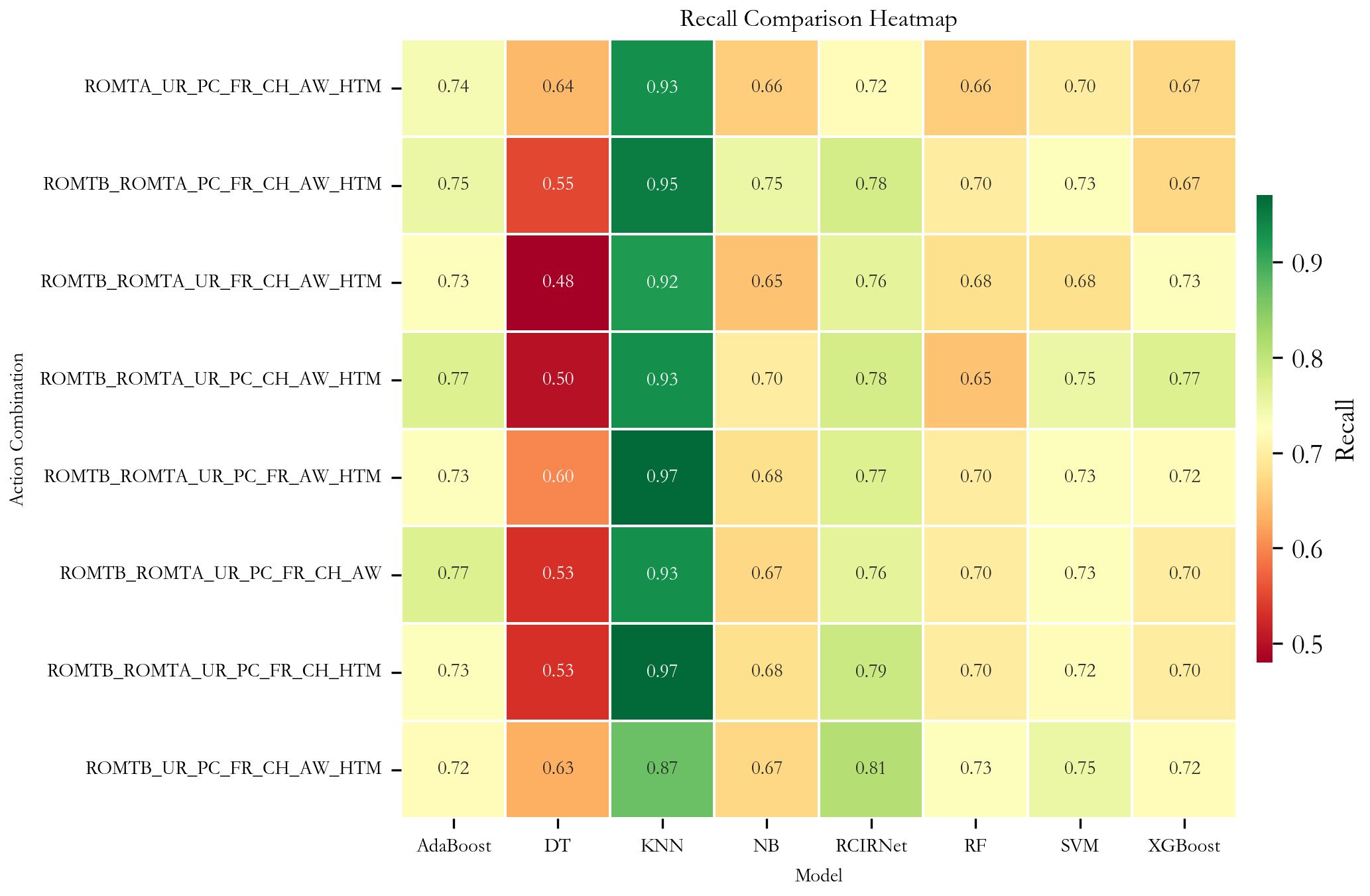


**Supplementary Figure 33.** Recall of seven-action combination experiments.


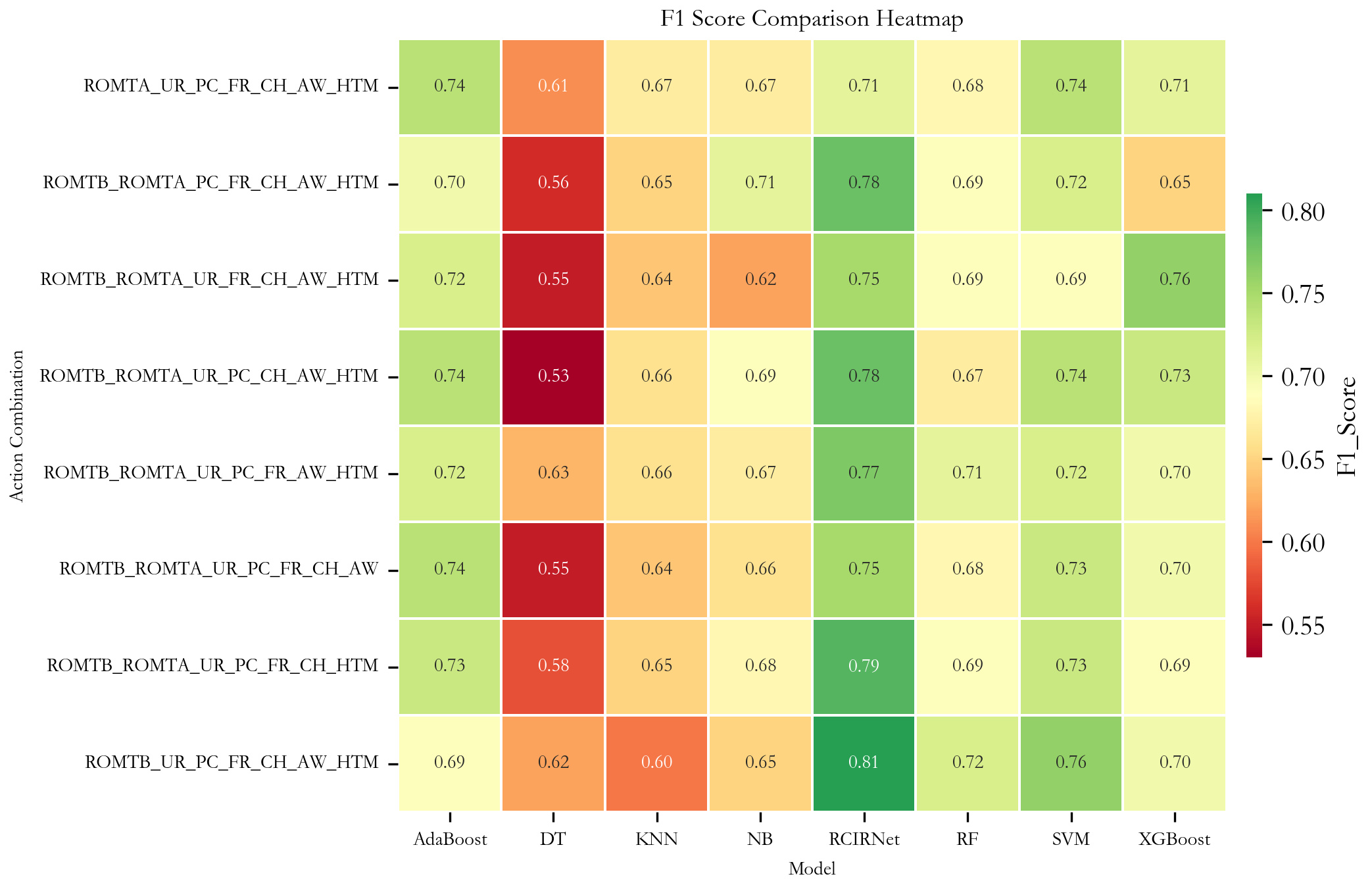


**Supplementary Figure 34.** F1-score of seven-action combination experiments.


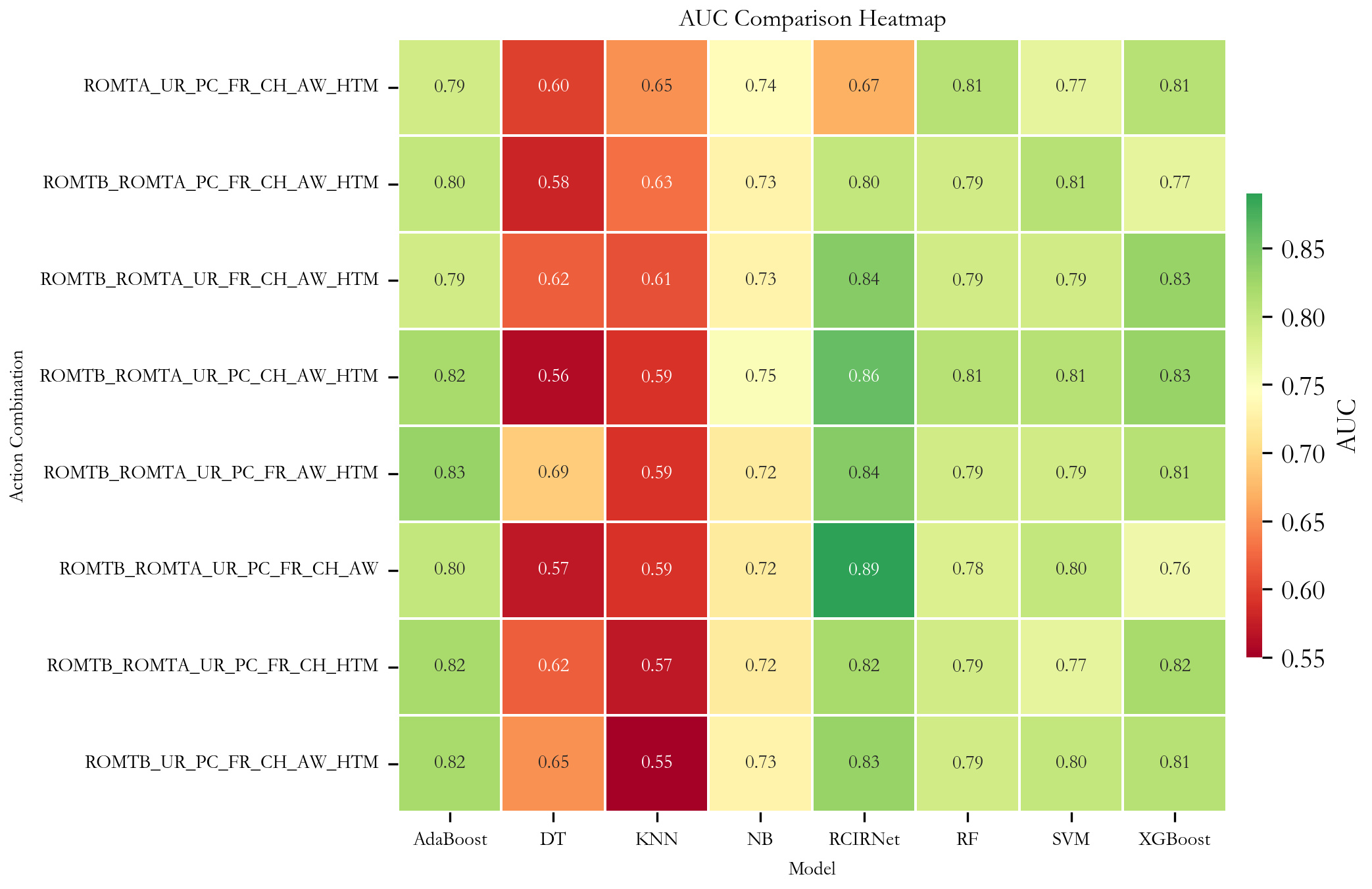


**Supplementary Figure 35.** AUC of seven-action combination experiments.

Figure 36 presents RCI recognition results for eight combined movements. The results indicate that Accuracy, F1-score, and AUC ranged from 0.48 to 0.78, 0.55 to 0.78, and 0.57 to 0.84, respectively.


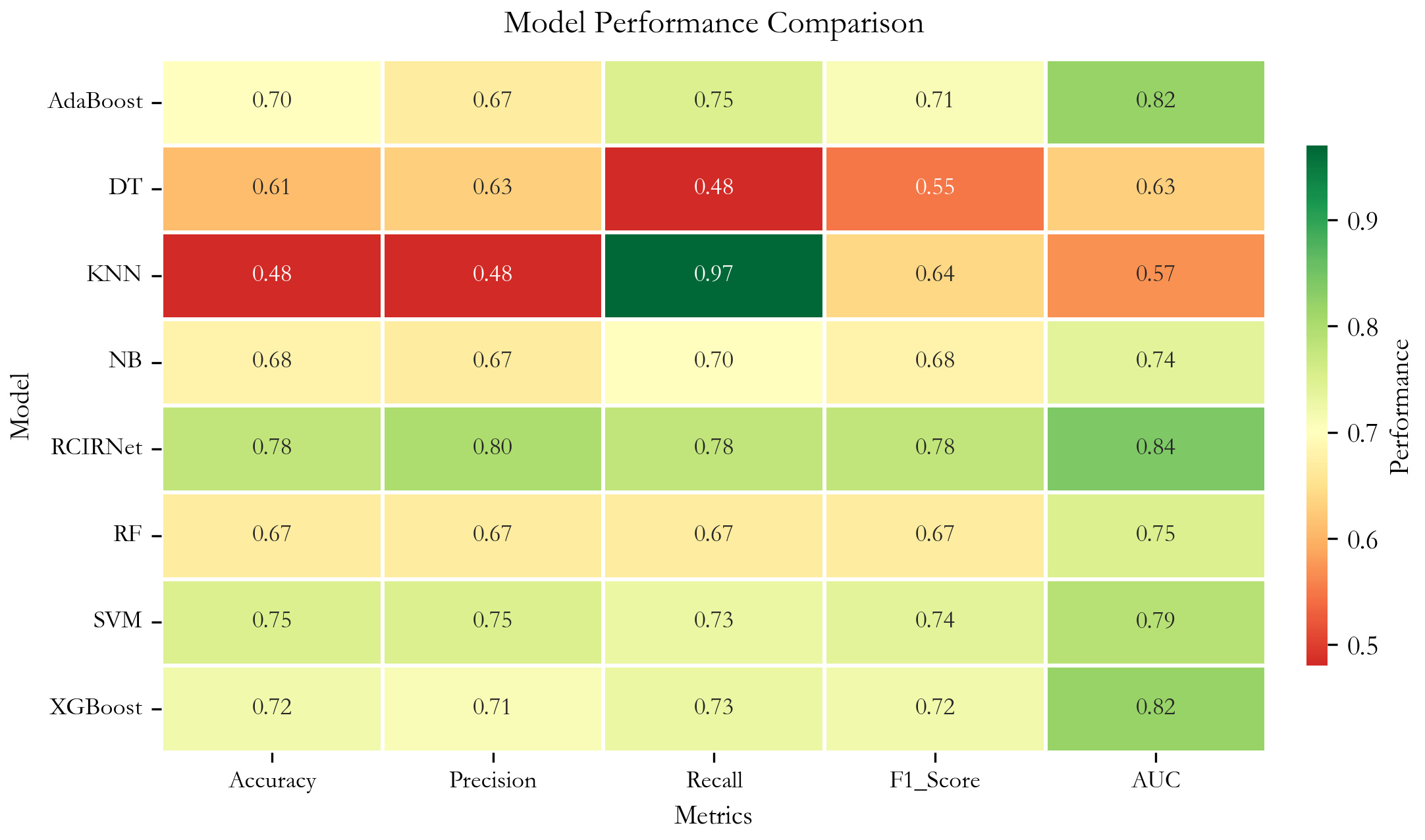


**Supplementary Figure 36.** Results of eight-action combination experiments.

The result of RCIRNet using Leave-One-Subject-Out (LOSO) cross-validation is in Table 1. The recognition Accuracy, F1-score, and AUC ranged from 0.58-0.76, 0.61-0.77, and 0.62-0.79.

**Supplementary Table 1.** Performance of RCIRNet under LOSO cross-validation for single actions.

| Action | Accuracy | Precision | Recall | F1-score | AUC |
| --- | --- | --- | --- | --- | --- |
| ROMTB | 0.72 | 0.72 | 0.73 | 0.72 | 0.77 (0.72,0.81) |
| AW | 0.64 | 0.64 | 0.64 | 0.64 | 0.70 (0.66,0.75) |
| CH | 0.64 | 0.66 | 0.58 | 0.62 | 0.72 (0.67,0.76) |
| FR | 0.58 | 0.57 | 0.65 | 0.61 | 0.62 (0.57,0.67) |
| HTM | 0.62 | 0.62 | 0.64 | 0.63 | 0.67 (0.63,0.72) |
| PC | 0.61 | 0.60 | 0.66 | 0.63 | 0.65 (0.61,0.70) |
| ROMTA | 0.67 | 0.67 | 0.70 | 0.68 | 0.74 (0.70,0.78) |
| UR | 0.76 | 0.76 | 0.78 | 0.77 | 0.79 (0.75,0.83) |

Five DL baselines were implemented for comparison using the best action ROMTB, including CNN, long short-term memory (LSTM), CNN-LSTM, [temporal convolutional neural](https://link.springer.com/article/10.1007/s00500-020-04954-0) (TCN), and Transformer.

The CNN baseline contained three 1D convolutional layers with output channels of 32, 64, and 128 and kernel sizes of 7, 5, and 3, respectively. The first two convolutional layers were each followed by BN, ReLU, and max pooling. The third convolutional layer was followed by BN and ReLU. AdaptiveAveragePool and AdaptiveMaxPool were then applied to obtain global temporal features. The pooled features were concatenated and passed to a fully connected classifier.

The LSTM baseline consisted of a two-layer bidirectional LSTM. The input dimension was 9, corresponding to the nine IMU channels. The hidden size was set to 64, and the dropout rate was set to 0.3. The last hidden states from the forward and backward directions were concatenated. The concatenated feature vector was then passed to a fully connected classifier.

The CNN-LSTM baseline first used two 1D convolutional layers to extract local temporal features. The output channel numbers were 32 and 64, and the kernel sizes were 7 and 5, respectively. Each convolutional layer was followed by BN, ReLU, and max pooling. The extracted feature sequence was then fed into a one-layer bidirectional LSTM with 64 hidden units. The final forward and backward hidden states were concatenated and used for classification.

The TCN baseline consisted of three residual temporal convolution blocks. The output channel numbers of the three blocks were 32, 64, and 128, respectively. Each block contained two 1D convolutional layers, BN, ReLU, dropout, and a residual connection. The kernel size was set to 3. The dilation rates of the three blocks were 1, 2, and 4, respectively. When the input and output channel dimensions were different, a 1 × 1 convolution was used in the residual branch. AdaptiveAveragePool and AdaptiveMaxPool were applied after the temporal convolution blocks. The resulting features were concatenated and passed to a fully connected classifier.

The Transformer baseline first used a 1D convolutional embedding layer to project the nine-channel IMU input into a 64D feature space. This embedding layer included convolution, BN, and ReLU. AdaptiveAveragePool was then used to reduce the sequence to 256 tokens. Learnable positional embeddings were added to the token sequence. The Transformer encoder contained two encoder layers. Each layer used four attention heads, a feed-forward dimension of 128, and a dropout rate of 0.3. The encoded token features were averaged over the temporal dimension. The averaged representation was then passed to a classifier composed of layer normalization and fully connected layers.

This experiment was conducted on the action ROMTB. The results were shown in Table 2. RCIRNet achieved the highest Accuracy (0.89), Precision (0.85), F1-score (0.89) and AUC (0.93). TCN gained the optimal Recall of 0.95, but with a low Precision of 0.77. The results demonstrated that RCIRNet had the best overall performance among the evaluated DL models.

**Supplementary Table** **2.** Performance comparison between RCIRNet and representative deep learning baselines.

| Model | Accuracy | Precision | Recall | F1-score | AUC (95%CI) |
| --- | --- | --- | --- | --- | --- |
| RCIRNet | 0.89 | 0.85 | 0.93 | 0.89 | 0.93 (0.88,0.98) |
| CNN | 0.76 | 0.71 | 0.85 | 0.77 | 0.83 (0.75,0.90) |
| LSTM | 0.63 | 0.62 | 0.60 | 0.61 | 0.64 (0.55,0.74) |
| CNN_LSTM | 0.70 | 0.67 | 0.72 | 0.69 | 0.71 (0.62,0.80) |
| TCN | 0.84 | 0.77 | 0.95 | 0.85 | 0.90 (0.85, 0.96) |
| Transformer | 0.80 | 0.77 | 0.83 | 0.80 | 0.91 (0.86, 0.96) |
